# Circulating retinol binding protein 4 levels in coronary artery disease: a systematic review and meta-analysis

**DOI:** 10.1186/s12944-021-01516-7

**Published:** 2021-08-21

**Authors:** Hengying Chen, Jiaying Zhang, Jiayu Lai, Yingyu Zhou, Xiaoping Lin, Guifang Deng, Zheqing Zhang, Liping Li

**Affiliations:** 1 Injury Prevention Research Center, Shantou University Medical College, Shantou, China; 2 School of Public Health, Shantou University, Shantou, China; 3 Department of Nutrition and Food Hygiene, Guangdong Provincial Key Laboratory of Tropical Disease Research, School of Public Health, Southern Medical University, Guangzhou, China; 4 Department of Clinical Nutrition, Huazhong University of Science and Technology Union Shenzhen Hospital, Shenzhen, China

**Keywords:** Retinol binding protein 4, Coronary artery disease, Ischemic heart disease, Systematic review, Meta-analysis

## Abstract

**Background:**

Retinol binding protein 4 (RBP4) has been proposed to play a role in the pathophysiology of coronary artery disease (CAD), but previous findings on the association of RBP4 levels with CAD are inconsistent.

**Methods:**

A meta-analysis based on observational studies was conducted to evaluate the association between circulating RBP4 levels and CAD. Databases including PubMed, Web of Science, Embase, Google Scholar and ClinicalTrials.gov database were searched for eligible studies published up to 12 July 2021. Standard mean differences (SMDs) with 95% confidence intervals (CIs) were calculated using the inverse variance heterogeneity (IVhet) and random-effects model for data with moderate and high heterogeneity (*I*^*2*^ > 30%) and data with low heterogeneity were analysed using a fixed-effects model (*I*^*2*^ ≤ 30%). Moreover, a bias-adjusted quality-effects model was generated, and the prediction interval was also calculated under the random-effects model.

**Results:**

Two nested case-control studies, one cohort study and twelve case–control studies with a total of 7111 participants were included. Circulating RBP4 levels in patients with CAD were comparable to those in the controls under the IVhet model (SMD: 0.25, 95% CI: − 0.29-0.79, *I*^*2*^: 96.00%). The quality-effects model produced consistent results. However, the association turned to be significant under the random-effect model (SMD: 0.46, 95% CI: 0.17–0.75, *I*^*2*^: 96.00%), whereas the 95% predictive interval (PI) included null values (95% PI: − 0.82-1.74). Subgroup analyses illustrated a positive relationship between CAD and RBP4 levels in patients with complications (SMD: 1.34, 95% CI: 0.38–2.29, *I*^*2*^: 96.00%). The meta-regression analysis revealed that the mean BMI of patients (*P* = 0.03) and complication status (*P* = 0.01) influenced the variation in SMD.

**Conclusions:**

There was low-quality evidence that patients with CAD exhibited similar circulating RBP4 levels compared with controls, and high inter-study heterogeneity was also observed. Thus, RBP4 might not be a potential risk factor for CAD. Comparisons among different subtypes of RBP4 with larger sample size are needed in the future.

**Supplementary Information:**

The online version contains supplementary material available at 10.1186/s12944-021-01516-7.

## Introduction

Coronary artery disease (CAD), the most frequent cardiovascular disease, is the leading cause of mortality and morbidity worldwide [1]. In 2017, 126 million individuals were affected by CAD, and approximately 9 million deaths were attributed to CAD [2]. However, a large number of patients with CAD and a poor prognosis of CAD did not present the traditional risk factors including obesity, diabetes, dyslipidaemia, hypertension and a family history of CAD [3]. The identification of new risk factors and their use as targets for drug or nutrition therapy has been an interesting area of research that has facilitated the prevention and treatment of CAD.

RBP4, a plasma transport protein that delivers retinol from liver to the tissues, may play an important role in CAD through its involvement in the progression of inflammatory mechanisms in adipose and vascular tissues [4]. In vitro studies showed that elevated RBP4 levels promoted aberrant vascular smooth muscle cell proliferation and migration, which contributed to the formation of atherosclerotic plaques [5]. Moreover, RBP4 activated cholesterol uptake to enhance foam cell formation, thereby accelerating the progression of atherosclerosis [6]. RBP4 has received increasing attention over the past few years, with numerous epidemiological studies investigating the relationship of RBP4 levels with the risk of CAD. However, researchers have not clearly determined whether increased RBP4 levels are associated with the risk of CAD, as most studies have reported similar results that elevated circulating RBP4 levels are related to CAD [7–11], whereas a nonsignificant or even negative relationships between RBP4 levels and CAD were documented in other studies [12–14]. Recently, one study reported that serum RBP4 levels were negatively associated with CAD in men but not in women, revealing that hormone-related factors might influence the effect of RBP4 on the development of CAD [15]. The conflicting results may be due to a lack of power in some studies or different sex compositions of the examined populations. Subsequently, a meta-analysis was conducted to assess the relationship between circulating RBP4 levels and CAD, but the study population was limited to Chinese individuals, which restricted the generalization of the results to other populations [16]. Additionally, more updated analyses have been published since the previous meta-analysis [17]. Therefore, a systematic review and meta-analysis of observational studies was conducted to verify whether RBP4 levels were altered in CAD patients compared with the controls.

## Method

### Methods and literature search

The present meta-analysis was conducted in accordance with the guidelines of Meta-analysis of Observational Studies in Epidemiology [18] and Preferred Reporting Items for Systematic Reviews and Meta-Analyses (PRISMA; Supplementary Table 1) [19]. The study protocol was registered at PROSPERO (https://www.crd.york.ac.uk/prospero/) with number CRD42020152286. A systematic literature search was performed in four different databases including PubMed, Embase, Web of Science, Google Scholar and ClinicalTrials.gov database for published and unpublished literature using the following keywords with respect to specific search tips of each database: (“retinol binding protein 4[All Fields]” OR “RBP4[All Fields]” OR “retinol binding protein 4, human[MeSH]”) AND (“coronary artery disease[All Fields]” OR “CAD[All Fields]” OR “coronary heart disease[All Fields]” OR “CHD[All Fields]” OR “coronary artery disease[MeSH]” OR “myocardial infarction[MeSH]” OR “myocardial infarction[All Fields]” OR “ischemic heart disease[All Fields]” OR “myocardial ischemia[MeSH]” OR “myocardial ischemia[All Fields]” OR “angina pectoris[All Fields]” OR “angina pectoris[MeSH]”). The details of search strategy were shown in Supplementary Table 2. The last search was performed on 12 July 2021. Reference lists of the identified papers were also searched for potential relevant publications.

### Study selection

The selection criteria were listed below: (1) case–control, nested case–control or prospective cohort studies that investigated the relationship of the circulating RBP4 concentration with the risk of CAD; (2) mean circulating RBP4 level and its standard deviation, or adjusted odds ratio (OR), relative risk (RR) or hazard ratio (HR) with 95% CIs (confidence intervals) were reported; (3) studies were excluded if its published language is not English; (4) studies focused on a population with a disease such as renal disease and nonalcoholic fatty liver disease (NAFLD) were also excluded; (5) The publication with the largest sample size was considered eligible when there were multiple publications from the same study population. The CAD was defined as stable angina, unstable angina, myocardial infarction and sudden cardiac death. The diagnosis of CAD was based on the standards applied in all original articles. Studies with more than one subtype of CAD were regarded as two or more studies sharing the control group. Two investigators (HY Chen and JY Zhang) independently screened the titles and abstracts of records, and disagreements between investigators were resolved by consensus. Then, these investigators read full texts and confirmed studies for final inclusion with a third reviewer (ZQ Zhang).

### Data extraction

The necessary data were extracted independently by two researchers (HY Chen and JY Zhang) using a purpose-designed form. Any discrepancy was resolved by discussion or involved a third reviewer (ZQ Zhang) when necessary. Data were extracted from graphs using R package metaDigitise [20]. The following information were extracted from each eligible study: the first author’s name, publication year, sample size, study design, origin of the study population, clinical classification of CAD, method of measurement, general characteristics of participants (age, sex, and BMI), mean circulating RBP4 concentration and its corresponding standard deviation (SD) or standard error for patients and controls, risk estimates (RR, OR, or HR), 95% CIs and confounders adjusted for in the final models. If the standard deviation of the RBP4 concentration was lacking, it was calculated from the provided standard error of the mean or 95% CIs or other basic parameters [21].

### Quality assessment and strength of evidence across studies

The risk of bias was assessed using the Risk of Bias In Non-Randomized Studies of Exposure (ROBINS-E) tool [22]. The Grading of Recommendations Assessment, Development and Evaluation (GRADE) approach was utilized to evaluate the certainty of the evidence [23]. Evidence from observational studies with default low-certainty levels can be downgraded or upgraded according to the assessment of limitations in five domains: study design, indirectness, imprecision, inconsistency and reporting bias.

### Statistical analysis

The means and SD of circulating RBP4 levels in CAD and control groups and risk estimates were used to evaluate the effect size for each study. SMD presented as Cohen’s *d* and a summary OR with 95% CI were calculated to estimate the association of circulating RBP4 levels with CAD [24]. RRs were considered equivalent to ORs [25]. A random-effects model and inverse variance heterogeneity (IVhet) model were utilized if moderate or high heterogeneity were observed (*I*^*2*^ ≥ 30%); otherwise, a fixed-effects model was utilized as noted [26]. However, the IVhet model preserving a correct coverage probability and showing a lower observed variance independent of heterogeneity performs better than the random-effect model [27]. A quality-effects model was also performed to adjust for bias [28]. In addition, the prediction interval (PI) was calculated using the Higgins’s method to account for between-study heterogeneity and evaluate the certainty of the association if new studies were conducted in the future [17, 29]. Subgroup meta-analyses were conducted of patients stratified by mean age of patients (< 60 years old or ≥ 60 years old), origin of the study population (Asian or others), mean BMI of patients (< 25 kg/m^2^ or ≥ 25 kg/m^2^), complication status (CAD alone or CAD with complications) and percentage of female subjects (≥50% or < 50%) to explore potential explanations for the heterogeneity. Meta-regression analyses were applied for exploring the possible sources of heterogeneity. The effect size was set as the dependent variable with geographic region (Asian or others), mean age of patients (< 60 years old or ≥ 60 years old), mean BMI of patients (< 25 kg/m^2^ or ≥ 25 kg/m^2^), female percentage among patients (≥50% or < 50%), quality score (continuous) and complication status (CAD alone or CAD with complications) as explanatory variables. Sensitivity analyses were also performed by sequentially omitting each study to evaluate the robustness of the principal findings. Additionally, a cumulative meta-analysis was performed to monitor the evidence over time. Finally, publication bias was detected using Luis Furuya-Kanamori (LFK) index and the Doi plot [30]. The absolute value of LFK index < 1 was categorized as ‘symmetry’. All analyses were conducted using Stata 16.0 software (Stata Corporation, Texas) and MetaXL (version 5.3, EpiGear International Brisbane). All *P* values were two-sided, and a *P* value < 0.05 was considered statistically significant.

## Results

### Literature search

**Fig. 1:**
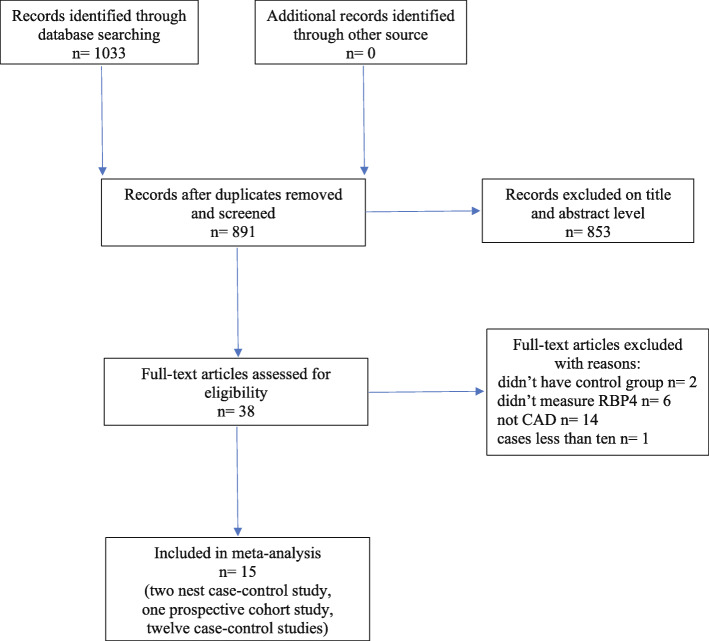
Flow chart of study selection in the meta-analysis

A flow chart of the study selection progress is presented in Fig. 1. A total of 1033 potential publications were identified in the systematic search of four different databases. A total of 891 articles remained for screening the title and abstract after removing the duplicates. Of these articles, 853 studies were excluded after screening at the titles and abstracts, leaving 38 studies for full-text inspection. Twenty-three articles were subsequently excluded at the full-text review phase, of which 2 articles did not include a control group, 6 articles did not measure RBP4 levels, 14 articles were related to other diseases and 1 article published results from fewer than 10 patients (Supplementary Table 3). Therefore, 15 articles that met the inclusion criteria were pooled in the analysis [7–15, 31–36].

### Study characteristics

Table 1 provides an overview of selected characteristics of the eligible studies. The selected articles were published between 2007 and 2020, including 3471 patients with CAD and 3640 controls. These studies included a prospective cohort study [8], 12 case–control studies [7, 9–15, 31, 32, 34, 36] and two nested case–control studies [33, 35]. Studies were conducted in Asia [8–11, 13–15, 32, 34, 36], Europe [7, 12, 31, 35] and the USA [33]. Patients with CAD were aged 55.2 to 64.7 years, and the BMI covered a range of 23.0 to 32.7 kg/m^2^ across studies. Levels of RBP4 were determined using an ELISA in all of the studies. Most studies enrolled both female and male participants [7, 9–11, 13–15, 32, 34–36], while two studies recruited only males [12, 31] and two studies involved solely females [8, 33]. The comparative data between CAD and control group was provided as follows: 11 studies, CAD (*n* = 3169) vs controls (*n* = 3369); two studies, CAD with complications group (*n* = 177) vs controls (*n* = 141); and two studies, CAD alone and CAD with complications group (*n* = 125) vs controls (*n* = 130).

**Table 1:** Main characteristics of the studies included in present meta-analysis

References (Author, Year)	Location	Study design	Method of RBP4 measurement	Clinical classification	Age in cases (years)	Female in cases (%)	BMI in cases (kg/m^2^)	RBP4 units	Cases		Controls	
									n	Mean ± SD	n	Mean ± SD
von Eynatten et al., 2007 [31]	Germany	Case-control	ELISA	CAD	62.0	0.0	27.6	μg/ml	143	28.08 ± 8.54	96	31.38 ± 10.46
Mallat et al., 2008 [35]	France	Nest case-control	ELISA	CAD	64.7	36.2	27.2	mg/L	1036	48.90 ± 16.80	1889	46.80 ± 14.30
Al-Daghri et al., 2009 [10]	Saudi Arabia	Case-control	ELISA	CAD + DM	56.0	51.7	32.7	mg/L	29	33.40 ± 13.60	39	22.60 ± 9.50
Mahmoudi et al., 2012 [32]	Iran	Case-control	ELISA	CAD	55.2	0.0	29.8	μmol/L	15	1.30 ± 0.29	15	1.24 ± 0.15
Sun et al., 2013 [33]	USA	Nest case-control	ELISA	CAD	59.5	100.0	26.5	μg/ml	468	97.91 ± 43.72	472	94.56 ± 43.57
Cubedo et al., 2014 [12]	Spain	Case-control	ELISA	AMI	NA	0.0	NA	μg/ml	68	32.00 ± 10.72	132	39.70 ± 10.34
Lambadiari et al., 2014 [7]	Greece	Case-control	ELISA	CAD	64.0	11.8	28.9	mg/L	305	39.29 ± 11.72	91	24.83 ± 11.27
Li et al., 2014 [34]	China	Case-control	ELISA	CAD	63.7	36.7	23.0	μg/ml	30	17.66 ± 15.32	30	17.49 ± 24.45
–	–	–	–	CAD + HIns	63.7	41.4	25.0	μg/ml	29	26.67 ± 29.71	–	–
Liu et al., 2015 [8]	China	Prospective	ELISA	Stable CAD	NA	100.0	NA	μg/ml	201	39.31 ± 15.12	225	25.57 ± 6.59
Guan et al., 2016 [11]	China	Case-control	ELISA	CAD	63.7	45.7	NA	mg/L	35	18.39 ± 6.61	35	16.54 ± 4.34
–	–	–	–	CAD + HIns	63.6	45.2	NA	mg/L	31	27.36 ± 8.32	–	–
Wang et al., 2018 [15]	China	Case-control	ELISA	CAD	63.7	39.8	24.2	μg/ml	440	28.99 ± 10.87	218	31.44 ± 9.96
Liu et al., 2019 [14]	China	Case-control	ELISA	CAD	60.5	25.0	25.5	ng/ml	180	5.70 ± 1.80	79	3.60 ± 2.40
Sun et al., 2019 [9]	China	Case-control	ELISA	SCH + CAD	64.4	53.8	25.0	μg/ml	148	58.29 ± 13.86	102	35.07 ± 10.44
Pan et al., 2020 [13]	China	Case-control	ELISA	AMI	NA	NA	NA	ng/ml	150	35.99 ± 20.34	75	44.67 ± 29.63
Si et al., 2020 [36]	China	Case-control	ELISA	CAD	57.0	26.4	25.5	ng/ml	163	5.57 ± 2.57	77	4.89 ± 2.70

*RBP4* retinol binding protein 4, *CAD* coronary artery disease, *SCH* Subclinical hypothyroidism, *DM* diabetes mellitus, *AMI* acute myocardial infarction, *HIns* hyperinsulinemia, *USA* united states of America, *SD* standard deviation, *BMI* body mass index, *ELISA* enzyme linked immunosorbent assay

### Quality assessment

The results of the risk of bias assessment using the ROBINS-E tool are shown in Supplementary Table 4. All of the eligible studies were judged as moderate risk of bias due to confounding. The risks of bias due to departures from intended exposures, bias due to missing data, bias in measurement of outcomes and bias in selection of the reported result were considered low in all the studies. Regarding the bias in selection of participants into the study, three studies [8, 33, 35] were considered to be at low risk, and twelve studies [7, 9–15, 31, 32, 34, 36] were considered to be at moderate risk. In relation to the bias in classification of exposures, two articles [35, 36] were categorized as having a moderate risk of bias and thirteen articles [7–15, 31–34] were at low risk.

### Main analysis

**Fig. 2:**
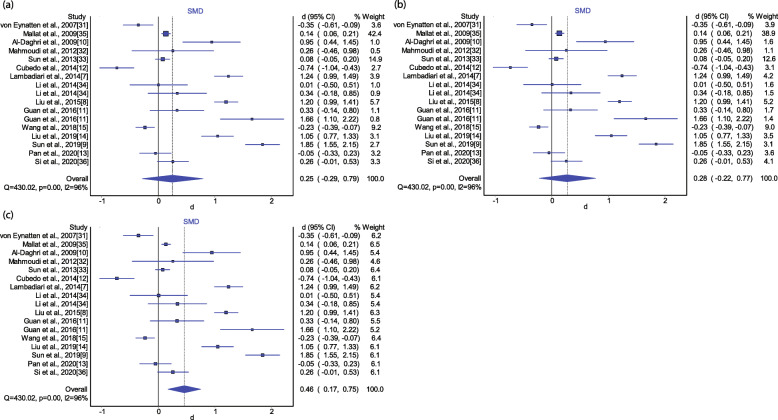
RBP4 levels in CAD patients compared with controls. **a** IVhet model; **b** quality-effect model; **c** random-effect model

**Fig. 3:**
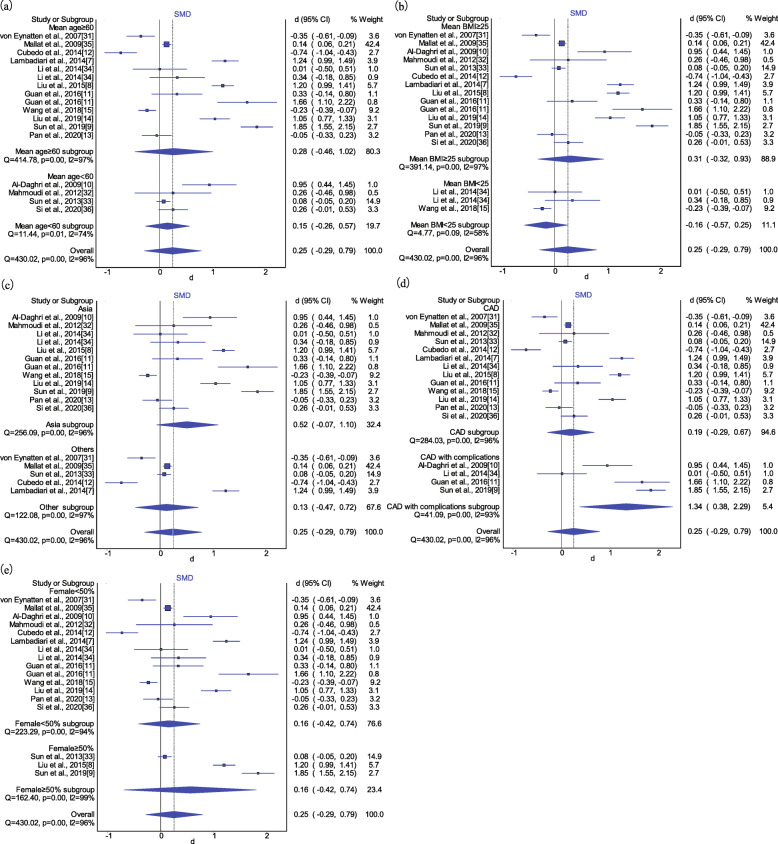
Subgroup analysis of RBP4 levels in CAD patients compared with controls. **a** mean age in cases; **b** mean BMI in cases; **c** region; **d** complication status; **e** female percentage in cases

As significant heterogeneity existed among studies (*I*^*2*^ = 96.00%, *P* < 0.01), the IVhet model and the random-effects model were adopted. As illustrated in Fig. 2, the circulating RBP4 levels were not significantly elevated in patients with CAD when compared with those without CAD (SMD: 0.25, 95% CI: − 0.29-0.79). The result from the quality-effect model was similar (SMD: 0.28, 95% CI: − 0.22-0.77). However, the association between RBP4 levels and CAD turned significant under the random-effect model (SMD: 0.46, 95% CI: 0.17–0.75). The PI ranged from − 0.82 to 1.74, revealing that the 95% plausible range for the mean difference in RBP4 levels compared with the control group would be − 0.82 to 1.74 if a study was conducted in the future. The results of subgroup analyses are presented in Fig. 3. Insignificant associations between circulating RBP4 concentrations and the risk of CAD remained in the subgroup analyses stratified according to mean BMI of patients, mean age of patients, female percentage and geographic region. However, a significant SMD in the RBP4 concentration was observed in studies of CAD patients with a complication (SMD: 1.34, 95% CI: 0.38–2.29), but not CAD patients without a complication (SMD: 0.19, 95% CI: − 0.29-0.67).

### Meta regression

The results of the meta-regression analysis indicted that mean BMI of patients (β = − 0.44, 95% CI: 0.09 to 0.79, *P* = 0.03, *R*^*2*^ = 0.11) and complication status (β = 1.14, 95% CI: 0.30 to 1.99, *P* = 0.01, *R*^*2*^ = 0.24) might exert a significant effect on the pooled effect size, accounting for a low level of heterogeneity (11.00 and 24.00%, respectively). In addition, geographic region (β = − 0.39, 95% CI: − 1.05 to 0.28, *P* = 0.23, *R*^*2*^ = 0.12), mean age of patients (β = 0.10, 95%CI: − 0.27 to 0.47, *P* = 0.56, *R*^*2*^ = 0.01), female percentage (β = 0.41, 95% CI: − 0.46 to 1.28, *P* = 0.33, *R*^*2*^ = 0.11) and quality score (β = − 0.13, 95% CI: − 0.74 to 0.47, *P* = 0.65, *R*^*2*^ = 0.01) failed to account for heterogeneity in the results (Table 2).

**Table 2:** Meta-regression analysis to assess the influence of basal variables on the effect sizes

Variables	*β*	95% CI	*P*	R^2^
The percentage of females	0.41	−0.46, 1.28	0.33	0.11
Mean age	0.10	−0.27, 0.47	0.56	0.01
Mean BMI	0.44	0.09, 0.79	0.03	0.11
Region	−0.39	−1.05, 0.28	0.23	0.12
Quality score	−0.13	−0.74, 0.47	0.65	0.01
Complications status	1.14	0.30, 1.99	0.01	0.24

*CI* confidence interval, *BMI* body mass index

### Cumulative Meta-analysis

**Fig. 4:**
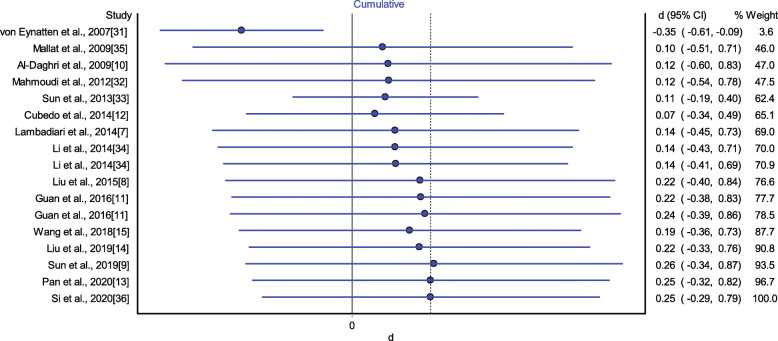
Cumulative meta-analysis of RBP4 levels in CAD patients compared with controls

A cumulative meta-analysis was conducted to estimate the association between RBP4 levels and CAD in relation to the year of publication (Fig. 4). The results indicated that the insignificant association between circulating RBP4 levels and CAD remained stable after the study by Mallat et al. appeared in 2009 [35].

### Sensitivity analysis and publication bias

**Fig. 5:**
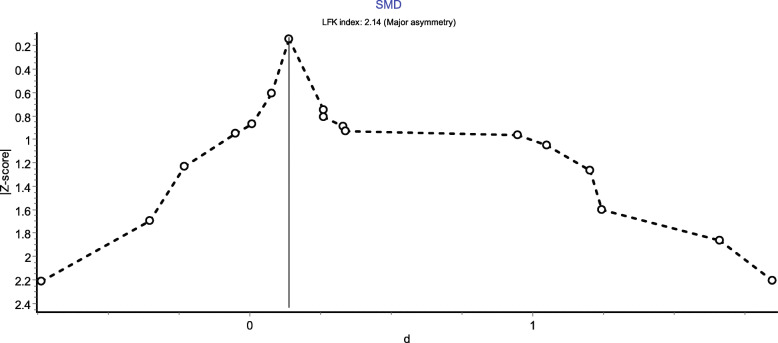
Doi plot for the analysis of RBP4 levels in CAD patients compared with controls

A sensitivity analysis performed by omitting individual studies one by one revealed that no individual study had any undue effects on the pooled results (Supplementary Table 5). Major asymmetry (LFK index: 2.14) was observed in the Doi plot (Fig. 5).

### Certainty of evidence

Based on the GRADE approach, the quality of evidence was rated as ‘very low’ due to the plausible existence of risk of bias of inconsistency and indirectness (Supplementary Table 6).

## Discussion

The present meta-analysis included 15 studies with a total of 7111 participants (patients = 3471 and control = 3640) and used the IVhet and quality-effect models to show comparable circulating RBP4 levels in patients with CAD than in those without CAD. CAD patients with complications are particularly susceptible to having increased RBP4 levels.

A previous meta-analysis by Hou et al. including 10 studies with 1698 Chinese subjects reported a significant difference in RBP4 levels between CAD and non-CAD participants under the random-effect model (weighted mean difference = 7.17 μg/ml, 95%CI:3.56–10.78 μg/ml, *P*<0.05) [16], which is inconsistent with the results of the present meta-analysis. However, the wider population and larger sample size of the current study may improve the stability of the results. Additionally, only the random-effect model was utilized in the previous meta-analysis, but the IVhet model that preserved a correct coverage probability and a true variance irrespective of heterogeneity was also adopted in this meta-analysis. The association between RBP4 concentrations and cardiovascular disease has been investigated in several studies, yielding mixed results. A secondary analysis of a multicentre trial by Wang et al. revealed that subjects with higher RBP4 levels experience an excessive risk of coronary artery calcification [37]. The results of the BCAMS study after a 10-year follow-up have shown that elevated RBP4 levels during childhood had a good ability to predict the cardiometabolic risk in adults [38]. In addition, a positive correlation of RBP4 levels with the multifactorial pathogenesis of metabolic dysregulation was reported in the Framingham Heart Study [39]. In contrast, an inverse association between RBP4 levels and cardiovascular mortality [HR (95% CI) for cardiovascular mortality, tertile 3 versus tertile 1: 0.73 (0.50, 1.07)] was observed among 950 type 2 diabetes patients with 22 years of follow-up [40]. Therefore, the results are mixed, which may be attribute to the use of different methods (e.g., ELISA and western blot) and differences in age, ethnicity, sex composition and sample size.

A potential inflammatory mechanism was suggested to link circulating RBP4 levels to CAD, although the exact biological mechanism is currently unknown. Farjo et al. reported that RBP4 induces vascular inflammation by stimulating the mRNA expression of factors involved in leucocyte recruitment and adhesion to endothelial cells [4]. In addition, elevated RBP4 levels occur in part by the activation of NADPH oxidase and NF-κB, inducing the expression of proinflammatory molecules in both retinal capillary endothelial cells and umbilical vein endothelial cells, and accelerating the formation of atheromatous plaques [41, 42]. In addition, a notable number of studies have shown that elevated circulating RBP4 concentrations are positively correlated with the increase in oxidative stress markers including urinary 8-isoprostane [43] and malondialdehyde [44].

In the present meta-analysis, patients with CAD presenting complications (i.e., hyperinsulinaemia and subclinical hypothyroidism) had a greater effect size than patients with CAD without complications compared with the controls (*P* for the meta-regression analysis = 0.01). Patients with diabetes, NAFLD and other chronic diseases were commonly complicated with CAD [45, 46]. The associations between circulating RBP4 levels, diabetes mellitus and other chronic diseases have been well documented [38, 47]. Complications of CAD may enhance the increase in RBP4 levels, as increasing RBP4 levels were also observed in patients with diabetes and metabolic syndrome [38].

Adipokines including leptin and adiponectin have been found to be sexually dimorphic and be affected by sex hormones [48, 49]. Sex differences in the associations of circulating RBP4 concentrations with insulin resistance and fasting blood glucose levels have been reported [50]. The circulating RBP4 levels in premenopausal women were significantly lower than that in postmenopausal women, indicating the possible influence of the gonadotropins on the expression of RBP4 [51, 52]. A similar situation was observed when comparing RBP4 levels in women aged over 50 years with those younger than 50 years [53]. A decrease in the production of oestrogen during menopause that in turn resulted in increased oxidative stress and subsequently increases in RBP4 level may therefore raise the risk of CAD. In addition, the mean age of patients with CAD ranged from 55.2 to 64.7 years in this study, which may merely cover the postmenopausal period. This characteristic might partially explain the non-sex-specific results. Similar circulating RBP4 levels were detected among different ethnicities [54], which was in line with the findings of the current study. However, the relationship between circulating RBP4 concentrations and insulin sensitivity differed between ethnic groups [55]. The majority of the eligible studies were conducted with one race. Large-sample prospective studies are required to clarify the findings of the present study.

With advancing age and increasing weight, increased systemic inflammation may affect the onset of CAD [56]. According to previous studies, RBP levels are higher in older adults and patients with a higher BMI [9, 34], among whom the risk of CAD is also increased. Meta-regression analyses showed that mean BMI of patients confounded the difference in circulating RBP4 levels between CAD patients and the controls, indicating the potential link between BMI and circulating RBP levels among patients with CAD. However, the age of subjects had a minimal effect on the relationship between RBP4 levels and CAD presented in this study, probably due to the narrow range of ages of the patients enrolled in those studies (range: 55.2–64.7).

The prediction interval was calculated to evaluate the heterogeneity and the certainty of the association for future studies. Accordingly, a wide range (− 0.82 to 1.74) of PI was observed, indicating that RBP4 levels would be either significantly increased or decreased in future studies comparing patients with CAD and healthy controls. These results may reflect the uncertainty of the association between RBP4 levels and CAD risk, highlighting a limitation of epidemiological studies on this topic. Moreover, the risk of bias assessment revealed that most eligible studies had a moderate risk of bias, and the results obtained after adjustment for the risk of bias using the quality-effect model showed that the effects of the risk of bias on pooled effect sizes were limited in the current meta-analysis.

### Strengths and limitations

The current meta-analysis has a much larger sample size and included literature from wider regions including China and other countries, compared with the previous meta-analysis [16]. However, several limitations of the study should also be considered. First, most of the studies did not adjust for potential confounders, such as age, sex and smoking status, reducing the credibility of the results. Second, high between-study heterogeneity was observed, although the IVhet model was used to pool the results. The results of this meta-analysis should be interpreted with caution and considered for hypothesis generation. Third, the SMD was utilized to assess the association of RBP4 levels with CAD instead of the pooled odds ratio because insufficient data was provided in all the included studies (Supplementary Table 7 and Supplementary Fig. 1), which only reflected the difference in expression of RBP4 between patients with CAD and the control. Future studies are needed to explore the potential association between increased circulating RBP4 levels and the CAD risk and the diagnostic value of RBP4 levels for CAD. Fourth, a high proportion of the eligible articles employed a cross-sectional design; thus, the results of the current study only suggested an association instead of causation. Fifth, substantial evidence of publication bias was found using the Doi plot. Sixth, subgroup and meta-analyses using the aggregated levels of patient characteristics may lead to the aggregation bias, namely ecological fallacy [57]. Finally, the association between the levels of different subtypes (i.e., full-length RBP4 and RBP4-L) of RBP4 and CAD failed to be examined, as only one study reported the parameters.

## Conclusions

There was low-quality evidence that circulating RBP4 levels in patients with CAD were comparable to the levels of controls. However, CAD patients with complications were at risk of having higher levels of RBP4. Based on the results, RBP4 may not be a risk factor for the progression of CAD. Comparisons among different subtypes of RBP4 with larger sample size are needed in the future.

## Supplementary Information

**Additional file 1.**

## Data Availability

All data generated or analyzed during this study are included in this article.

## Abbreviations

CAD: Coronary artery disease
RBP4: Retinol-binding protein 4
SD: Standard deviation
SMD: Standard mean difference
BMI: Body mass index
CIs: Confidence intervals
NAFLD: Nonalcoholic fatty liver disease
USA: United states of America
ELISA: Enzyme linked immunosorbent assay
OR: Odd ratio
RR: Relative risk
HR: Hazard ratio
PI: Prediction interval
ROBINS-E: Risk of Bias In Non-Randomized Studies of Exposure
GRADE: The Grading of Recommendations Assessment, Development and Evaluation
IVhet: Inverse variance heterogeneity
