# Supplementary material for: Circulating retinol binding protein 4 levels in coronary artery disease: a systematic review and meta-analysis
Source: Lipids Health Dis. 2021 Aug 21;20:89. doi: 10.1186/s12944-021-01516-7 (PMC8380323; doi:10.1186/s12944-021-01516-7)
Supplement: Supplementary file 1 — Additional file 1. [file 12944_2021_1516_MOESM1_ESM.docx]

**Supplementary table 1.** PRISMA checklist

| Section/topic | **#** | **Circulating retinol binding protein 4 levels in coronary artery disease: a systematic review and meta-analysis** | **Reported on page #** |
| --- | --- | --- | --- |
| **TITLE** | | |  |
| Title | 1 | Identify the report as a systematic review, meta-analysis, or both. | Page 1 |
| **ABSTRACT** | | |  |
| Structured summary | 2 | Provide a structured summary including, as applicable: background; objectives; data sources; study eligibility criteria, participants, and interventions; study appraisal and synthesis methods; results; limitations; conclusions and implications of key findings; systematic review registration number. | Page 3 |
| **INTRODUCTION** | | |  |
| Rationale | 3 | Describe the rationale for the review in the context of what is already known. | Page 5 and 6 |
| Objectives | 4 | Provide an explicit statement of questions being addressed with reference to participants, interventions, comparisons, outcomes, and study design (PICOS). | Page 5 and 6 |
| **METHODS** | | |  |
| Protocol and registration | 5 | Indicate if a review protocol exists, if and where it can be accessed (e.g., Web address), and, if available, provide registration information including registration number. | Page 6 |
| Eligibility criteria | 6 | Specify study characteristics (e.g., PICOS, length of follow-up) and report characteristics (e.g., years considered, language, publication status) used as criteria for eligibility, giving rationale. | Page 7 |
| Information sources | 7 | Describe all information sources (e.g., databases with dates of coverage, contact with study authors to identify additional studies) in the search and date last searched. | Page 7 |
| Search | 8 | Present full electronic search strategy for at least one database, including any limits used, such that it could be repeated. | Page 7 |
| Study selection | 9 | State the process for selecting studies (i.e., screening, eligibility, included in systematic review, and, if applicable, included in the meta-analysis). | Page 7 |
| Data collection process | 10 | Describe method of data extraction from reports (e.g., piloted forms, independently, in duplicate) and any processes for obtaining and confirming data from investigators. | Page 7 and 8 |
| Data items | 11 | List and define all variables for which data were sought (e.g., PICOS, funding sources) and any assumptions and simplifications made. | Page 7 |
| Risk of bias in individual studies | 12 | Describe methods used for assessing risk of bias of individual studies (including specification of whether this was done at the study or outcome level), and how this information is to be used in any data synthesis. | Page 8 |
| Summary measures | 13 | State the principal summary measures (e.g., risk ratio, difference in means). | Page 8 |
| Synthesis of results | 14 | Describe the methods of handling data and combining results of studies, if done, including measures of consistency (e.g., I^2^) for each meta-analysis. | Page 8 |

| Risk of bias across studies | 15 | Specify any assessment of risk of bias that may affect the cumulative evidence (e.g., publication bias, selective reporting within studies). | Page 9 |
| --- | --- | --- | --- |
| Additional analyses | 16 | Describe methods of additional analyses (e.g., sensitivity or subgroup analyses, meta-regression), if done, indicating which were pre-specified. | Page 9 |
| **RESULTS** | | |  |
| Study selection | 17 | Give numbers of studies screened, assessed for eligibility, and included in the review, with reasons for exclusions at each stage, ideally with a flow diagram. | Page 9 and 10  Figure 1 |
| Study characteristics | 18 | For each study, present characteristics for which data were extracted (e.g., study size, PICOS, follow-up period) and provide the citations. | Page 10  Table 1 |
| Risk of bias within studies | 19 | Present data on risk of bias of each study and, if available, any outcome level assessment (see item 12). | Page 10  Supplementary table 4 |
| Results of individual studies | 20 | For all outcomes considered (benefits or harms), present, for each study: (a) simple summary data for each intervention group (b) effect estimates and confidence intervals, ideally with a forest plot. | Figure 2 |
| Synthesis of results | 21 | Present results of each meta-analysis done, including confidence intervals and measures of consistency. | Page 11  Figure 2 |
| Risk of bias across studies | 22 | Present results of any assessment of risk of bias across studies (see Item 15). | Page 12  Figure 5 |
| Additional analysis | 23 | Give results of additional analyses, if done (e.g., sensitivity or subgroup analyses, meta-regression [see Item 16]). | Page 11-12  Figure 3 and 4 supplementary table 5 |
| **DISCUSSION** | | |  |
| Summary of evidence | 24 | Summarize the main findings including the strength of evidence for each main outcome; consider their relevance to key groups (e.g., healthcare providers, users, and policy makers). | Page 12 |
| Limitations | 25 | Discuss limitations at study and outcome level (e.g., risk of bias), and at review-level (e.g., incomplete retrieval of identified research, reporting bias). | Page 15-16 |
| Conclusions | 26 | Provide a general interpretation of the results in the context of other evidence, and implications for future research. | Page 16 |
| **FUNDING** | | |  |
| Funding | 27 | Describe sources of funding for the systematic review and other support (e.g., supply of data); role of funders for the systematic review. | Page 17 |

**Supplementary table 2.** Details of search strategy

| Dataset | No. | Query | Results |
| --- | --- | --- | --- |
| Pubmed | #1 | "retinol binding protein 4"[All Fields] OR "RBP4"[All Fields] OR plasma retinol binding proteins[MeSH Terms] | 2052 |
|  | #2 | "coronary artery disease"[All Fields] OR "CAD"[All Fields] OR "coronary heart disease"[All Fields] OR "CHD"[All Fields] OR coronary artery disease[MeSH] OR myocardial infarction[MeSH] OR "myocardial infarction"[All Fields] OR "ischemic heart disease"[All Fields] OR myocardial ischemia[MeSH] OR "myocardial ischemia"[All Fields] OR angina pectoris[All Fields] OR angina pectoris[MeSH] | 598123 |
|  | #3 | #1 AND #2 | 51 |
| Embase | #1 | 'retinol binding protein 4':ti,ab,kw OR rbp4:ti,ab,kw OR 'retinol binding protein 4'/exp | 2616 |
|  | #2 | 'coronary artery disease':ti,ab,kw OR 'coronary heart disease':ti,ab,kw OR cad:ti,ab,kw OR chd:ti,ab,kw OR 'myocardial infarction':ti,ab,kw OR 'ischemic heart disease':ti,ab,kw OR 'myocardial ischemia':ti,ab,kw OR 'angina pectoris':ti,ab,kw OR 'coronary artery disease'/exp OR 'heart infarction'/exp OR 'ischemic heart disease'/exp OR 'heart muscle ischemia'/exp OR 'angina pectoris'/exp | 978923 |
|  | #3 | #1 AND #2 | 122 |
| Web of science | #1 | (TS=(retinol binding protein 4)) OR TS=(RBP4) | 3816 |
|  | #2 | ((((((TS=(coronary artery disease)) OR TS=(CAD)) OR TS=(CHD)) OR TS=(myocardial infarction)) OR TS=(ischemic heart disease)) OR TS=(myocardial ischemia)) OR TS=(angina pectoris) | 581257 |
|  | #3 | #1 AND #2 | 119 |
| Google scholar | #1 | (“retinol binding protein 4” OR “RBP4”) AND (“coronary artery disease” OR “CAD” OR “CHD” OR “myocardial infarction” OR “ischemic heart disease” OR “myocardial ischemia” OR “angina pectoris”) | 738 |
| ClinicalTrials.gov | #1 | (retinol binding protein 4 OR RBP4) AND (coronary artery disease OR CAD OR CHD OR myocardial infarction OR ischemic heart disease OR myocardial ischemia OR angina pectoris) | 3 |

**Supplementary table 3.** List of excluded studies and reason for exclusion.

| **Exclusion reasons** | **Reference number** |
| --- | --- |
| **Duplicates** | [1-123] |
| **Title and abstract screen** |  |
| Animal studies | [9, 68, 124-169] |
| Basic or cell experiments | [45, 170-206] |
| Lack of information or data | [207-232] |
| Disease isn’t CAD | [37, 38, 54, 72, 92, 101, 111, 233-277] |
| Not investigating RBP4 or CAD | [1, 24, 31, 35, 36, 51, 59, 93, 113, 115, 116, 233, 241, 278-459] |
| Review, meta-analysis, conference abstract, letter or comments | [2, 10, 12, 14, 15, 18, 20, 22, 25-27, 33, 40, 47, 61, 70, 77, 87-90, 94, 96, 112, 123, 460-754] |
| Exposure isn’t RBP4 | [41, 62, 755-790] |
| Patients with interventions | [108, 791-815] |
| Language isn’t English | [57, 816-924] |
| **Full-text screen** |  |
| Didn’t have a control group | [925, 926] |
| Didn’t measure RBP4 | [85, 785, 927-930] |
| Outcome isn’t CAD | [931-944] |
| Cases less than ten | [945] |

1. Adams-Huet B, Jialal I: The neutrophil count is superior to the neutrophil/lymphocyte ratio as a biomarker of inflammation in nascent metabolic syndrome. Annals of Clinical Biochemistry. 2019;56:715-716.

2. Agasthi P, Aloor S, Chenna A, Harris R: Association between serum retinol binding protein-4 level and coronary artery disease: A meta analysis. Arteriosclerosis, Thrombosis, and Vascular Biology. 2015;35.

3. Agasthi P, Aloor S, Chenna A, Harris R: Association Between Serum Retinol Binding Protein-4 Level and Coronary Artery Disease: A Meta Analysis. Arteriosclerosis, Thrombosis, and Vascular Biology. 2015;35:A344-A344.

4. Agra RM, Fernández-Trasancos Á, Sierra J, González-Juanatey JR, Eiras S: Differential Association of S100A9, an Inflammatory Marker, and p53, a Cell Cycle Marker, Expression with Epicardial Adipocyte Size in Patients with Cardiovascular Disease. Inflammation. 2014;37:1504-1512.

5. Al-Daghri N, Al-Attas O, Alokail M, Draz H, Bamakhramah A, Sabico S: Retinol binding protein-4 is associated with TNF-α and not insulin resistance in subjects with type 2 diabetes mellitus and coronary heart disease. Endocrine Abstracts. 2010;20:P384.

6. Al-Daghri NM, Al-Attas OS, Alokail M, Draz HM, Bamakhramah A, Sabico S: Retinol binding protein-4 is associated with TNF-alpha and not insulin resistance in subjects with type 2 diabetes mellitus and coronary heart disease. Dis Markers. 2009;26:135-140.

7. Al-Mass A: Biochemical basis of pancreatic islet ß-cell adaptation and failure in high fat fed diet-induced obese mice. 2017.

8. Alkharfy KM, Al-Daghri NM, Vanhoutte PM, Krishnaswamy S, Xu A: Serum Retinol-Binding Protein 4 as a Marker for Cardiovascular Disease in Women. Plos One. 2012;7.

9. Amengual J, Coronel J, Marques C, Aradillas-García C, Morales JMV, Andrade FCD, Erdman JW, Teran-Garcia M: β-Carotene oxygenase 1 activity modulates circulating cholesterol concentrations in mice and humans. Journal of Nutrition. 2020;150:2023-2030.

10. Andersson C, Johnson AD, Benjamin EJ, Levy D, Vasan RS: 70-year legacy of the Framingham Heart Study. Nature Reviews Cardiology. 2019;16:687-698.

11. Azo Najeeb H, Ahmad Qasim B, Ahmad Mohammed A: Parental history of coronary artery disease among adults with hypothyroidism: Case controlled study. Annals of Medicine and Surgery. 2020;60:92-101.

12. Balta I, Balta S, Demirkol S, Celik T: Other inflammatory markers and related factors should be kept in mind in metabolic syndrome with psoriasis patients. Archives of Dermatological Research. 2013;305:459-460.

13. Beetham HGS: **Mechanisms of Hereditary Diffuse Gastric Cancer Initiation: The role of E-cadherin in the Epithelial-Mesenchymal Transition.** University of Otago, 2012.

14. Blüher M: Are there still healthy obese patients? Current Opinion in Endocrinology, Diabetes and Obesity. 2012;19:341-346.

15. Bodhini D, Mohan V: Mediators of insulin resistance & cardiometabolic risk: Newer insights. Indian Journal of Medical Research. 2018;148:127-129.

16. Brown CE, McCarthy NS, Hughes AD, Sever P, Stalmach A, Mullen W, Dominiczak AF, Sattar N, Mischak H, Thom S, et al: Urinary proteomic biomarkers to predict cardiovascular events. Proteomics - Clinical Applications. 2015;9:610-617.

17. Buckner T, Shao B, Eckel RH, Heinecke JW, Bornfeldt KE, Snell-Bergeon J: Association of apolipoprotein C3 with insulin resistance and coronary artery calcium in patients with type 1 diabetes. Journal of Clinical Lipidology. 2021;15:235-242.

18. Cacciapuoti F: Visceral adiposity as a cause of some cardiovascular disorders. Old and new adipocytokines. Obesity and Metabolism. 2010;6:39-45.

19. Cacciapuoti F: Visceral adiposity as a cause of some cardiovascular disorders. Old and new adipocytokines. Obesity and Metabolism-Milan. 2010;6:39-45.

20. Caiazzo G, Fabbrocini G, Di Caprio R, Raimondo A, Scala E, Balato N, Balato A: Psoriasis, cardiovascular events, and biologics: Lights and shadows. Frontiers in Immunology. 2018;9.

21. Camera A, Hopps E, Caimi G: Metabolic syndrome: From insulin resistance to adipose tissue dysfunction. Minerva Medica. 2008;99:307-321.

22. Carpino PA, Goodwin B: Diabetes area participation analysis: A review of companies and targets described in the 2008 2010 patent literature. Expert Opinion on Therapeutic Patents. 2010;20:1627-1651.

23. Cheng X, Wu Z, Yuan B: Original article diagnostic and predictive value of serum ldl/hdl and rbp4 levels in restenosis after revascularization in patients with coronary heart disease (Chd). International Journal of Clinical and Experimental Medicine. 2019;12:10783-10788.

24. Cheow ESH, Cheng WC, Yap T, Dutta B, Lee CN, Kleijn DPVD, Sorokin V, Sze SK: Myocardial Injury Is Distinguished from Stable Angina by a Set of Candidate Plasma Biomarkers Identified Using iTRAQ/MRM-Based Approach. Journal of Proteome Research. 2018;17:499-515.

25. Choi SH, Hong ES, Lim S: Clinical implications of adipocytokines and newly emerging metabolic factors with relation to insulin resistance and cardiovascular health. Frontiers in Endocrinology. 2013;4.

26. Christou GA, Tselepis AD, Kiortsis DN: The metabolic role of retinol binding protein 4: An update. Hormone and Metabolic Research. 2012;44:6-14.

27. Coronel J, Pinos I, Amengual J: β-carotene in obesity research: Technical considerations and current status of the field. Nutrients. 2019;11.

28. Cubedo J, Padró T, Cinca J, Mata P, Alonso R, Badimon L: Retinol-binding protein 4 levels and susceptibility to ischaemic events in men. European Journal of Clinical Investigation. 2014;44:266-275.

29. Cubedo J, Padro T, Cinca J, Mata P, Alonso R, Badimon L: Retinol-binding protein 4 levels and susceptibility to ischaemic events in men. European Journal of Clinical Investigation. 2014;44:266-275.

30. Cubedo J, Padró T, Formiga F, Ferrer A, Padrós G, Peña E, Badimon L: Inflammation and hemostasis in older octogenarians: implication in 5-year survival. Translational Research. 2017;185:34-46.e39.

31. De Lillo A, De Angelis F, Di Girolamo M, Luigetti M, Frusconi S, Manfellotto D, Fuciarelli M, Polimanti R: Phenome-wide association study of TTR and RBP4 genes in 361,194 individuals reveals novel insights in the genetics of hereditary and wildtype transthyretin amyloidoses. Human Genetics. 2019;138:1331-1340.

32. Dong H, Li X, Tang Y: Serum retinol-binding protein-4 level is a high risk factor for coronary heart disease in Chinese. Clinical Laboratory. 2015;61:1675-1678.

33. Duntas LH, Biondi B: New insights into subclinical hypothyroidism and cardiovascular risk. Seminars in Thrombosis and Hemostasis. 2011;37:27-34.

34. Elmadhun NY, Lassaletta AD, Chu LM, Sellke FW: Metformin alters the insulin signaling pathway in ischemic cardiac tissue in a swine model of metabolic syndrome. Journal of Thoracic and Cardiovascular Surgery. 2013;145:258-266.

35. Engin A: **Does bariatric surgery improve obesity associated comorbid conditions.** vol. 960. pp. 545-570; 2017:545-570.

36. Espe KM, Raila J, Henze A, Krane V, Schweigert FJ, Hocher B, Wanner C, Drechsler C: Impact of vitamin A on clinical outcomes in haemodialysis patients. Nephrology Dialysis Transplantation. 2011;26:4054-4061.

37. Eyzaguirre F, Mericq V: Insulin resistance markers in children. Hormone Research. 2009;71:65-74.

38. Fabregate R, Sanchez O, Marin E, Reyes A, Martinez C, Tello S, Fabregate M, Rodriguez A, Saban-Ruiz J: Myeloperoxidase, leptin and retinol binding protein-4 as biomarkers of osteopenia and atherosclerosis, two sister-diseases. Journal of Clinical Hypertension. 2011;13:A51.

39. Gandhi H, Upaganlawar A, Balaraman R: Adipocytokines: The pied pipers. Journal of Pharmacology and Pharmacotherapeutics. 2010;1:9-17.

40. Garić D, Dumut DC, Shah J, De Sanctis JB, Radzioch D: The role of essential fatty acids in cystic fibrosis and normalizing effect of fenretinide. Cellular and Molecular Life Sciences. 2020;77:4255-4267.

41. Gehrau R, Maluf D, Cathro H, King A, Suh J, Brayman K, Ladie D, Mas V: Portrait of CAD with IF-TA development post kidney transplantation. Proteomics as noninvasive biomarkers. American Journal of Transplantation. 2013;13:83.

42. Gerdes S, Osadtschy S, Rostami-Yazdi M, Buhles N, Weichenthal M, Mrowietz U: Leptin, adiponectin, visfatin and retinol-binding protein-4 - mediators of comorbidities in patients with psoriasis? Experimental Dermatology. 2012;21:43-47.

43. Gillis J, Pavlidis P: Gene Ontology matrices (with descriptions, IDs, etc) from" Guilt by Association" Is the Exception Rather Than the Rule in Gene Networks. Gillis, J. and Pavlidis, P.(2012) PLoS Computational Biology, 8 (3). 2012.

44. Guan WJ, Yang GJ: Significance of change of retinol binding protein 4 level of plasma of patients with coronary heart disease complicated with hyperlipidemia. European Review for Medical and Pharmacological Sciences. 2016;20:4136-4140.

45. Guantario B, Conigliaro A, Amicone L, Sambuy Y, Bellovino D: The new murine hepatic 3A cell line responds to stress stimuli by activating an efficient Unfolded Protein Response (UPR). Toxicology in Vitro. 2012;26:7-15.

46. Guglielmi V, Morretti T, Morazzini M, Sbraccia P: Fat and Lipid Partitioning: Phenotyping Beyond BMI. J Diabetes Endocrinol Metab Disord 2017: 1-6. DOI: <https://doi> org/1029199/DEMD. 2017;101011:3.

47. Gurav AN: Periodontitis and insulin resistance: Casual or causal relationship? Diabetes and Metabolism Journal. 2012;36:404-411.

48. Harwood Jr HJ: The adipocyte as an endocrine organ in the regulation of metabolic homeostasis. Neuropharmacology. 2012;63:57-75.

49. Heitmeier MR, Payne MA, Weinheimer C, Kovacs A, Hresko RC, Jay PY, Hruz PW: Metabolic and Cardiac Adaptation to Chronic Pharmacologic Blockade of Facilitative Glucose Transport in Murine Dilated Cardiomyopathy and Myocardial Ischemia. Scientific reports. 2018;8:6475.

50. Hepler C, Shao B, Zhang Q, Henry G, Shao M, Vishvanath L, Ghaben A, B Mobley A, Hon G, Gupta R: eLife-Identification of functionally distinct fibro-inflammatory and adipogenic stromal subpopulations in visceral adipose tissue of adult mice. pdf. 2019.

51. Hong SJ, Choi SC, Ahn CM, Park JH, Kim JS, Lim D-S: Telmisartan reduces neointima volume and pulse wave velocity 8 months after zotarolimus-eluting stent implantation in hypertensive type 2 diabetic patients. Heart. 2011;97:1425-1432.

52. Hong SJ, Choi SC, Ahn CM, Park JH, Kim JS, Lim DS: Telmisartan reduces neointima volume and pulse wave velocity 8 months after zotarolimus-eluting stent implantation in hypertensive type 2 diabetic patients. Heart. 2011;97:1425-1432.

53. Hong SJ, Choi SC, Cho JY, Joo HJ, Park JH, Yu CW, Lim DS: Pioglitazone increases circulating microRNA-24 with decrease in coronary neointimal hyperplasia in type 2 diabetic patients: Optical coherence tomography analysis. Circulation Journal. 2015;79:880-888.

54. Huang G, Wang D, Khan UI, Zeb I, Manson JE, Miller V, Hodis HN, Budoff MJ, Merriam GR, Harman MS, et al: Associations between retinol-binding protein 4 and cardiometabolic risk factors and subclinical atherosclerosis in recently postmenopausal women: cross-sectional analyses from the KEEPS study. Cardiovasc Diabetol. 2012;11:52.

55. Huang G, Wang D, Khan UI, Zeb I, Manson JE, Miller V, Hodis HN, Budoff MJ, Merriam GR, Harman MS, et al: Associations between retinol-binding protein 4 and cardiometabolic risk factors and subclinical atherosclerosis in recently postmenopausal women: Cross-sectional analyses from the KEEPS study. Cardiovascular Diabetology. 2012;11.

56. Ingelsson E, Sundström J, Melhus H, Michaëlsson K, Berne C, Vasan RS, Risérus U, Blomhoff R, Lind L, Ärnlöv J: Circulating retinol-binding protein 4, cardiovascular risk factors and prevalent cardiovascular disease in elderly. Atherosclerosis. 2009;206:239-244.

57. Jing L, Xiaoming H, Youzhen J, Fuxiu L, Zhongwen M: Expression and clinical significance of serum retinol binding protein 4, superoxide dismutase and hypersensitive C-reactive protein in patients with acute ST-segment elevated myocardial infarction. Zhonghua Wei Zhong Bing Ji Jiu Yi Xue. 2020;32:1199-1202.

58. Kadoglou NPE, Lambadiari V, Gastounioti A, Gkekas C, Giannakopoulos TG, Koulia K, Maratou E, Alepaki M, Kakisis J, Karakitsos P, et al: The relationship of novel adipokines, RBP4 and omentin-1, with carotid atherosclerosis severity and vulnerability. Atherosclerosis. 2014;235:606-612.

59. Kałuzna S, Nawrocki MJ, Jopek K, Hutchings G, Perek B, Jemielity M, Kempisty B, Malińska A, Mozdziak P, Nowicki M: In search of markers useful for evaluation of graft patency-molecular analysis of 'muscle system process' for internal thoracic artery and saphenous vein conduits. Medical Journal of Cell Biology. 2020;8:12-23.

60. Kraus BJ, Sartoretto JL, Polak P, Hosooka T, Shiroto T, Eskurza I, Lee SA, Jiang H, Michel T, Kahn BB: Novel role for retinol-binding protein 4 in the regulation of blood pressure. Faseb j. 2015;29:3133-3140.

61. Laakso M: Cardiovascular disease in type 2 diabetes from population to man to mechanisms: The Kelly West award lecture 2008. Diabetes Care. 2010;33:442-449.

62. Laborde CM, Alonso-Orgaz S, Mourino-Alvarez L, Moreu J, Vivanco F, Padial LR, Barderas MG: The plasma proteomic signature as a strategic tool for early diagnosis of acute coronary syndrome. Proteome Science. 2014;12.

63. Lambadiari V, Kadoglou NP, Stasinos V, Maratou E, Antoniadis A, Kolokathis F, Parissis J, Hatziagelaki E, Iliodromitis EK, Dimitriadis G: Serum levels of retinol-binding protein-4 are associated with the presence and severity of coronary artery disease. Cardiovascular diabetology. 2014;13:1-8.

64. Lambadiari V, Kadoglou NPE, Stasinos V, Maratou E, Antoniadis A, Kolokathis F, Parissis J, Hatziagelaki E, Iliodromitis EK, Dimitriadis G: Serum levels of retinol-binding protein-4 are associated with the presence and severity of coronary artery disease. Cardiovascular Diabetology. 2014;13.

65. Li F: Retinol-binding protein 4 as a novel cardiovascular disease risk factor in patients with coronary artery disease and hyperinsulinemia. Heart. 2013;99:A158.

66. Li F, Xia K, Li C, Yang T: Retinol-binding protein 4 as a novel risk factor for cardiovascular disease in patients with coronary artery disease and hyperinsulinemia. The American journal of the medical sciences. 2014;348:474-479.

67. Li F, Xia K, Li C, Yang T: Retinol-binding protein 4 as a novel risk factor for cardiovascular disease in patients with coronary artery disease and hyperinsulinemia. American Journal of the Medical Sciences. 2014;348:474-479.

68. Li F, Xia K, Sheikh MSA, Cheng J, Li C, Yang T: Involvement of RBP4 in hyperinsulinism-induced vascular smooth muscle cell proliferation. Endocrine. 2015;48:472-482.

69. Li F, Yang T, Zhao Z, Xia K: Plasma level of RBP4 in patients with coronary heart disease and the effect of hyperinsulinemia. Zhong nan da xue xue bao Yi xue ban= Journal of Central South University Medical Sciences. 2012;37:1177-1182.

70. Li M, Zhang EY, Zhang X, Li GP: Mechanism by which statins influence insulin signaling pathway. Chinese Medical Journal. 2014;127:3664-3668.

71. Lim S, Hivert MF: Update on the Role of Adipokines in Atherosclerosis and Cardiovascular Diseases. Current Cardiovascular Risk Reports. 2012;6:53-61.

72. Liu G, Ding M, Chiuve SE, Rimm EB, Franks PW, Meigs JB, Hu FB, Sun Q: Plasma Levels of Fatty Acid-Binding Protein 4, Retinol-Binding Protein 4, High-Molecular-Weight Adiponectin, and Cardiovascular Mortality among Men with Type 2 Diabetes: A 22-Year Prospective Study. Arteriosclerosis, Thrombosis, and Vascular Biology. 2016;36:2259-2267.

73. Liu T, Han C, Sun L, Ding Z, Shi F, Wang R, Wang W, Shan W, Zhang Y, Hu N, et al: Association between new circulating proinflammatory and anti-inflammatory adipocytokines with coronary artery disease. Coron Artery Dis. 2019;30:528-535.

74. Liu T, Han C, Sun L, Ding Z, Shi F, Wang R, Wang W, Shan W, Zhang Y, Hu N, et al: Association between new circulating proinflammatory and anti-inflammatory adipocytokines with coronary artery disease. Coronary Artery Disease. 2019;30:528-535.

75. Liu Y, Wang D, Chen H, Xia M: Circulating retinol binding protein 4 is associated with coronary lesion severity of patients with coronary artery disease. Atherosclerosis. 2015;238:45-51.

76. Llombart V, García‐Berrocoso T, Bustamante A, Giralt D, Rodriguez‐Luna D, Muchada M, Penalba A, Boada C, Hernández‐Guillamon M, Montaner J: Plasmatic retinol‐binding protein 4 and glial fibrillary acidic protein as biomarkers to differentiate ischemic stroke and intracerebral hemorrhage. Journal of neurochemistry. 2016;136:416-424.

77. Lorenzet R, Napoleone E, Cutrone A, Donati MB: Thrombosis and obesity: Cellular bases. Thrombosis Research. 2012;129:285-289.

78. Mahmoudi MJ, Mahmoudi M, Siassi F, Hedayat M, Pasalar P, Chamari M, Abolhassani H, Rezaei N, Saboor-Yaraghi A-A: Circulating retinol-binding protein 4 concentrations in patients with coronary artery disease and patients with type 2 diabetes mellitus. International Journal of Diabetes in Developing Countries. 2012;32:105-110.

79. Majerczyk M, Choręza P, Bożentowicz-Wikarek M, Brzozowska A, Arabzada H, Owczarek A, Mossakowska M, Grodzicki T, Zdrojewski T, Więcek A, et al: Increased plasma RBP4 concentration in older hypertensives is related to the decreased kidney function and the number of antihypertensive drugs-results from the PolSenior substudy. J Am Soc Hypertens. 2017;11:71-80.

80. Majerczyk M, Choręza P, Bożentowicz-Wikarek M, Brzozowska A, Arabzada H, Owczarek A, Mossakowska M, Grodzicki T, Zdrojewski T, Więcek A, et al: Increased plasma RBP4 concentration in older hypertensives is related to the decreased kidney function and the number of antihypertensive drugs—results from the PolSenior substudy. Journal of the American Society of Hypertension. 2017;11:71-80.

81. Majerczyk M, Choręza P, Mizia-Stec K, Bożentowicz-Wikarek M, Brzozowska A, Arabzada H, Owczarek AJ, Szybalska A, Grodzicki T, Więcek A, et al: Plasma Level of Retinol-Binding Protein 4, N-Terminal proBNP and Renal Function in Older Patients Hospitalized for Heart Failure. Cardiorenal Med. 2018;8:237-248.

82. Majerczyk M, Chorȩza P, Mizia-Stec K, Bozentowicz-Wikarek M, Brzozowska A, Arabzada H, Owczarek AJ, Szybalska A, Grodzicki T, Wiȩcek A, et al: Plasma level of retinol-binding protein 4, n-terminal probnp and renal function in older patients hospitalized for heart failure. CardioRenal Medicine. 2018;8:237-248.

83. Mallat Z, Simon T, Benessiano J, Clement K, Taleb S, Wareham NJ, Luben R, Khaw K-T, Tedgui A, Boekholdt SM: Retinol-Binding Protein 4 and Prediction of Incident Coronary Events in Healthy Men and Women. Journal of Clinical Endocrinology & Metabolism. 2009;94:255-260.

84. Mallat Z, Simon T, Benessiano J, Clément K, Taleb S, Wareham NJ, Luben R, Khaw KT, Tedgui A, Boekholdt SM: Retinol-binding protein 4 and prediction of incident coronary events in healthy men and women. J Clin Endocrinol Metab. 2009;94:255-260.

85. Manfredi M, Chiariello C, Conte E, Castagna A, Robotti E, Gosetti F, Patrone M, Martinelli N, Bassi A, Cecconi D, et al: Plasma Proteome Profiles of Stable CAD Patients Stratified According to Total Apo C-III Levels. Proteomics Clin Appl. 2019;13:e1800023.

86. Manfredi M, Chiariello C, Conte E, Castagna A, Robotti E, Gosetti F, Patrone M, Martinelli N, Bassi A, Cecconi D, et al: Plasma Proteome Profiles of Stable CAD Patients Stratified According to Total Apo C-III Levels. Proteomics - Clinical Applications. 2019;13.

87. Martini LA, Catania AS, Ferreira SRG: Role of vitamins and minerals in prevention and management of type 2 diabetes mellitus. Nutrition Reviews. 2010;68:341-354.

88. Matsuda M, Shimomura I: Recent research on adipocytes as endocrine cells. Japanese Journal of Clinical Chemistry. 2008;37:238-244.

89. McIntyre HD, Thomae MK, Wong SF, Idris N, Callaway LK: Pregnancy in type 2 diabetes mellitus - Problems & promises. Current Diabetes Reviews. 2009;5:190-200.

90. McKenney RL, Short DK: Tipping the balance: The pathophysiology of obesity and type 2 diabetes mellitus. Surgical Clinics of North America. 2011;91:1139-1148.

91. Miller AP, Coronel J, Amengual J: The role of β-carotene and vitamin A in atherogenesis: Evidences from preclinical and clinical studies. Biochimica et Biophysica Acta - Molecular and Cell Biology of Lipids. 2020;1865.

92. Murata M, Saito T, Otani T, Sasaki M, Ikoma A, Toyoshima H, Kawakami M, Ishikawa SE: An increase in serum retinol-binding protein 4 in the type 2 diabetic subjects with nephropathy. Endocrine Journal. 2009;56:287-294.

93. Najeeb HA, Al-Timimi DJ, Qasim BA, Mohammed AA: Parental history of coronary artery disease among adults with hypothyroidism: Case controlled study. Annals of Medicine and Surgery. 2020;60:92-101.

94. Oda E, Watanabe K: Letter by Oda et al regarding article, "Hypoadiponectinemia is associated with impaired glucose tolerance and coronary artery disease in non-diabetic men". Circulation Journal. 2008;72:505.

95. Oda E, Watanabe K: Letter by Oda et al regarding article, "Hypoadiponectinemia is Associated With Impaired Glucose Tolerance and Coronary Artery Disease In Non-Diabetic Men". Circulation Journal. 2008;72:505-505.

96. Opatrilova R, Caprnda M, Kubatka P, Valentova V, Uramova S, Nosal V, Gaspar L, Zachar L, Mozos I, Petrovic D, et al: Adipokines in neurovascular diseases. Biomedicine and Pharmacotherapy. 2018;98:424-432.

97. Padró T, Cubedo J, Camino S, Béjar MT, Ben-Aicha S, Mendieta G, Escolà-Gil JC, Escate R, Gutiérrez M, Casani L: Detrimental effect of hypercholesterolemia on high-density lipoprotein particle remodeling in pigs. Journal of the American College of Cardiology. 2017;70:165-178.

98. Padró T, Cubedo J, Camino S, Béjar MT, Ben-Aicha S, Mendieta G, Escolà-Gil JC, Escate R, Gutiérrez M, Casani L, et al: Detrimental Effect of Hypercholesterolemia on High-Density Lipoprotein Particle Remodeling in Pigs. J Am Coll Cardiol. 2017;70:165-178.

99. Pan Y, Wang L, Xie Y, Tan Y, Chang C, Qiu X, Li X: Characterization of differentially expressed plasma proteins in patients with acute myocardial infarction. Journal of Proteomics. 2020;227.

100. Pan Y, Wang L, Xie Y, Tan Y, Chang C, Qiu X, Li X: Characterization of differentially expressed plasma proteins in patients with acute myocardial infarction. Journal of Proteomics. 2020;227:103923.

101. Patterson CC, Blankenberg S, Ben-Shlomo Y, Heslop L, Bayer A, Lowe G, Zeller T, Gallacher J, Young I, Yarnell J: Which biomarkers are predictive specifically for cardiovascular or for non-cardiovascular mortality in men? Evidence from the Caerphilly Prospective Study (CaPS). International Journal of Cardiology. 2015;201:113-118.

102. Promintzer M, Krebs M, Todoric J, Luger A, Bischof MG, Nowotny P, Wagner O, Esterbauer H, Anderwald C: Insulin resistance is unrelated to circulating retinol binding protein and protein C inhibitor. Journal of Clinical Endocrinology & Metabolism. 2007;92:4306-4312.

103. Puig N, Creus A, Miñambres I, Gil P, Perez A, Sanchez-Quesada JL, Benitez S: Epicardial adipose tissue from type 2 diabetic patients displays features ascribed to metabolic alterations and inflammation. Atherosclerosis. 2020;315:e70.

104. Qin B, Polansky MM, Harry D, Anderson RA: Green tea polyphenols improve cardiac muscle mrna and protein levels of signal pathways related to insulin and lipid metabolism and inflammation in insulin-resistant rats. Molecular Nutrition and Food Research. 2010;54:S14-S23.

105. Rahim MAA, Rahim ZHA, Ahmad WAW, Bakri MM, Ismail MD, Hashim OH: Inverse changes in plasma tetranectin and titin levels in patients with type 2 diabetes mellitus: A potential predictor of acute myocardial infarction? article. Acta Pharmacologica Sinica. 2018;39:1197-1207.

106. Rist PM, Jiménez MC, Tworoger SS, Hu FB, Manson JE, Sun Q, Rexrode KM: Plasma Retinol-Binding Protein 4 Levels and the Risk of Ischemic Stroke among Women. Journal of Stroke and Cerebrovascular Diseases. 2018;27:68-75.

107. Robich MP, Osipov RM, Chu LM, Han Y, Feng J, Nezafat R, Clements RT, Manning WJ, Sellke FW: Resveratrol modifies risk factors for coronary artery disease in swine with metabolic syndrome and myocardial ischemia. European Journal of Pharmacology. 2011;664:45-53.

108. Rodrigues B, Lira FS, Consolim-Colombo FM, Rocha JA, Caperuto EC, De Angelis K, Irigoyen MC: Role of exercise training on autonomic changes and inflammatory profile induced by myocardial infarction. Mediators of Inflammation. 2014;2014.

109. Salgado-Somoza A, Teijeira-Fernández E, Rubio J, Couso E, González-Juanatey JR, Eiras S: Coronary artery disease is associated with higher epicardial Retinol-binding protein 4 (RBP4) and lower glucose transporter (GLUT) 4 levels in epicardial and subcutaneous adipose tissue. Clinical Endocrinology. 2012;76:51-58.

110. Salgado-Somoza A, Teijeira-Fernandez E, Rubio J, Couso E, Gonzalez-Juanatey JR, Eiras S: Coronary artery disease is associated with higher epicardial Retinol-binding protein 4 (RBP4) and lower glucose transporter (GLUT) 4 levels in epicardial and subcutaneous adipose tissue. Clinical Endocrinology. 2012;76:51-58.

111. Sasaki M, Ishikawa S, Kawakami M: Increased plasma retinol-binding protein 4 and reduced adiponectin in the patients with cerebral infarction. Diabetes. 2009;58.

112. Sun L, Zong G, Li H, Lin X: Fatty acids and cardiometabolic health: a review of studies in Chinese populations. European Journal of Clinical Nutrition. 2021;75:253-266.

113. Syed Ikmal Hj S, Zaman Huri H, Vethakkan V, Wan Ahmad W: Potential biomarkers for insulin resistance and atherosclerosis among type 2 diabetes mellitus patients with coronary artery disease. Obesity Reviews. 2014;15:72.

114. Tang D, Wu Q, Yuan Z, Xu J, Zhang H, Jin Z, Zhang Q, Xu M, Wang Z, Dai Z, et al: Identification of key pathways and genes changes in pancreatic cancer cells (BXPC-3) after cross-talk with primary pancreatic stellate cells using bioinformatics analysis. Neoplasma. 2019;66:681-693.

115. Taylan A, Toprak B, Akinci B, Birlik M, Arslan FD, Gundogdu B, Colak A: Disease activity correlates with insulin resistance and adipocytokines in patients with DMARD-naïve rheumatoid arthritis. Annals of the Rheumatic Diseases. 2019;78:642-643.

116. Toussirot É, Aubin F, Dumoulin G: Relationships between adipose tissue and psoriasis, with or without arthritis. Frontiers in Immunology. 2014;5.

117. Verhagen SN, Buijsrogge MP, Vink A, Van Herwerden LA, Van Der Graaf Y, Visseren FLJ: Secretion of adipocytokines by perivascular adipose tissue near stenotic and non-stenotic coronary artery segments in patients undergoing CABG. Atherosclerosis. 2014;233:242-247.

118. von Eynatten M, Lepper PM, Liu D, Lang K, Baumann M, Nawroth PP, Bierhaus A, Dugi KA, Heemann U, Allolio B, Humpert PM: Retinol-binding protein 4 is associated with components of the metabolic syndrome, but not with insulin resistance, in men with type 2 diabetes or coronary artery disease. Diabetologia. 2007;50:1930-1937.

119. Xiaowen H, Guangxiao L, Qiongrui Z, Xin C, Chong W, Jingpu S: Association between retinol-binding protein 4 and coronary artery disease in Chinese: A Meta-analysis. Chinese Journal of Endemiology. 2015;36:1010-1014.

120. Xie XF, Chu HJ, Xu YF, Hua L, Wang ZP, Huang P, Jia HL, Zhang L: Proteomics study of serum exosomes in Kawasaki disease patients with coronary artery aneurysms. Cardiol J. 2019;26:584-593.

121. Yim J, Rabkin SW: Differences in Gene Expression and Gene Associations in Epicardial Fat Compared to Subcutaneous Fat. Hormone and Metabolic Research. 2017;49:327-337.

122. Yu F, Zhou X, Li Z, Feng X, Liao D, Liu Z, Huang Q, Li X, Yang Q, Xiao B, Xia J: Diagnostic Significance of Plasma Levels of Novel Adipokines in Patients With Symptomatic Intra- and Extracranial Atherosclerotic Stenosis. Frontiers in Neurology. 2019;10.

123. Zabetian-Targhi F, Mahmoudi MJ, Rezaei N, Mahmoudi M: Retinol Binding Protein 4 in Relation to Diet, Inflammation, Immunity, and Cardiovascular Diseases. Advances in Nutrition. 2015;6:748-762.

124. Al-Mass A: **Biochemical Basis of Pancreatic Islet β-Cell Adaptation and Failure in High Fat Fed Diet-Induced Obese Mice.** McGill University (Canada), 2017.

125. Alexander LEC, Drummond CA, Hepokoski M, Mathew D, Moshensky A, Willeford A, Das S, Singh P, Yong Z, Lee JH: Translational Physiology: Chronic inhalation of e-cigarette vapor containing nicotine disrupts airway barrier function and induces systemic inflammation and multiorgan fibrosis in mice. American Journal of Physiology-Regulatory, Integrative and Comparative Physiology. 2018;314:R834.

126. Bilbija D, Haugen F, Sagave J, Baysa A, Bastani N, Levy FO, Sirsjo A, Blomhoff R, Valen G: Retinoic Acid Signalling Is Activated in the Postischemic Heart and May Influence Remodelling. Plos One. 2012;7.

127. Bolduc C, Yoshioka M, St Amand J: Transcriptomic characterization of the long‐term dihydrotestosterone effects in adipose tissue. Obesity. 2007;15:1107-1132.

128. Bouchal P, Jarkovsky J, Hrazdilova K, Dvorakova M, Struharova I, Hernychova L, Damborsky J, Sova P, Vojtesek B: The new platinum-based anticancer agent LA-12 induces retinol binding protein 4 in vivo. Proteome science. 2011;9:1-9.

129. Chainani M, Sampsell B, Elliott RW: Localization of the gene for plasma retinol binding protein to the distal half of mouse chromosome 19. Genomics. 1991;9:376-379.

130. Choi JW, Joo JI, Kim DH, Wang X, Oh TS, Choi DK, Yun JW: Proteome changes in rat plasma in response to sibutramine. Proteomics. 2011;11:1300-1312.

131. Chuang C-M, Chang C-H, Wang H-E, Chen K-C, Peng C-C, Hsieh C-L, Peng RY: Valproic acid downregulates RBP4 and elicits hypervitaminosis A-teratogenesis—a kinetic analysis on retinol/retinoic acid homeostatic system. 2012.

132. Ding Y, Ding C, Wu X, Wu C, Qian L, Li D, Zhang W, Wang Y, Yang M, Wang L: Porcine LIF gene polymorphisms and their association with litter size traits in four pig breeds. Canadian Journal of Animal Science. 2020;100:85-92.

133. Ghotbi Ravandi E: Recruitment of Polycomb-Group Proteins at giant in Drosophila Embryos. 2019.

134. Heitmeier MR, Payne MA, Weinheimer C, Kovacs A, Hresko RC, Jay PY, Hruz PW: Metabolic and Cardiac Adaptation to Chronic Pharmacologic Blockade of Facilitative Glucose Transport in Murine Dilated Cardiomyopathy and Myocardial Ischemia. Sci Rep. 2018;8:6475.

135. Hepler C, Shan B, Zhang Q, Henry GH, Shao M, Vishvanath L, Ghaben AL, Mobley AB, Strand D, Hon GC: Identification of functionally distinct fibro-inflammatory and adipogenic stromal subpopulations in visceral adipose tissue of adult mice. Elife. 2018;7:e39636.

136. Inohara N, Koseki T, Chen S, Benedict MA, Núñez G: Identification of regulatory and catalytic domains in the apoptosis nuclease DFF40/CAD. J Biol Chem. 1999;274:270-274.

137. Kumar SG, Rahman MA, Lee SH, Hwang HS, Kim HA, Yun JW: Plasma proteome analysis for anti-obesity and anti-diabetic potentials of chitosan oligosaccharides in ob/ob mice. Proteomics. 2009;9:2149-2162.

138. Li F, Xia K, Sheikh MSA, Cheng J, Li C, Yang T: Retinol binding protein 4 promotes hyperinsulinism-induced proliferation of rat aortic smooth muscle cells. Molecular Medicine Reports. 2014;9:1634-1640.

139. Liu Z, Liu L, Song X, Huo Z, Cong B, Yang F, Peng Y: Effects of follicle-stimulating hormone beta subunit and nuclear receptor coactivator 1 gene polymorphisms and expressions on pink-eyed white mink reproductive traits. Canadian Journal of Animal Science. 2020;100:683-690.

140. Maarman GJ: **The effect of CPT-1 inhibition on myocardial function and resistance to ischemia/reperfusion injury in a rodent model of the metabolic syndrome.** Stellenbosch: University of Stellenbosch, 2010.

141. Maheras AL, Dix B, Carmo OM, Young AE, Gill VN, Sun JL, Booker AR, Thomason HA, Ibrahim AE, Stanislaw L: Genetic pathways of neuroregeneration in a novel mild traumatic brain injury model in adult zebrafish. Eneuro. 2018;5.

142. Nordentoft I, Jeppesen P, Hong J, Abudula R, Hermansen K: Increased insulin sensitivity and changes in the expression profile of key insulin regulatory genes and beta cell transcription factors in diabetic KKAy-mice after feeding with a soy bean protein rich diet high in isoflavone content. Journal of agricultural and food chemistry. 2008;56:4377-4385.

143. Ohki R, Yamamoto K, Ueno S, Mano H, Ikeda U, Shimada K: Effects of olmesartan, an angiotensin II receptor blocker, on mechanically-modulated genes in cardiac myocytes. Cardiovascular Drugs and Therapy. 2003;17:231-236.

144. Olivares-Garcia V, Torre-Villalvazo I, Velázquez-Villegas L, Alemán G, Lara N, López-Romero P, Torres N, Tovar A, Díaz-Villaseñor A: Fasting and postprandial regulation of the intracellular localization of adiponectin and of adipokines secretion by dietary fat in rats. Nutrition & diabetes. 2015;5:e184-e184.

145. Padro T, Cubedo J, Camino S, Bejar MT, Ben-Aicha S, Mendieta G, Carles Escola-Gil J, Escate R, Gutierrez M, Casani L, et al: Detrimental Effect of Hypercholesterolemia on High-Density Lipoprotein Particle Remodeling in Pigs. Journal of the American College of Cardiology. 2017;70:165-178.

146. Pamir N, Pan C, Plubell DL, Hutchins PM, Tang C, Wimberger J, Irwin A, de Aguiar Vallim TQ, Heinecke JW, Lusis AJ: Genetic control of the mouse HDL proteome defines HDL traits, function, and heterogeneity [S]. Journal of lipid research. 2019;60:594-608.

147. Pang T, Li M, Zhang Y, Yong W, Kang H, Yao Y, Hu X: Y Box-Binding Protein 1 Promotes Epithelial-Mesenchymal Transition, Invasion, and Metastasis of Cervical Cancer via Enhancing the Expressions of Snail. Int J Gynecol Cancer. 2017;27:1753-1760.

148. Patel A, Anderson G, Galea GL, Balys M, Sowden JC: A molecular and cellular analysis of human embryonic optic fissure closure related to the eye malformation coloboma. Development. 2020;147:dev193649.

149. Qin B, Polansky MM, Harry D, Anderson RA: Green tea polyphenols improve cardiac muscle mRNA and protein levels of signal pathways related to insulin and lipid metabolism and inflammation in insulin-resistant rats. Mol Nutr Food Res. 2010;54 Suppl 1:S14-23.

150. Quach A: **Investigation of Genome Variations as Potential Markers for Boar Fertility.** 2015.

151. Rabhi N, Hannou SA, Gromada X, Salas E, Yao X, Oger F, Carney C, Lopez-Mejia IC, Durand E, Rabearivelo I: Cdkn2a deficiency promotes adipose tissue browning. Molecular metabolism. 2018;8:65-76.

152. Robich MP, Osipov RM, Chu LM, Han Y, Feng J, Nezafat R, Clements RT, Manning WJ, Sellke FW: Resveratrol modifies risk factors for coronary artery disease in swine with metabolic syndrome and myocardial ischemia. Eur J Pharmacol. 2011;664:45-53.

153. Rokling-Andersen MH, Rustan AC, Wensaas AJ, Kaalhus O, Wergedahl H, Røst TH, Jensen J, Graff BA, Caesar R, Drevon CA: Marine n-3 fatty acids promote size reduction of visceral adipose depots, without altering body weight and composition, in male Wistar rats fed a high-fat diet. British journal of nutrition. 2009;102:995-1006.

154. Rourke J: The Chemerin Receptor GPR1 Signals Through a RhoA/ROCK Pathway and Contributes to Glucose Homeostasis in Obese Mice. 2015.

155. Sabe AA, Elmadhun NY, Robich MP, Dalal RS, Sellke FW: Does resveratrol improve insulin signaling in chronically ischemic myocardium? Journal of Surgical Research. 2013;183:531-536.

156. Sayre B: Comparative genomics for prediction of the relative location of ESTs in the goat genome. Edited by NE Odongo, M Garcia & GJ Viljoen. 2010;175.

157. Tanaka M, Murayama D, Nagashima M, Higashi T, Mawatari K, Matsukawa T, Kato S: Purpurin expression in the zebrafish retina during early development and after optic nerve lesion in adults. Brain research. 2007;1153:34-42.

158. Tvarijonaviciute A, Gutierrez AM, Miller I, Razzazi-Fazeli E, Tecles F, Ceron JJ: A proteomic analysis of serum from dogs before and after a controlled weight-loss program. Domestic Animal Endocrinology. 2012;43:271-277.

159. Uzbekova S, Almiñana-Brines C, Labas V, Teixeira-Gomes A-P, Combes-Soia L, Tsikis G, Carvalho AV, Uzbekov R, Singina G: Protein Cargo of Extracellular Vesicles From Bovine Follicular Fluid and Analysis of Their Origin From Different Ovarian Cells. Frontiers in veterinary science. 2020;7:821.

160. Vilahur G, Cubedo J, Gutierrez M, Casani L, Capdevila A, Pons-Llado G, Carreras F, Hidalgo A, Badimon L: Dyslipidemia impairs high-density lipoprotein cardioprotective effects leading to larger infarcts. HDL-characterization by lipid analysis and differential proteomics. European Heart Journal. 2015;36:354-355.

161. Wagner MJ, Savall J, Kim TH, Schnitzer MJ, Luo L: Skilled reaching tasks for head-fixed mice using a robotic manipulandum. Nature protocols. 2020;15:1237-1254.

162. Weldenegodguad SB: Transcriptome analysis of bovine day 16 conceptus derived after transfer of blastocyst from somatic cell nuclear transfer or in vitro production. 2013.

163. Wergedahl H: Marine n-3 fatty acids promote size reduction of visceral adipose depots, without altering body weight and compo. British Journal of Nutrition. 2009;102:995-1006.

164. Wijekoon EP: **Metabolic and physiological studies in a rat model of type 2 diabetes.** Memorial University of Newfoundland, 2006.

165. Winkler S, Hempel M, Hsu M-J, Gericke M, Kühne H, Brückner S, Erler S, Burkhardt R, Christ B: Immune-Deficient Pfp/Rag2−/− Mice Featured Higher Adipose Tissue Mass and Liver Lipid Accumulation with Growing Age than Wildtype C57BL/6N Mice. Cells. 2019;8:775.

166. Zagorski J, Obraztsova M, Gellar MA, Kline JA, Watts JA: Transcriptional changes in right ventricular tissues are enriched in the outflow tract compared with the apex during chronic pulmonary embolism in rats. Physiological Genomics. 2009;39:61-71.

167. Zhang Y, Yuan M, Li HM, Lao M, Xu Z, Li GP: Testin on Atherosclerosis in Rabbits. Chin Med J (Engl). 2015;128:1662-1665.

168. Zhong G, Hogarth C, Snyder JM, Palau L, Topping T, Huang W, Czuba LC, LaFrance J, Ghiaur G, Isoherranen N: The retinoic acid hydroxylase Cyp26a1 has minor effects on postnatal vitamin A homeostasis, but is required for exogenous atRA clearance. Journal of Biological Chemistry. 2019;294:11166-11179.

169. Zhou W, Ye S-D, Chen C, Wang W: Involvement of RBP4 in diabetic atherosclerosis and the role of vitamin D intervention. Journal of diabetes research. 2018;2018.

170. Brenner AK, Aasebø E, Hernandez-Valladares M, Selheim F, Berven F, Grønningsæter I-S, Bartaula-Brevik S, Bruserud Ø: The capacity of long-term in vitro proliferation of acute myeloid leukemia cells supported only by exogenous cytokines is associated with a patient subset with adverse outcome. Cancers. 2019;11:73.

171. Danzl K, Messner B, Doppler C, Nebert C, Abfalterer A, Sakic A, Temml V, Heinz K, Streitwieser R, Edelmann T, et al: Early inhibition of endothelial retinoid uptake upon myocardial infarction restores cardiac function and prevents cell, tissue, and animal death. Journal of Molecular and Cellular Cardiology. 2019;126:105-117.

172. De La Cruz A, Omidian A, Wilson M, Altintas M, De La Cruz-Munoz N, Nadji M, Garcia-Buitrago M, Nayer A: Tryptase+ SCF+ Mast Cell Accumulation in the Human Liver Correlates with Fibrosis in Non-Alcoholic Fatty Liver Disease. Journal of Hepatology. 2016;64:S472-S473.

173. Dieker J, Iglesias-Guimarais V, Décossas M, Stevenin J, van der Vlag J, Yuste VJ, Muller S: Early apoptotic reorganization of spliceosomal proteins involves caspases, CAD and rearrangement of NuMA. Traffic. 2012;13:257-272.

174. Foltz DR, Jansen LE, Black BE, Bailey AO, Yates JR, 3rd, Cleveland DW: The human CENP-A centromeric nucleosome-associated complex. Nat Cell Biol. 2006;8:458-469.

175. Fremuntova Z, Mosko T, Soukup J, Kucerova J, Kostelanska M, Hanusova ZB, Filipova M, Cervenakova L, Holada K: Changes in cellular prion protein expression, processing and localisation during differentiation of the neuronal cell line CAD 5. Biol Cell. 2020;112:1-21.

176. Furuya A, Kawano F, Nakajima T, Ueda Y, Sato M: Assembly Domain-Based Optogenetic System for the Efficient Control of Cellular Signaling. ACS Synth Biol. 2017;6:1086-1095.

177. Hashemi SH, Li JY, Faigle R, Dahlström A: Adrenergic differentiation and SSR2a receptor expression in CAD-cells cultured in serum-free medium. Neurochem Int. 2003;42:9-17.

178. Kawakami Y, Siddiki MS, Inoue K, Otabayashi H, Yoshida K, Ueda S, Miyasaka H, Maeda I: Application of fluorescent protein-tagged trans factors and immobilized cis elements to monitoring of toxic metals based on in vitro protein-DNA interactions. Biosens Bioelectron. 2010;26:1466-1473.

179. Kohlstedt K, Gershome C, Trouvain C, Hofmann W-K, Fichtlscherer S, Fleming I: Angiotensin-Converting Enzyme (ACE) Inhibitors Modulate Cellular Retinol-Binding Protein 1 and Adiponectin Expression in Adipocytes via the ACE-Dependent Signaling Cascade. Molecular Pharmacology. 2009;75:685-692.

180. Lam E: Targeting SPINK1 in the damaged tumour microenvironment alleviates therapeutic resistance. 2018.

181. Liu QL, Kishi H, Ohtsuka K, Muraguchi A: Heat shock protein 70 binds caspase-activated DNase and enhances its activity in TCR-stimulated T cells. Blood. 2003;102:1788-1796.

182. Lui JH, Nguyen ND, Grutzner SM, Darmanis S, Peixoto D, Wagner MJ, Allen WE, Kebschull JM, Richman EB, Ren J: Differential encoding in prefrontal cortex projection neuron classes across cognitive tasks. Cell. 2021;184:489-506. e426.

183. Ma Y, Zhang J, Xiao Y, Yang Y, Liu C, Peng R, Yang Y, Bravo A, Soberón M, Liu K: The Cadherin Cry1Ac Binding-Region is Necessary for the Cooperative Effect with ABCC2 Transporter Enhancing Insecticidal Activity of Bacillus thuringiensis Cry1Ac Toxin. Toxins (Basel). 2019;11.

184. Marchiano S: CLINICAL AND EXPERIMENTAL EVIDENCES OF DIRECT VASCULAR EFFECT OF PROPROTEIN CONVERTASE SUBTILISIN/KEXIN TYPE 9. 2018.

185. McClelland SE, Borusu S, Amaro AC, Winter JR, Belwal M, McAinsh AD, Meraldi P: The CENP-A NAC/CAD kinetochore complex controls chromosome congression and spindle bipolarity. Embo j. 2007;26:5033-5047.

186. Mederer T: **Identification and characterization of novel candidate genes for Hirschsprung's disease-a developmental disorder of the enteric nervous system.** 2019.

187. Michielin F, Giobbe GG, Luni C, Hu Q, Maroni I, Orford MR, Manfredi A, Di Filippo L, David AL, Cacchiarelli D: The Microfluidic Environment Reveals a Hidden Role of Self-Organizing Extracellular Matrix in Hepatic Commitment and Organoid Formation of hiPSCs. Cell Reports. 2020;33:108453.

188. Mohd-Sarip A, Teeuwssen M, Bot AG, De Herdt MJ, Willems SM, de Jong RJB, Looijenga LH, Zatreanu D, Bezstarosti K, van Riet J: DOC1-dependent recruitment of NURD reveals antagonism with SWI/SNF during epithelial-mesenchymal transition in oral cancer cells. Cell reports. 2017;20:61-75.

189. Moreira IS, Sensoy O: Modulation of Protein-Protein Interactions for the Development of Effective Therapeutics - From a Joint Perspective of Experiment and Computation. Curr Top Med Chem. 2018;18:645-646.

190. Nakano T, Aikawa M: Supplemental Material Uremic toxin indoxyl sulfate promotes pro-inflammatory macrophage activation via.

191. Ng BG, Wolfe LA, Ichikawa M, Markello T, He M, Tifft CJ, Gahl WA, Freeze HH: Biallelic mutations in CAD, impair de novo pyrimidine biosynthesis and decrease glycosylation precursors. Hum Mol Genet. 2015;24:3050-3057.

192. Öhlinger K, Kolesnik T, Meindl C, Gallé B, Absenger-Novak M, Kolb-Lenz D, Fröhlich E: Air-liquid interface culture changes surface properties of A549 cells. Toxicology In Vitro. 2019;60:369-382.

193. Pan J, Guleria RS, Zhu S, Baker KM: Molecular mechanisms of retinoid receptors in diabetes-induced cardiac remodeling. Journal of clinical medicine. 2014;3:566-594.

194. Philippova M, Joshi MB, Pfaff D, Kyriakakis E, Maslova K, Erne P, Resink TJ: T-cadherin attenuates insulin-dependent signalling, eNOS activation, and angiogenesis in vascular endothelial cells. Cardiovasc Res. 2012;93:498-507.

195. Rai EP: Effect of Lipotoxicity on ER Stress, Autophagy and Apoptosis in Skeletal Muscle and Regulation by Adiponectin. 2014.

196. Reinhold WC, Reimers MA, Lorenzi P, Ho J, Shankavaram UT, Ziegler MS, Bussey KJ, Nishizuka S, Ikediobi O, Pommier YG, Weinstein JN: Multifactorial regulation of E-cadherin expression: an integrative study. Mol Cancer Ther. 2010;9:1-16.

197. Sato T, Akasu H, Shimono W, Matsu C, Fujiwara Y, Shibagaki Y, Heard JJ, Tamanoi F, Hattori S: Rheb protein binds CAD (carbamoyl-phosphate synthetase 2, aspartate transcarbamoylase, and dihydroorotase) protein in a GTP- and effector domain-dependent manner and influences its cellular localization and carbamoyl-phosphate synthetase (CPSase) activity. J Biol Chem. 2015;290:1096-1105.

198. Schwappacher R, Rangaswami H, Su-Yuo J, Hassad A, Spitler R, Casteel DE: cGMP-dependent protein kinase Iβ regulates breast cancer cell migration and invasion via interaction with the actin/myosin-associated protein caldesmon. J Cell Sci. 2013;126:1626-1636.

199. Seldin MM, Koplev S, Rajbhandari P, Vergnes L, Rosenberg GM, Meng Y, Pan C, Phuong TM, Gharakhanian R, Che N: A strategy for discovery of endocrine interactions with application to whole-body metabolism. Cell metabolism. 2018;27:1138-1155. e1136.

200. Sosa MS, Girotti MR, Salvatierra E, Prada F, de Olmo JA, Gallango SJ, Albar JP, Podhajcer OL, Llera AS: Proteomic analysis identified N-cadherin, clusterin, and HSP27 as mediators of SPARC (secreted protein, acidic and rich in cysteines) activity in melanoma cells. Proteomics. 2007;7:4123-4134.

201. Tate T: *Pparg drives luminal differentiation and luminal tumor formation in the urothelium.* Columbia University; 2021.

202. Teo AKK, Tsuneyoshi N, Hoon S, Tan EK, Stanton LW, Wright CV, Dunn NR: PDX1 binds and represses hepatic genes to ensure robust pancreatic commitment in differentiating human embryonic stem cells. Stem cell reports. 2015;4:578-590.

203. van Gurp L: **Characterization of islet cells during development and after transplantation.** Utrecht University, 2017.

204. Vasieva O, Goryanin I: Is there a Function for a Sex Pheromone Precursor? J Integr Bioinform. 2019;16.

205. Zhai L: *Study of Lcn2 in inflammation and characterization of its RNA aptamer.* Iowa State University; 2012.

206. Zhou Y, Yuan J, Qi C, Shao X, Mou S, Ni Z: Calcium dobesilate may alleviate diabetes‑induced endothelial dysfunction and inflammation. Mol Med Rep. 2017;16:8635-8642.

207. ALBINO TSFIP, MM S: Corpo e Movimento. CEP. 15809:144.

208. Ardıç İ, Kaya MG, Yarlıoğlueş M, Karadağ Z, Doğan A, Yıldız H, Doğdu O, Zencir C, Aktaş E, Ergin A: Ekokardiyografi Echocardiography.

209. Beck-Nielsen H: *The Metabolic Syndrome.* Springer; 2013.

210. Bisazza PM, Hillier L, Wilson RK, Fuerstenberg S, Boore J, Searle10 S, Postlethwait JH, Warren WC: Supplementary information for.

211. CARLTON RH: SISA.

212. DELLE CITOCHINE SDE, DELLA METFORMINA E, SULL’ACETILAZIONE DP, UMANE IIP: DIPARTIMENTO DI FARMACIA.

213. Durkin N, Noor S, Desai A, Kurup M: Paediatric Bariatric Surgery and. Core Topics in Anaesthesia and Perioperative Care of the Morbidly Obese Surgical Patient. 2018;228.

214. Eppig JT, Bucan M, Smith BJ, Miller D, Abbott C, Andersson L, Artzt K, Avner P, Balling R, Barlow D: Lee M. Silver. 2000.

215. Fetuin A, Oncostatin M: Adipokine Alternative Name Entrez Gene ID.

216. für Diabetes-Forschung L-Z: Deutschen Diabetes-Zentrum Paul-Langerhans-Gruppe für Integrative Physiologie.

217. Girasole V: SEPSIS AND ADIPONECTIN.

218. GUILLEN MA, VILLENA J, MORELLO E, GAYOSO O: PUBLISHED ONLY.

219. Gupta S, FICP D, Gupta V, Student MGMFY: JCD. JCD. 2014;4.

220. Kinase MC: Sino Biological Inc.(Antikörper| Protein| Elisa| cDNA).

221. Kunikowski W, l Olejnik P, Awrejcewicz J: Journal of Vibration Testing and System Dynamics. Journal of Vibration Testing and System Dynamics. 2018;2:91-107.

222. Michel NA: The Role of Tumor Necrosis Receptor associated Factor (TRAF)-1 in cardio-metabolic disease.

223. MSR R: Sino Biological Inc.(Antikörper| Protein| Elisa| cDNA).

224. Palmetto G: MolDX: Biomarkers in Cardiovascular Risk Assessment.

225. Park J, Rha S, Choi J, Choi B, Choi S, Choi C, Kim E: 336 Renin angiotensin system–old scripts, new movies.

226. PevsnerCopyright J: BLAST! 2010.

227. Smith JD: Whole Genome Expression Differences in Human Left and Right Atria Ascertained by RNA-Sequencing.

228. Townsend W: Western Abstracts Subject Index. 2011.

229. Vivo ATI: Core Clock Gene Expression in Muscle and Adipose Tissue are Cor-related With Metabolic Traits.

230. von Zychlinski A, Kleffmann T: Translational Proteomics.

231. Xu A, Wang Y, Renneberg R, Cautherley G, Chan C: The HKU Scholars Hub The University of Hong Kong 香 港大學 學術庫. Pharmacology. 2007;5:15-25.

232. Yang Y: Glutamine diet supplementation prevents obesity through inhibiting inflammation.

233. Agra RM, Fernández-Trasancos Á, Sierra J, González-Juanatey JR, Eiras S: Differential association of S100A9, an inflammatory marker, and p53, a cell cycle marker, expression with epicardial adipocyte size in patients with cardiovascular disease. Inflammation. 2014;37:1504-1512.

234. Ali EY, Hegazy GA, Hashem EM: Evaluation of irisin, retinol-binding protein 4, and leptin serum levels as biomarkers of macrovascular complications involvement in Saudi type 2 diabetes mellitus. A case-control study. Saudi Medical Journal. 2020;41:1369.

235. Aliasghari F, Aliasgharzadeh S, Faghfouri AH, Mahdavi R, Yagin NL: The predictability of the metabolic syndrome by adipokines. Nutrition & Food Science. 2020.

236. Aust G, Uptaite-Patapoviene M, Scholz M, Richter O, Rohm S, Blueher M: Circulating Nampt and RBP4 levels in patients with carotid stenosis undergoing carotid endarterectomy (CEA). Clinica Chimica Acta. 2011;412:1195-1200.

237. Bakshi S, Schmidt H, Baskin A, Croniger C, Thompson C, Bonfield T, Fletcher D, Berger N: Sexual dimorphism in developmental and diet‐dependent circulating retinol binding protein 4. Obesity science & practice. 2018;4:526-534.

238. Baran A, Świderska M, Bacharewicz-Szczerbicka J, Myśliwiec H, Flisiak I: Serum fatty acid-binding protein 4 is increased in patients with psoriasis. Lipids. 2017;52:51-60.

239. Boutari C, Perakakis N, Mantzoros CS: Association of adipokines with development and progression of nonalcoholic fatty liver disease. Endocrinology and Metabolism. 2018;33:33-43.

240. Chavarria N, Kato TS, Khan R, Chokshi A, Collado E, Akashi H, Takayama H, Naka Y, Farr M, Mancini D: Increased levels of retinol binding protein 4 in patients with advanced heart failure correct after hemodynamic improvement through ventricular assist device placement. Circulation Journal. 2012;CJ-12-0350.

241. Cubedo J, Padró T, Formiga F, Ferrer A, Padrós G, Peña E, Badimon L: Inflammation and hemostasis in older octogenarians: implication in 5-year survival. Transl Res. 2017;185:34-46.e39.

242. Dessein PH, Tsang L, Norton GR, Woodiwiss AJ, Solomon A: Retinol binding protein 4 concentrations relate to enhanced atherosclerosis in obese patients with rheumatoid arthritis. PLoS One. 2014;9:e92739.

243. Gayathri R: **Serum retinol binding protein 4 (RBP4) level in patients with gestational diabetes mellitus.** Madras Medical College, Chennai, 2018.

244. Gerdes S, Osadtschy S, Rostami-Yazdi M, Buhles N, Weichenthal M, Mrowietz U: Leptin, adiponectin, visfatin and retinol-binding protein-4 - mediators of comorbidities in patients with psoriasis? Exp Dermatol. 2012;21:43-47.

245. Güdücü N, Görmüş U, Kavak ZN, İşçi H, Yiğiter A, Dünder İ: Retinol-binding protein 4 is elevated and is associated with free testosterone and TSH in postmenopausal women. Journal of endocrinological investigation. 2013;36:831-834.

246. Güdücü N, Görmüş U, Telatar B, Dünder İ: Retinol-binding protein 4, as a negative acute-phase reactant in polycystic ovary syndrome. 2014.

247. Jabbari S, Hedayati M, Yaghmaei P, Parivar K: Medullary Thyroid Carcinoma-Circulating Status of Vaspin and Retinol Binding Protein-4 in Iranian Patients. Asian Pacific journal of cancer prevention. 2015;16:6507-6512.

248. Junjun W, Jia W, Jiaxi S, Dongmei N, Yonghui S, Fang Z, Xin Z: Associations of RBP 4 with lipid metabolism and renal function in diabetes mellitus. European journal of lipid science and technology. 2013;115:831-837.

249. Kistorp CN, Linneberg A, Jorgen-Sen T, Hess G, Faber J, Hildebrandt P: Retinol-binding protein 4 is associated with parameters reflecting insulin resistance, but is not predictive of outcome in the general population. Diabetes. 2008;57:A285-A285.

250. Klisic A, Kotur-Stevuljevic J, Kavaric N, Matic M: Relationship between cystatin C, retinol-binding protein 4 and Framingham risk score in healthy postmenopausal women. Archives of Iranian medicine. 2016;19:0-0.

251. Kocełak P, Owczarek A, Bożentowicz-Wikarek M, Brzozowska A, Mossakowska M, Grodzicki T, Więcek A, Chudek J, Olszanecka-Glinianowicz M: PROTEIN 4 (RBP4) IN RELATION TO NUTRITIONAL STATUS AND KIDNEY FUNCTION IN ELDERLY POPULATION OF POLSENIOR STUDY. 2017.

252. Liang W, dong Ye D: The potential of adipokines as biomarkers and therapeutic agents for vascular complications in type 2 diabetes mellitus. Cytokine & growth factor reviews. 2019;48:32-39.

253. López-Canoa J, Couselo-Seijas M, Baluja A, González-Melchor L, Rozados A, Llorente-Cortés V, de Gonzalo-Calvo D, Guerra J, Vilades D, Leta R: Sex-related differences of fatty acid-binding protein 4 and leptin levels in atrial fibrillation. EP Europace. 2021;23:682-690.

254. Lv L-q, Tang Y-z, Wang S-q, Xie Y-m, Ge L, Cheng X-m: Research on the Clinical Phenotype of Coronary Heart Disease with Retinol Binding Protein 4, Lipoprotein-related Phospholipase A2, and the Severity of Coronary Artery Lesion. International Journal of Pharmaceutical Research and Allied Sciences. 2017;6:107-112.

255. Majerczyk M, Choręza P, Bożentowicz-Wikarek M, Brzozowska A, Arabzada H, Owczarek A, Mossakowska M, Grodzicki T, Zdrojewski T, Więcek A: Increased plasma RBP4 concentration in older hypertensives is related to the decreased kidney function and the number of antihypertensive drugs—results from the Polsenior substudy. Journal of the American Society of Hypertension. 2017;11:71-80.

256. Majerczyk M, Choręza P, Mizia-Stec K, Bożentowicz-Wikarek M, Brzozowska A, Arabzada H, Owczarek AJ, Szybalska A, Grodzicki T, Więcek A: Plasma level of retinol-binding protein 4, N-terminal proBNP and renal function in older patients hospitalized for heart failure. Cardiorenal medicine. 2018;8:237-248.

257. Majerczyk M, Olszanecka-Glinianowicz M, Puzianowska-Kuznicka M, Chudek J: Retinol-binding protein 4 (RBP4) as the causative factor and marker of vascular injury related to insulin resistance. Postepy Higieny I Medycyny Doswiadczalnej. 2016;70:1267-1275.

258. Mansouri M, Heshmat R, Tabatabaei-Malazy O, Sharifi F, Badamchizadeh Z, Alatab S, Omidfar K, Fakhrzadeh H, Larijani B: The association of carotid intima media thickness with retinol binding protein-4 and total and high molecular weight adiponectin in type 2 diabetic patients. Journal of Diabetes & Metabolic Disorders. 2012;11:1-7.

259. Meena UK, Jain S, Vaid NB, Sharma A, Chawla S, Guleria K, Mehndiratta M: ROLE OF SERUM RETINOL BINDING PROTEIN 4 IN PREECLAMPSIA: A CASE CONTROL STUDY. Indian Obstetrics and Gynaecology. 2018;8.

260. Naji MT, Sami OM, Shams HA, Abdul-Hadi MH, Al-Kuraishy HM, Al-Gareeb AI, Al-Harchan NA-A: The associations between retinol binding protein-4 and cardiometabolic profile: Intertwined-intricate relationship. Biomedical and Biotechnology Research Journal (BBRJ). 2020;4:95.

261. Sasaki M, Otani T, Kawakami M, Ishikawa S-e: Elevation of plasma retinol-binding protein 4 and reduction of plasma adiponectin in subjects with cerebral infarction. Metabolism-Clinical and Experimental. 2010;59:527-532.

262. Straczkowski M, Karczewska-Kupczewska M, Nikolajuk A, Adamska A, Matulewicz N, Zielinska M, Gorska M, Kowalska I: Hyperinsulinemia acutely decreases serum Retinol Binding Protein 4 (RBP4) concentration in lean and obese subjects. Diabetes. 2012;61:A711.

263. Vaisbuch E, Romero R, Mazaki-Tovi S, Erez O, Kim SK, Chaiworapongsa T, Gotsch F, Than NG, Dong Z, Pacora P, et al: Retinol binding protein 4-a novel association with early-onset preeclampsia. Journal of Perinatal Medicine. 2010;38:129-139.

264. Von Jeinsen B, Ritzen L, Vietheer J, Unbehaun C, Weferling M, Liebetrau C, Hamm CW, Rolf A, Keller T: Adipokines retinol binding protein 4 and fatty-acid binding protein 4 in left ventricular hypertrophy. European Heart Journal. 2019;40:350.

265. Wan K, Zeng Z: Polymorphism of RBP4 Locus Is Associated with 5-Year Survival in acute coronary syndrome after coronary revascularization. Journal of the American College of Cardiology. 2015;66:C60-C60.

266. Welsh PI: **Inflammatory markers as novel predictors of cardiovascular disease.** University of Glasgow, 2008.

267. Wu J, Shi Y-h, Niu D-m, Li H-q, Zhang C-n, Wang J-j: Association among retinol-binding protein 4, small dense LDL cholesterol and oxidized LDL levels in dyslipidemia subjects. Clinical biochemistry. 2012;45:619-622.

268. Wu M-Z, Lee C-H, Chen Y, Yu S-Y, Yu Y-J, Ren Q-W, Fong H-YC, Wong P-F, Tse H-F, Lam S-LK: Association between adipocyte fatty acid-binding protein with left ventricular remodelling and diastolic function in type 2 diabetes: a prospective echocardiography study. Cardiovascular diabetology. 2020;19:1-11.

269. Yang M, Weng H, Pei Q, Jing F, Yi Q: The Relationship between Retinol-Binding Protein 4 and Markers of Inflammation and Thrombogenesis in Children with Kawasaki Disease. Mediators of Inflammation. 2021;2021.

270. Yu F, Zhou X, Li Z, Feng X, Liao D, Liu Z, Huang Q, Li X, Yang Q, Xiao B: Diagnostic significance of plasma levels of novel adipokines in patients with symptomatic intra-and extracranial atherosclerotic stenosis. Frontiers in neurology. 2019;10:1228.

271. Zhang M, Chen P, Chen S, Sun Q, Zeng Q, Chen J, Liu Y, Cao X, Ren M, Wang J: The association of new inflammatory markers with type 2 diabetes mellitus and macrovascular complications: a preliminary study. Eur Rev Med Pharmacol Sci. 2014;18:1567-1572.

272. Zhu Y-y, Zhang J-l, Liu L, Han Y, Ge X, Zhao S: Evaluation of serum retinol-binding protein-4 levels as a biomarker of poor short-term prognosis in ischemic stroke. Bioscience reports. 2018;38:BSR20180786.

273. Qi Q, Yu Z, Ye X, Zhao F, Huang P, Hu FB, Franco OH, Wang J, Li H, Liu Y, Lin X: Elevated retinol-binding protein 4 levels are associated with metabolic syndrome in Chinese people. J Clin Endocrinol Metab. 2007;92:4827-4834.

274. Won JC, Park CY, Oh SW, Park SW: Increased plasma levels of retinol-binding protein 4 with visceral obesity is associated with cardiovascular risk factors. J Diabetes Investig. 2012;3:457-463.

275. Kadoglou NP, Lambadiari V, Gastounioti A, Gkekas C, Giannakopoulos TG, Koulia K, Maratou E, Alepaki M, Kakisis J, Karakitsos P: The relationship of novel adipokines, RBP4 and omentin-1, with carotid atherosclerosis severity and vulnerability. Atherosclerosis. 2014;235:606-612.

276. Liu Y, Wang D, Li D, Sun R, Xia M: Associations of retinol-binding protein 4 with oxidative stress, inflammatory markers, and metabolic syndrome in a middle-aged and elderly Chinese population. Diabetol Metab Syndr. 2014;6:25.

277. Yushchuk EN, Sadulaeva IA, Chusova NA: The role of retinol binding protein 4 as a marker of cardiovascular disease risk in patients with arterial hypertension and obesity. Obesity Reviews. 2020;21.

278. Abarna Devi S: **Prevalence of nonalcoholic fatty liver disease and metabolic syndrome in psoriasis in a tertiary health center.** Madras Medical College, Chennai, 2015.

279. ABCA CBO, EFFLUX A-MC: HDL-C PROFILE AND BMI IN DYSLIPIDEMIC CHILDREN.

280. Abdellatif AAT: Study of a new adipocytokine Visfatin in obese males and its relation to type II diabetes mellitus. CU Theses. 2012.

281. Akbar MF: Gene linkage and genomic characteristic analysis between type. group. 2014;4:4.6718.

282. Alfadda AA, Sallam RM, Chishti MA, Moustafa AS, Fatma S, Alomaim WS, Al-Naami MY, Bassas AF, Chrousos GP, Jo H: Differential patterns of serum concentration and adipose tissue expression of chemerin in obesity: adipose depot specificity and gender dimorphism. Molecules and cells. 2012;33:591-596.

283. Ali TM, Al Hadidi K: Chemerin is associated with markers of inflammation and predictors of atherosclerosis in Saudi subjects with metabolic syndrome and type 2 diabetes mellitus. Beni-Suef University Journal of Basic and Applied Sciences. 2013;2:86-95.

284. ALsailawi H, Mudhafar M, Majhool AA, Asaad A: Study of Cystatin C as Early Biomarker of Nephropathy in Patients with Type 2 DM and Risk Stratification in Tarnaka Hospital of Hyderabad City in India. Journal of US-China Medical Science. 2019;16:232-241.

285. Andersen C: Dietary Modulation of the Dynamics between Leukocyte Inflammation, Cholesterol Flux, and HDL function in Metabolic Syndrome. 2013.

286. Anuurad E, Yamasaki M, Shachter N, Pearson TA, Berglund L: ApoE and ApoC-I polymorphisms: association of genotype with cardiovascular disease phenotype in African Americans. Journal of lipid research. 2009;50:1472-1478.

287. Aristoteli L, Hawkins C, Davies M, Kritharides L: Circulating Haptoglobin Isoforms Typical of Patients with Coronary Atherosclerosis Are Related to Variations in Sialylation. Molecular & Cellular Proteomics. 2004;3:S7.

288. Arnaboldi L, Corsini A: Could changes in adiponectin drive the effect of statins on the risk of new-onset diabetes? The case of pitavastatin. Atherosclerosis Supplements. 2015;16:1-27.

289. Ashley DT: **Serum Osteoprotegerin, a Potential Novel Marker of Systemic Inflammation: The Influence of Obesity, Insulin Sensitivity and Oral Glucose Loading on its Circulating Concentrations.** Dublin City University, 2010.

290. Azo Najeeb H, Ahmad Qasim B, Ahmad Mohammed A: Parental history of coronary artery disease among adults with hypothyroidism: Case controlled study. Ann Med Surg (Lond). 2020;60:92-101.

291. Babicz M, Pastwa M, Kozubska-Sobocińska A, Danielak-Czech B, Skrzypczak E, Kropiwiec-Domańska K: Association analysis of GH1 and CRP loci polymorphisms with reproductive traits in native Pulawska gilts and sows. Canadian Journal of Animal Science. 2020;100:650-656.

292. Bacon ER: **Epigenetic regulation of endocrine aging transitions of the Perimenopausal and menopausal brain.** University of Southern California, 2017.

293. Badimon L, Padró T, Cubedo J: Protein changes in non-LDL-lipoproteins in familial hypercholesterolemia: implications in cardiovascular disease manifestation and outcome. Current opinion in lipidology. 2017;28:427-433.

294. Banfi C, Brioschi M, Barcella S, Wait R, Galli S, Tremoli E: PROTEOMIC ANALYSIS OF HUMAN LOW DENSITY LIPOPROTEIN REVEALS THE PRESENCE OF PRENYLCYSTEINE LYASE, A HYDROGEN PEROXIDE-GENERATING ENZYME. Atherosclerosis Supplements. 2009;10.

295. Berezin AE, Berezin AA, Lichtenauer M: Emerging role of adipocyte dysfunction in inducing heart failure among obese patients with prediabetes and known diabetes mellitus. Frontiers in Cardiovascular Medicine. 2020;7.

296. Bilbija D: Alternative Therapeutic Modalities in Treatment of Ischemic Heart Disease: Experimental studies on retinoic acid signaling in myocardial remodelling and remote gene therapy using heme oxygenase-1. 2013.

297. Blaikley J, Sutton P, Walter M, Lapsley M, Norden A, Pugsley W, Unwin R: Tubular proteinuria and enzymuria following open heart surgery. Intensive Care Medicine. 2003;29:1364-1367.

298. Borai IH, Soliman AF, Ahmed HM, Ahmed GF, Kassim SK: Association of MTHFR C677T and ABCA1 G656A polymorphisms with obesity among Egyptian children. Gene Reports. 2018;11:143-149.

299. Borskaya E, Kerbikov O, Panteleev R, Shakhnovich E, Doinichenko N, Krutova T, Averyanov A: P4. 23 HYPOECHGENIC CAROTID PLAQUES ARE MORE MOBILE IN COMPARISON WITH HYPERECHOGENIC ONES. Artery Research. 2012;6:189-190.

300. Brown CE, McCarthy NS, Hughes AD, Sever P, Stalmach A, Mullen W, Dominiczak AF, Sattar N, Mischak H, Thom S: Urinary proteomic biomarkers to predict cardiovascular events. PROTEOMICS–Clinical Applications. 2015;9:610-617.

301. Buccino AP, Kordovan M, Ness TV, Merkt B, Häfliger PD, Fyhn M, Cauwenberghs G, Rotter S, Einevoll GT: Combining biophysical modeling and deep learning for multielectrode array neuron localization and classification. Journal of neurophysiology. 2018;120:1212-1232.

302. Buckner T, Shao B, Eckel RH, Heinecke JW, Bornfeldt KE, Snell-Bergeon J: Association of apolipoprotein C3 with insulin resistance and coronary artery calcium in patients with type 1 diabetes. J Clin Lipidol. 2021;15:235-242.

303. Burgeiro A, Fuhrmann A, Cherian S, Espinoza D, Jarak I, Carvalho RA, Loureiro M, Patrício M, Antunes M, Carvalho E: Glucose uptake and lipid metabolism are impaired in epicardial adipose tissue from heart failure patients with or without diabetes. American journal of physiology-endocrinology and metabolism. 2016;310:E550-E564.

304. Bustamante A, Penalba A, Orset C, Azurmendi L, Llombart V, Simats A, Pecharroman E, Ventura O, Ribo M, Vivien D, et al: Blood Biomarkers to Differentiate Ischemic and Hemorrhagic Strokes. Neurology. 2021;96:E1928-E1939.

305. Canzano P: INFLUENCE OF CHRONIC KIDNEY DISEASE ON THE HAEMOSTATIC PROPERTIES, THE PLATELET TRANSCRIPTOMIC AND PLASMA PROTEOMIC PROFILES OF CORONARY ARTERY DISEASE PATIENTS. 2016.

306. Carrey EA, Hardie DG: Mapping of catalytic domains and phosphorylation sites in the multifunctional pyrimidine-biosynthetic protein CAD. Eur J Biochem. 1988;171:583-588.

307. Cave MC, Hurt RT, Frazier TH, Matheson PJ, Garrison RN, McClain CJ, McClave SA: Obesity, inflammation, and the potential application of pharmaconutrition. Nutrition in Clinical Practice. 2008;23:16-34.

308. Cediel G, Carrasquer A, Gonzalez_Del_Hoyo M, Sanchez R, Boque C, Bardaji A: P6434Early risk stratification of patients with positive troponin and without acute coronary syndrome. European Heart Journal. 2017;38.

309. Chang SH, Chang WL, Lu CC, Tarn WY: Alanine repeats influence protein localization in splicing speckles and paraspeckles. Nucleic Acids Res. 2014;42:13788-13798.

310. Chhabra N: **Developmental gene Pax6 in adult pancreas homeostasis and energy metabolism.** Technische Universität München, 2017.

311. Chrousos GP: Differential patterns of serum concentration and adipose tissue expression of chemerin in obesity: Adipose depot. Mol Cells. 2012;33:591-596.

312. Crotty Alexander L, Drummond C, Hepokoski M: Chronic Inhalation of E-Cigarette Vapor Containing Nicotine Disrupts Airway Barrier Function. 2018.

313. Cubedo J, Padro T, Formiga F, Ferrer A, Padros G, Badimon L: Increased anti-fibrinolytic proteins characterize elderly individuals with un-healthy ageing due to cardiovascular disease and cognitive decline. European Heart Journal. 2014;35:522.

314. Dash S: *RNA-binding protein mediated post-transcriptional control of gene expression in eye development and disease.* University of Delaware; 2018.

315. de Azevedo Rabello FR, dos Santos Pereira SS, Corrêa-Giannella M, Neto DG, Melo ESA, Caramelli B: The impact of bariatric surgery on cardiometabolic profile and adipokine levels. Atherosclerosis. 2016;252:e142.

316. Dogan S, Guven K, Celikbilek M, Deniz K, Saraymen B, Gursoy S: Serum visfatin levels in ulcerative colitis. Journal of clinical laboratory analysis. 2016;30:552-556.

317. Du N, Cui Y, Xie W, Yin C, Gong C, Chen X: Application effect of initiation of enteral nutrition at different time periods after surgery in neonates with complex congenital heart disease A retrospective analysis. Medicine. 2021;100.

318. Duan CA, Pan Y, Ma G, Zhou T, Zhang S, Xu N-l: A cortico-collicular pathway for motor planning in a memory-dependent perceptual decision task. Nature Communications. 2021;12:1-16.

319. Dudek AH: **The Induction of an Antiviral State by MxA Requires the Presence of the ATPases SMARCA2 and SMARCA4, which Can be Inactivated by Caspase-mediated Cleavage.** Albert-Ludwigs-Universität Freiburg im Breisgau, 2017.

320. Eliashevich S, Drapkina O, Shoibonov B: A new method to determine the modified LDL atherogenicity among patients at low cardiovascular risk. Atherosclerosis. 2017;263:e200.

321. Elsevier A, Neo7logix B, Yuryev A, Catanzaro J, Khan MSS: Combined Analysis of Tumor RNAseq, Urine Proteomics and WES Profiles from Patient with Gallbladder Cancer. 2019.

322. Fang X, Zhang T, Yang M, Li L, Zhang C, Hu W, Fan X, Liu H, Zhu Z, Liu D: High circulating alarin levels are associated with presence of metabolic syndrome. Cellular Physiology and Biochemistry. 2018;51:2041-2051.

323. Farmakis D, Koeck T, Mullen W, Parissis J, Gogas BD, Nikolaou M, Lekakis J, Mischak H, Filippatos G: Urine proteome analysis in heart failure with reduced ejection fraction complicated by chronic kidney disease: feasibility, and clinical and pathogenetic correlates. European journal of heart failure. 2016;18:822-829.

324. Feltes BC, de Faria Poloni J, Nunes IJG, Bonatto D: Fetal alcohol syndrome, chemo-biology and OMICS: ethanol effects on vitamin metabolism during neurodevelopment as measured by systems biology analysis. Omics: a journal of integrative biology. 2014;18:344-363.

325. Feng Y, Kang K, Xue Q, Chen Y, Wang W, Cao J: Value of plasma homocysteine to predict stroke, cardiovascular diseases, and new-onset hypertension: A retrospective cohort study. Medicine. 2020;99.

326. Fernández‐Trasancos Á, Fandiño‐Vaquero R, Agra RM, Fernández ÁL, Viñuela JE, González‐Juanatey JR, Eiras S: Impaired adipogenesis and insulin resistance in epicardial fat‐mesenchymal cells from patients with cardiovascular disease. Journal of cellular physiology. 2014;229:1722-1730.

327. Ford K: The Development of a Nucleic Acid-Directed Switch and Proximity Dependent Protein Assays Using Electrochemical Techniques. 2018.

328. Franco-Martínez L, Gelemanović A, Horvatić A, Contreras-Aguilar MD, Dąbrowski R, Mrljak V, Cerón JJ, Martínez-Subiela S, Tvarijonaviciute A: Changes in Serum and Salivary Proteins in Canine Mammary Tumors. Animals. 2020;10:741.

329. Fujii R, Yamada H, Munetsuna E, Yamazaki M, Ando Y, Mizuno G, Tsuboi Y, Ohashi K, Ishikawa H, Hagiwara C, et al: Associations between dietary vitamin intake, ABCA1 gene promoter DNA methylation, and lipid profiles in a Japanese population. American Journal of Clinical Nutrition. 2019;110:1213-1219.

330. Garay Baquero DJ: **Protemic discovery and validation of diagnostic plasma biomarkers for pulmonary tuberculosis.** University of Southampton, 2018.

331. Gasperin LdOF: **Association between cigarette smoking and body composition among Austrian adults.** uniwien, 2012.

332. Gehrau R, Maluf D, Cathro H, King A, Suh J, Brayman K, Ladie D, Mas V: Portrait of CAD with IF-TA Development Post Kidney Transplantation. Proteomics as Noninvasive Biomarkers.: Abstract# 172. American Journal of Transplantation. 2013;13.

333. Gillberg L, Perfilyev A, Brøns C, Thomasen M, Grunnet LG, Volkov P, Rosqvist F, Iggman D, Dahlman I, Risérus U: Adipose tissue transcriptomics and epigenomics in low birthweight men and controls: role of high-fat overfeeding. Diabetologia. 2016;59:799-812.

334. Gluba-Brzózka A, Franczyk B, Banach M, Rysz-Górzyńska M: Do HDL and LDL subfractions play a role in atherosclerosis in end-stage renal disease (ESRD) patients? International urology and nephrology. 2017;49:155-164.

335. Gunduztepe Y, Bukan NC, Zorlu E, Erel O: The evaluation of thiol-disulfite balance, ischemia albumin modification and seruloplazmine as a new oxidative stress in mild cognitive impairment and early stage alzheimer’s disease patients. Education. 1985;1991.

336. Hannou SA, Wouters K, Paumelle R, Staels B: Functional genomics of the CDKN2A/B locus in cardiovascular and metabolic disease: what have we learned from GWASs? Trends in Endocrinology & Metabolism. 2015;26:176-184.

337. Hansen T, Ahlström H, Söderberg S, Hulthe J, Wikström J, Lind L, Johansson L: Visceral adipose tissue, adiponectin levels and insulin resistance are related to atherosclerosis as assessed by whole-body magnetic resonance angiography in an elderly population. Atherosclerosis. 2009;205:163-167.

338. Heo JM, Park JH, Kim JH, You SH, Kim JS, Ahn C-M, Hong SJ, Shin K-H, Lim D-S: Comparison of inflammatory markers between diabetic and nondiabetic ST segment elevation myocardial infarction. Journal of Cardiology. 2012;60:204-209.

339. Hepler C: **Regulation of Visceral Adipose Tissue Development and Remodeling in Obesity.** 2018.

340. Hepler C, Shan B, Zhang Q, Henry GH, Shao M, Vishvanath L, Ghaben AL, Mobley AB, Strand DW, Hon GC: Single-Cell RNA Sequencing Identifies Functionally Distinct Fibro-inflammatory and Adipogenic Pdgfrβ Progenitor Subpopulations in Visceral Adipose Tissue. Available at SSRN 3188404. 2018.

341. Hong SJ, Choi SC, Cho JY, Joo HJ, Park JH, Yu CW, Lim D-S: Pioglitazone Increases Circulating MicroRNA-24 With Decrease in Coronary Neointimal Hyperplasia in Type 2 Diabetic Patients–Optical Coherence Tomography Analysis–. Circulation Journal. 2015;CJ-14-0964.

342. Huang H, Liu D, Dong Y: GW24-e2463 Relationship between resting heart rate reduction and progression of coronary atherosclerosis in established coronary artery disease patients. Heart. 2013;99:A157-A158.

343. Hulver MW, Zheng D, Tanner CJ, Houmard JA, Kraus WE, Slentz CA, Sinha MK, Pories WJ, MacDonald KG, Dohm GL: Adiponectin is not altered with exercise training despite enhanced insulin action. American Journal of Physiology-Endocrinology And Metabolism. 2002;283:E861-E865.

344. Hwang J-H, Chen J-C, Yang W-S, Liu T-C: Waist circumference is associated with pitch pattern sequence score in older male adults. International journal of audiology. 2012;51:920-925.

345. Hwang J-H, Tseng F-Y, Liu T-C, Yang W-S: No association between plasma adiponectin levels and central auditory function in adults. Metabolic brain disease. 2015;30:191-196.

346. Imler T, Chalasani N, Liangpunsakul S: Incidence and Demographics of Alcoholic Hepatitis Using a Large Health Information Exchange. Am J Gastroenterol. 2012;107:S142-S200.

347. Järup L, Carlsson MD, Elinder CG, Hellström L, Persson B, Schütz A: Enzymuria in a population living near a cadmium battery plant. Occupational and environmental medicine. 1995;52:770-772.

348. Jernås M: Microarray Analysis in Human Adipose Tissue and Adipocytes.

349. Jernås M: *Microarray analysis of gene expression in human adipocytes and adipose tissue.* Inst of Medicine. Dept of Molecular and Clinical Medicine; 2008.

350. Jerry Abraham J: **Prevalence of metabolic syndrome in young acute coronary syndrome patients.** Tirunelveli Medical College, Tirunelveli, 2018.

351. Kadoglou NP, Kassimis G, Patsourakos N, Kanonidis I, Valsami G: Omentin-1 and vaspin serum levels in patients with pre-clinical carotid atherosclerosis and the effect of statin therapy on them. Cytokine. 2021;138:155364.

352. Kallempudi SS: **Development of a new biosensor array and lab-on-a-chip for portable applications using a label-free detection method.** 2011.

353. Kammerer A, Staab H, Herberg M, Kerner C, Klöting N, Aust G: Increased circulating chemerin in patients with advanced carotid stenosis. BMC cardiovascular disorders. 2018;18:1-7.

354. Katugampola SD, Kuc RE, Maguire JJ, Davenport AP: G-protein-coupled receptors in human atherosclerosis: comparison of vasoconstrictors (endothelin and thromboxane) with recently de-orphanized (urotensin-II, apelin and ghrelin) receptors. Clin Sci (Lond). 2002;103 Suppl 48:171s-175s.

355. Kaur P, Rizk NM, Ibrahim S, Younes N, Uppal A, Dennis K, Karve T, Blakeslee K, Kwagyan J, Zirie M: iTRAQ-based quantitative protein expression profiling and MRM verification of markers in type 2 diabetes. Journal of proteome research. 2012;11:5527-5539.

356. Kher K, Mietus-Snyder M: **Kidney disease associated with diabetes mellitus and metabolic syndrome.** In *Clinical Pediatric Nephrology.* CRC Press; 2016: 549-568

357. Khoueiry G, Abdallah M, Saiful F, Abi Rafeh N, Raza M, Bhat T, El-Sayegh S, Kalantar-Zadeh K, Lafferty J: High-density lipoprotein in uremic patients: metabolism, impairment, and therapy. International urology and nephrology. 2014;46:27-39.

358. Kim H-J, Yoo H-S, Kim P-K, Kim M-R, Lee H-W, Kim C-W: Comparative analysis of serum proteomes of patients with cardiovascular disease. Clinical Biochemistry. 2011;44:178-184.

359. Kim N, Nguyen M, Do D, Nguyen Q, Nguyen T, Truong T: CYP2C19 genetic polymorphism on clopidogrel response in vietnamese patients with coronary stenting. Atherosclerosis. 2020;315:e83-e84.

360. Kjellmo C, Nestvold T, Lappegård K, Hovland A, Mathisen M: Bariatric surgery improves lipid profile in morbidly obese patients by lowering LDL-C and increasing HDL-C and the large HDL-particles. Atherosclerosis. 2016;252:e142-e143.

361. Kontush A: Identifying new Risk Markers and Potential Targets: The Value of the Proteome. Cardiovascular drugs and therapy. 2016;30:13-18.

362. Kouyama K, Miyake K, Zenibayashi M, Hirota Y, Teranishi T, Tamori Y, Kanda H, Sakaguchi K, Ohara T, Kasuga M: Association of serum MCP-1 concentration and MCP-1 polymorphism with insulin resistance in Japanese individuals with obese type 2 diabetes. Kobe J Med Sci. 2008;53:345-354.

363. Krisp C: Proteomic profiling of exudates from diabetic foot ulcers and acute wounds using mass spectrometry. 2013.

364. Kutsuna N, Makita K, Goto K, Hirayama K, Kido G, Kagawa Y: **Fluctuations of Nutrition-Associated Markers After Decompressive Hemicraniectomy in Middle Cerebral Artery Occlusion Patients.** In *Oxygen Transport to Tissue Xl.* *Volume* 1072. Edited by Thews O, LaManna JC, Harrison DK; 2018: 33-38: *Advances in Experimental Medicine and Biology*].

365. Lachine NA, Elnekiedy AA, Megallaa MH, Khalil GI, Sadaka MA, Rohoma KH, Kassab HS: Serum chemerin and high-sensitivity C reactive protein as markers of subclinical atherosclerosis in Egyptian patients with type 2 diabetes. Therapeutic advances in endocrinology and metabolism. 2016;7:47-56.

366. Ladjouze A, Kedji L, Maoudj A, Berkouk K, Bensmina M, Aboura R, Dahmane N, Anane T, Laraba A: Neonatal hypocalcaemia revealing maternal calcium metabolism disorder. Horm Res. 2012;78:1.

367. Lando D, Peet DJ, Whelan DA, Gorman JJ, Whitelaw ML: Asparagine hydroxylation of the HIF transactivation domain a hypoxic switch. Science. 2002;295:858-861.

368. Langheim S, Dreas L, Veschini L, Maisano F, Foglieni C, Ferrarello S, Sinagra G, Zingone B, Alfieri O, Ferrero E: Increased expression and secretion of resistin in epicardial adipose tissue of patients with acute coronary syndrome. American Journal of Physiology-Heart and Circulatory Physiology. 2010;298:H746-H753.

369. Lechareas S, Yanni AE, Golemati S, Chatziioannou A, Perrea D: Ultrasound and biochemical diagnostic tools for the characterization of vulnerable carotid atherosclerotic plaque. Ultrasound in Medicine & Biology. 2016;42:31-43.

370. Ledoux S, Coupaye M, Essig M, Msika S, Roy C, Queguiner I, Clerici C, Larger E: Do fat distribution still predict metabolic disturbances in women with severe obesity?

371. Lee SD: **Rural-urban differences in prevalence of diagnosed dyslipidemia in Newfoundland: findings from the Eastern Health laboratory information system.** Memorial University of Newfoundland, 2017.

372. Lee Y-h, Lee S-H, Jung ES, Kim J-S, Shim CY, Ko Y-G, Choi D, Jang Y, Chung N, Ha J-W: Visceral adiposity and the severity of coronary artery disease in middle-aged subjects with normal waist circumference and its relation with lipocalin-2 and MCP-1. Atherosclerosis. 2010;213:592-597.

373. Legry V, Bokor S, Cottel D, Beghin L, Catasta G, Molnar D, Moreno LA, Amouyel P, Dallongeville J, Meirhaeghe A: " Impact of Angiopoietin-like proteins 3 and 4 genetic polymorphisms on adiposity and lipid metabolism in European adult and adolescent samples. Obesity Facts: the European journal of obesity. 2009;2:183 (T184: PO. 116).

374. LeMaire SA, Jones MM, Conklin LD, Carter SA, Criddell MD, Wang XL, Raskin SA, Coselli JS: Randomized comparison of cold blood and cold crystalloid renal perfusion for renal protection during thoracoabdominal aortic aneurysm repair. Journal of Vascular Surgery. 2009;49:11-19.

375. Leotta CG: Localizzazione intranucleare ed espressione del gene HLXB9 nel differenziamento neuronale in vitro e nell insorgenza di disordini mielodi. 2014.

376. Li P, Lv B, Jiang X, Wang T, Ma X, Chang N, Wang X, Gao X: Identification of NF-κB inhibitors following Shenfu injection and bioactivity-integrated UPLC/Q-TOF-MS and screening for related anti-inflammatory targets in vitro and in silico. Journal of ethnopharmacology. 2016;194:658-667.

377. Li Y-H, Cao Y, Liu F, Zhao Q, Adi D, Huo Q, Liu Z, Luo J-Y, Fang B-B, Tian T: Visualization and Analysis of Gene Expression in Stanford Type A Aortic Dissection Tissue Section by Spatial Transcriptomics. Frontiers in genetics. 2021;12:1153.

378. Lin YH, Ho Y-L, Lee J-K, Huang H-L, Huang K-C, Chen M-F: Plasma leptin levels and digital pulse volume in obese patients without metabolic syndrome - A pilot study. Clinica Chimica Acta. 2011;412:730-734.

379. Lindström I, Andersson E, Dogan J: The transition state structure for binding between TAZ1 of CBP and the disordered Hif-1α CAD. Sci Rep. 2018;8:7872.

380. Liu H, Zeng L, Yang K, Zhang G: A network pharmacology approach to explore the pharmacological mechanism of xiaoyao powder on anovulatory infertility. Evidence-Based Complementary and Alternative Medicine. 2016;2016.

381. Luke BL: **Architecture of an Integrated Microelectronic Warfare System-on-a-Chip and Design of Key Components.** NAVAL POSTGRADUATE SCHOOL MONTEREY CA DEPT OF ELECTRICAL AND COMPUTER …; 2004.

382. Madani R: **Signals from adipose tissue in morbid obesity and effect on depot specific differences.** UCL (University College London), 2009.

383. Mahato K, Lodh M, Parida A, Ahirwar AK, Datta RR, Goswami B: Interplay between PAPP-A, inflammation and adiposity in patients with angiographically proven acute coronary syndrome (ACS). Hormone molecular biology and clinical investigation. 2017;31.

384. Mancini A, Leo F, Di Segni C, Raimondo S: Relationship Between Hormonal Milieu and Oxidative Stress in Childhood Obesity: A Physiopathological Basis for Antioxidant Treatment and Prevention of Cardiovascular Risk. Anti Obes Drug Discov Dev. 2017;3:149.

385. Manea V, Pop C, Pop L: High prevalence of non dippers hypertensive diabetic patients: The role of ambulatory blood pressure monitoring in effective treatment. Atherosclerosis. 2016;252:e142.

386. Marcadenti A, Fuchs SC, Moreira LB, Wiehe M, Gus M, Fuchs FD: Accuracy of anthropometric indexes of obesity to predict diabetes mellitus type 2 among men and women with hypertension. American journal of hypertension. 2011;24:175-180.

387. Margerit M, Nguyen P, Pereira B, Allanore Y, Le Loët X, Rat A, Soubrier M: THU0068 Prevalence of dyslipidaemia in an early arthritis rheumatoid cohort (ESPOIR cohort). Lack of improvement after 4 years of follow-up. Annals of the Rheumatic Diseases. 2013;71:175-176.

388. Maria Agra R, Fernandez-Trasancos A, Sierra J, Ramon Gonzalez-Juanatey J, Eiras S: Differential Association of S100A9, an Inflammatory Marker, and p53, a Cell Cycle Marker, Expression with Epicardial Adipocyte Size in Patients with Cardiovascular Disease. Inflammation. 2014;37:1504-1512.

389. MED S: **Characterisation of epicardial adipose tissue and myocardial fat infiltration in humans.** UNIVERSITY OF VERONA, 2013.

390. Melaku LS, Debela N: Molecular description of fibroblast growth factor and mechanisms by which fibroblast growth factor-21 mediating biological actions and acting as a biologic biomarker of cardiovascular diseases. Indian Journal of Health Sciences and Biomedical Research (KLEU). 2020;13:68.

391. Min H: **Cancer Biomarker Discovery Using N-terminal Peptides and Multiple Reaction Monitoring-MS Techniques.** 서울대학교 대학원, 2015.

392. Min KB, Min JY: Relation of serum vitamin A levels to all-cause and cause-specific mortality among older adults in the NHANES III population. Nutrition Metabolism and Cardiovascular Diseases. 2014;24:1197-1203.

393. Montoro JM, García JF, Vega MM, Fuentes MD, Tinahones F: PECAM-1 levels are increased in morbidly obese young men. Atherosclerosis. 2020;315:e59-e60.

394. Mukker J: **Pharmacokinetic and pharmacodynamic studies on flaxseed lignans.** University of Saskatchewan, 2013.

395. Murata M, Tamemoto H, Otani T, Jinbo S, Ikeda N, Kawakami M, Ishikawa S-e: Endothelial impairment and bone marrow-derived CD34(+)/133(+) cells in diabetic patients with erectile dysfunction. Journal of Diabetes Investigation. 2012;3:526-533.

396. Nakashima A, Kawanishi I, Eguchi S, Yu EH, Eguchi S, Oshiro N, Yoshino K, Kikkawa U, Yonezawa K: Association of CAD, a multifunctional protein involved in pyrimidine synthesis, with mLST8, a component of the mTOR complexes. J Biomed Sci. 2013;20:24.

397. Nasir Y, Farzollahpour F, Mirzababaei A, Maghbooli Z, Mirzaei K: Associations of dietary fats intake and adipokines levels in obese women. Clinical Nutrition ESPEN. 2021;43:390-396.

398. Natsukawa T, Maeda N, Fukuda S, Yamaoka M, Fujishima Y, Nagao H, Sato F, Nishizawa H, Sawano H, Hayashi Y: Significant association of serum adiponectin and creatine kinase-MB levels in ST-segment elevation myocardial infarction. Journal of atherosclerosis and thrombosis. 2017;38232.

399. Nduhirabandi F: **The role of melatonin in cardioprotection: an investigation into the mechanisms involved in glucose homeostasis, microvascular endothelial function and mitochondrial function in normal and insulin resistant states.** Stellenbosch: Stellenbosch University, 2014.

400. Nguyen QN: Causal Effect of Body Mass Index on Thyroid Cancer Risk: A Mendelian Randomization Analysis. 2018.

401. Niemira M, Collin F, Szalkowska A, Bielska A, Chwialkowska K, Reszec J, Niklinski J, Kwasniewski M, Kretowski A: Supplementary Materials: Molecular Signature of Subtypes of Non-Small Cell Lung Cancer by Large-Scale Transcriptional Profiling: Identification of Key Modules and Genes by Weighted Gene Co-Expression Network Analysis (WGCNA).

402. Nishijo M, Nakagawa H, Morikawa Y, Tabata M, Senma M, Miura K, Takahara H, Kawano S, Nishi M, Mizukoshi K: Mortality of inhabitants in an area polluted by cadmium: 15 year follow up. Occupational and Environmental Medicine. 1995;52:181-184.

403. Oberbach A, Blueher M, Wirth H, Till H, Kovacs P, Kullnick Y, Schlichting N, Tomm JM, Rolle-Kampczyk U, Murugaiyan J, et al: Combined Proteomic and Metabolomic Profiling of Serum Reveals Association of the Complement System with Obesity and Identifies Novel Markers of Body Fat Mass Changes. Journal of Proteome Research. 2011;10:4769-4788.

404. Okada K, Furusyo N, Murata M, Sawayama Y, Kainuma M, Hayashi J: A hypertriglyceridemic state increases high sensitivity C-reactive protein of Japanese men with normal glucose tolerance. Endocrine. 2012;41:96-102.

405. Okada T, Ohama T, Takafuji K, Kanno K, Matsuda H, Sairyo M, Zhu Y, Saga A, Kobayashi T, Masuda D: Shotgun proteomic analysis reveals proteome alterations in HDL of patients with cholesteryl ester transfer protein deficiency. Journal of clinical lipidology. 2019;13:317-325.

406. Olechnovič K, Venclovas Č: Contact Area-Based Structural Analysis of Proteins and Their Complexes Using CAD-Score. Methods Mol Biol. 2020;2112:75-90.

407. Olsen T, Vinknes KJ, Blomhoff R, Lysne V, Midttun Ø, Dhar I, Ueland PM, Svingen GF, Pedersen EK, Drevon CA: Creatinine, total cysteine and uric acid are associated with serum retinol in patients with cardiovascular disease. European journal of nutrition. 2020;59:2383-2393.

408. Olsen T, Vinknes KJ, Svingen GF, Pedersen ER, Tell GS, Blomhoff R, Drevon CA, Ueland PM, Midttun Ø, Refsum H: Cardiovascular disease risk associated with serum apolipoprotein B is modified by serum vitamin A. Atherosclerosis. 2017;265:325-330.

409. Otani H: Oxidative stress as pathogenesis of cardiovascular risk associated with metabolic syndrome. Antioxidants & redox signaling. 2011;15:1911-1926.

410. Otomo T, Sakahira H, Uegaki K, Nagata S, Yamazaki T: Structure of the heterodimeric complex between CAD domains of CAD and ICAD. Nat Struct Biol. 2000;7:658-662.

411. Pamir N, Pan C, Plubell DL, Hutchins PM, Tang C, Wimberger J, Irwin A, de Aguiar Vallim TQ, Heinecke JW, Lusis AJ: Genetic control of the HDL proteome. bioRxiv. 2018;405811.

412. Paoloni M, Davis S, Lana S, Withrow S, Sangiorgi L, Picci P, Hewitt S, Triche T, Meltzer P, Khanna C: Canine tumor cross-species genomics uncovers targets linked to osteosarcoma progression. BMC genomics. 2009;10:1-13.

413. Pappa E, Vougas K, Zoidakis J, Papaioannou W, Rahiotis C, Vastardis H: Downregulation of Salivary Proteins, Protective against Dental Caries, in Type 1 Diabetes. Proteomes. 2021;9:33.

414. Park J, Rha S, Choi J, Choi B, Choi S, Choi C, Kim E, Park C, Seo H, Oh D: Angiotensin converting enzyme inhibitor versus angiotensin receptor blocker on the incidence of new-onset diabetes mellitus in Asian population. European Heart Journal. 2013;34.

415. Patel A, Thompson A, Abdelmalek L, Adams-Huet B, Jialal I: The relationship between tyramine levels and inflammation in metabolic syndrome. Hormone Molecular Biology and Clinical Investigation. 2019;E1782-E1788.

416. Paul J, Maiti K, Read M, Hure A, Smith J, Chan EC, Smith R: Phasic phosphorylation of caldesmon and ERK 1/2 during contractions in human myometrium. PLoS One. 2011;6:e21542.

417. Peters JM, Blainey PC, Bryson BD: Consensus transcriptional states describe human mononuclear phagocyte diversity in the lung across health and disease. bioRxiv. 2020.

418. Pinegina N, Louinova M, Vagida M, Shpektor A, Vasilieva E, Margolis LB: Different subpopulations of monocytes in platelet-monocyte complexes in patients with acute coronary syndrome. Atherosclerosis. 2017;263:e164-e165.

419. Preisner T: Efficient One-Shot Function Tracing in the Linux Kernel. 2019.

420. Promintzer M, Krebs M, Todoric J, Luger A, Bischof MG, Nowotny P, Wagner O, Esterbauer H, Anderwald C: Insulin resistance is unrelated to circulating retinol binding protein and protein C inhibitor. J Clin Endocrinol Metab. 2007;92:4306-4312.

421. Puig N, Creus A, Miñambres I, Gil P, Perez A, Sanchez-Quesada J, Benitez S: Epicardial adipose tissue from type 2 diabetic patients displays features ascribed to metabolic alterations and inflammation. Atherosclerosis. 2020;315:e70.

422. Pujia A, De Angelis F, Scumaci D, Gaspari M, Liberale C, Candeloro P, Cuda G, Di Fabrizio E: Highly efficient human serum filtration with water-soluble nanoporous nanoparticles. International journal of nanomedicine. 2010;5:1005.

423. Qian Z, Gong L, Mou Y, Han Y, Zheng S: MicroRNA‑203a‑3p is a candidate tumor suppressor that targets thrombospondin 2 in colorectal carcinoma. Oncology reports. 2019;42:1825-1832.

424. QIN DOS: EXPRESSION AND SECRETION OF HUMAN VISFATIN, AND ITS CORRELATION WITH METABOLIC PARAMETERS IN OBESE CHILDREN. 2012.

425. Ramos Gordillo M, Cabrera Franquiz F, Perez Lorenzo Y, Cabrera Oliva J, Yedra M, Sanchez Villegas A: VALIDATION OF A QUESTIONNAIRE OF LYCOPENE FREQUENCY INTAKE. Nutricion Hospitalaria. 2012;27:1320-1327.

426. Rashad NM, Abd-Elrahman MA, Amal S, Amin AI: Serum visfatin as predictive marker of cardiometabolic risk in women with polycystic ovary syndrome. Middle East Fertility Society Journal. 2018;23:335-341.

427. Reiber I, Mark L, Bajnok L, Paragh G: Comparison of calculated LDL-C levels (Friedewald and Martin/Hopkins estimation) in atherogenic dyslipidemia condition. Atherosclerosis. 2020;315:e70.

428. Riesco Acevedo DG: **New adipokines vaspin and omentin, circulating levels, gene expression in adipose tissue and relationship of circulating levels with nonalcoholic fatty liver disease.** Universitat Rovira i Virgili, 2016.

429. RISK C: OBESITY, CENTRAL OBESITY AND DISTURBANCES IN GLUCOSE METABOLISM IN THE FIN-D2D SURVEY.

430. Rodó Morera J: *Transcriptomic analysis of white and brown adipose tissue during non-shivering thermogenesis.* Universitat Autònoma de Barcelona; 2019.

431. Rodriguez-Mortera R, Caccavello R, Sevilla MG, Gugliucci A: Higher ANGPTL3 and APO CIII levels are associated with APO B48 (chylomicron remnant) dyslipidemia and visceral fat in obese adolescents. Atherosclerosis. 2020;315:e70.

432. Roudi R, Beikzadeh B, Roviello G, D'angelo A, Hadizadeh M: Identification of Hub Genes, Modules and Metabolic Pathways Associated With Lung Adenocarcinoma: A System Biology Approach. 2020.

433. Roussel P, Pérez O, Labbé P: Phosphate tungsten bronze series: crystallographic and structural properties of low-dimensional conductors. Acta Crystallographica Section B: Structural Science. 2001;57:603-632.

434. Rubinow KB, Henderson CM, Robinson-Cohen C, Himmelfarb J, de Boer IH, Vaisar T, Kestenbaum B, Hoofnagle AN: Kidney function is associated with an altered protein composition of high-density lipoprotein. Kidney international. 2017;92:1526-1535.

435. Rybaczyk LA: **Comparative Gene Expression Analysis To Identify Common Factors In Multiple Cancers.** The Ohio State University, 2008.

436. Saely CH, Leiherer A, Muendlein A, Vonbank A, Rein P, Geiger K, Malin C, Drexel H: High plasma omentin predicts cardiovascular events independently from the presence and extent of angiographically determined atherosclerosis. Atherosclerosis. 2016;244:38-43.

437. Sakahira H, Enari M, Nagata S: Cleavage of CAD inhibitor in CAD activation and DNA degradation during apoptosis. Nature. 1998;391:96-99.

438. Salgado-Somoza A, Teijeira-Fernández E, Fernández ÁL, González-Juanatey JR, Eiras S: Proteomic analysis of epicardial and subcutaneous adipose tissue reveals differences in proteins involved in oxidative stress. American Journal of Physiology-Heart and Circulatory Physiology. 2010;299:H202-H209.

439. Santilli F, DArdes D, Teresa Guagnano M, Davi G: Metabolic syndrome: sex-related cardiovascular risk and therapeutic approach. Current medicinal chemistry. 2017;24:2602-2627.

440. Saranya Devi K: **A Study on Visceral Adiposity Index in patients with Acute Coronary Syndrome.** Chengalpattu Medical College, Chengalpattu, 2017.

441. Satoh K, Yamada K, Maniwa T, Oda T, Matsumoto K-i: Monitoring of serial presurgical and postsurgical changes in the serum proteome in a series of patients with calcific aortic stenosis. Disease markers. 2015;2015.

442. Seres I, Lorincz H, Varga VE, Szentpeteri A, Somodi S, Harangi M, Fulop P, Paragh G: Serum afamin concentration positively correlates with the levels of pro-inflammatory adipokines in obesity. Atherosclerosis. 2017;263:e200.

443. Shao B, De Boer I, Tang C, Mayer PS, Zelnick L, Afkarian M, Heinecke JW, Himmelfarb J: A cluster of proteins implicated in kidney disease is increased in high-density lipoprotein isolated from hemodialysis subjects. Journal of proteome research. 2015;14:2792-2806.

444. Shevchenko O, Kurabekova R, Lugovskaya S, Naumova E, Gichkun O, Shevchenko A, Dolgov V: PLASMA LEVEL OF SCD40L CORRELATES WITH CIRCULATING CD34/CD45 CELL NUMBER AFTER HEART TRANSPLANTATION IN ICHEMIC PATIENTS.

445. Shim J: **Investigation of Chinese herbal medicine in treatment of metabolic syndrome.** 2008.

446. Sun Y, Johnson C, Zhou J, Wang L, Li Y-F, Lu Y, Nanayakkara G, Fu H, Shao Y, Sanchez C: Uremic toxins are conditional danger-or homeostasis-associated molecular patterns. Frontiers in bioscience (Landmark edition). 2018;23:348.

447. Suriyaprom K, Phonrat B, Namjuntra P, Harnroongroj T, Tungtrongchitr R: The—11377C> G Adiponectin Gene Polymorphism Alters the Adiponectin Concentration and the Susceptibility to Type 2 Diabetes in Thais. International Journal for Vitamin and Nutrition Research. 2010;80:216.

448. Takebayashi K, Suetsugu M, Aso Y, Inukai T: Relationship Between Circulating Aldosterone and Adipocytokines or Oxidative Stress in Patients With Type 2 Diabetes. The Endocrinologist. 2008;18:290-294.

449. Tang D, Wu Q, Yuan Z, Xu J, Zhang H, Jin Z, Zhang Q, Xu M, Wang Z, Dai Z: Identification of key pathways and genes changes in pancreatic cancer cells (BXPC-3) after cross-talk with primary pancreatic stellate cells using bioinformatics analysis. Neoplasma. 2019;66:681-693.

450. Tewari S, Renney G, Brewin J, Gardner K, Kirkham F, Inusa B, Barrett JE, Menzel S, Thein SL, Ward M, Reese DC: Proteomic analysis of plasma from children with sickle cell anemia and silent cerebral infarction. Haematologica. 2018;103:1136-1142.

451. Toneff MJ, Sreekumar A, Tinnirello A, Hollander PD, Habib S, Li S, Ellis MJ, Xin L, Mani SA, Rosen JM: The Z-cad dual fluorescent sensor detects dynamic changes between the epithelial and mesenchymal cellular states. BMC Biol. 2016;14:47.

452. Uegaki K, Otomo T, Sakahira H, Shimizu M, Yumoto N, Kyogoku Y, Nagata S, Yamazaki T: Structure of the CAD domain of caspase-activated DNase and interaction with the CAD domain of its inhibitor. J Mol Biol. 2000;297:1121-1128.

453. Vespasiani-Gentilucci U, Gallo P, De Vincentis A, Galati G, Picardi A: Hepatitis C virus and metabolic disorder interactions towards liver damage and atherosclerosis. World journal of gastroenterology: WJG. 2014;20:2825.

454. von Jeinsen B, Short MI, Xanthakis V, Carneiro H, Cheng S, Mitchell GF, Vasan RS: Association of Circulating Adipokines With Echocardiographic Measures of Cardiac Structure and Function in a Community‐Based Cohort. Journal of the American Heart Association. 2018;7:e008997.

455. Vučković V, Arizanović B, Le Blond S: Ultra-fast basic geometrical transformations on linear image data structure. Expert Systems with Applications. 2018;91:322-346.

456. Wang Y, Song W, Wang J, Wang T, Xiong X, Qi Z, Fu W, Yang X, Chen Y-G: Single-cell transcriptome analysis reveals differential nutrient absorption functions in human intestine. Journal of Experimental Medicine. 2020;217.

457. Wu D-M, Wang S, Wen X, Han X-R, Wang Y-J, Shen M, Fan S-H, Zhang Z-F, Shan Q, Li M-Q: Impact of serum omentin-1 levels on functional prognosis in nondiabetic patients with ischemic stroke. American journal of translational research. 2019;11:1854.

458. Xu K, Shuai Q, Li X, Zhang Y, Gao C, Cao L, Hu F, Akaike T, Wang JX, Gu Z, Yang J: Human VE-Cadherin Fusion Protein as an Artificial Extracellular Matrix Enhancing the Proliferation and Differentiation Functions of Endothelial Cell. Biomacromolecules. 2016;17:756-766.

459. Zhang X, Chen G-Y, Wang Z-X, Li X-H, Luo R, Li Y-G, Yang F, Zhou X, Jiang F, Wang Y-S: Nonalcoholic fatty liver disease impacts the control of the international normalized ratio in patients with atrial fibrillation. Annals of Translational Medicine. 2020;8.

460. Aballay LR, Eynard AR, Díaz MdP, Navarro A, Muñoz SE: Overweight and obesity: a review of their relationship to metabolic syndrome, cardiovascular disease, and cancer in South America. Nutrition reviews. 2013;71:168-179.

461. Abbate R, Al-Daghri NM, Andreozzi P, Borregaard N, Can G, Caridi G, Carstensen-Kirberg M, Cioni G, Conte E, Cuomo R, et al: Research update for articles published in EJCI in 2013. European Journal of Clinical Investigation. 2015;45:1005-1016.

462. ACEI I, ARBs I: RECOMMENDATIONS FOR DELAY OF DM TYPE2. Tarascon Adult Endocrinology Pocketbook. 2013;312.

463. Achour C, Aguilo F: Long non-coding RNA and Polycomb: an intricate partnership in cancer biology. Front Biosci (Landmark Ed). 2018;23:2106-2132.

464. Adamska A, Maksymowicz A, Oleksiewicz L, Otziomek E, Gorska M, Drozdowski W: Decrease of Serum Retinol-Binding Protein 4 in Ischemic Cerebral Stroke Patients With and Without Glucose Metabolism Disturbances. Diabetes. 2014;63:A569-A569.

465. Agra RM, Al‐Daghri NM, Badimon L, Bodi V, Carbone F, Chen M, Cubedo J, Dullaart RP, Eiras S, García‐Monzón C: Research update for articles published in EJCI in 2014. European journal of clinical investigation. 2016;46:880-894.

466. Alexander RW: President's address: common mechanisms of multiple diseases: why vegetables and exercise are good for you. Transactions of the American Clinical and Climatological Association. 2010;121:1.

467. Allayee H, Aouizerat B, Davis R, Drake TA, Gu J, Lusis AJ, Machleder D, Mehdizadeh S, Mehrabian M, Qiao J-H: **Genetic Factors in Atherosclerosis.** In *Vascular Endothelium.* Springer; 1998: 159-177

468. AlZaim I, Hammoud SH, Al-Koussa H, Ghazi A, Eid AH, El-Yazbi AF: Adipose tissue immunomodulation: a novel therapeutic approach in cardiovascular and metabolic diseases. Frontiers in Cardiovascular Medicine. 2020;7:277.

469. Amon TT, Loffredo TJ: **Creating Human Readable Path Constraints from Symbolic Execution.** Sandia National Lab.(SNL-NM), Albuquerque, NM (United States); 2020.

470. Anoop S, Kapoor N: **Normal-weight Obesity: A Hidden Pandemic.** In *Obesity and Diabetes.* Springer; 2020: 347-359

471. Ansaldo AM, Montecucco F, Sahebkar A, Dallegri F, Carbone F: Epicardial adipose tissue and cardiovascular diseases. International journal of cardiology. 2019;278:254-260.

472. Association AD: Acute and Chronic Complications. Diabetes. 2014;63:A561-A582.

473. Bailey SD: *Genetic Insights into Obesity and its Associated Metabolic Complications: A Multiethnic Perspective.* McGill University (Canada); 2011.

474. Balagopal P, de Ferranti SD, Cook S, Daniels SR, Gidding SS, Hayman LL, McCrindle BW, Mietus-Snyder ML, Steinberger J, Council Nutr Phys Act M, Council Epidemiology P: Nontraditional Risk Factors and Biomarkers for Cardiovascular Disease: Mechanistic, Research, and Clinical Considerations for Youth A Scientific Statement From the American Heart Association. Circulation. 2011;123:2749-2769.

475. Barrachina MN, Calderón‐Cruz B, Fernandez‐Rocca L, García Á: Application of extracellular vesicles proteomics to cardiovascular disease: guidelines, data analysis, and future perspectives. Proteomics. 2019;19:1800247.

476. Basak T, Varshney S, Akhtar S, Sengupta S: Understanding different facets of cardiovascular diseases based on model systems to human studies: A proteomic and metabolomic perspective. Journal of proteomics. 2015;127:50-60.

477. Beach S: *Transcriptional regulation of RKIP in prostate cancer progression.* The University of Toledo; 2008.

478. Bednarska-Makaruk M: **Linking adiponectin and obesity in dementia.** In *Genetics, Neurology, Behavior, and Diet in Dementia.* Elsevier; 2020: 749-767

479. Beetham H: Mechanisms of Hereditary Diffuse Gastric Cancer Initiation. 2012.

480. Bharadwaj D, Singh A: **Genetic Basis for Increased Risk for Vascular Diseases in Diabetes.** In *Mechanisms of Vascular Defects in Diabetes Mellitus.* Springer; 2017: 27-71

481. Bhatia M, Yadava O, Mittal S: Skip the Breakfast–At your Peril! 79. Cardiology. 2019;23:77-120.

482. Bianchi VE: Weight loss is a critical factor to reduce inflammation. Clinical nutrition ESPEN. 2018;28:21-35.

483. Bianchi VE: Clinical Nutrition ESPEN. 2018.

484. Bonnet C, Gonzalez S, Roberts JS, Robertson S, Ruiz M, Zheng J, Deng SX: Human limbal epithelial stem cell regulation, bioengineering and function. Progress in Retinal and Eye Research. 2021;100956.

485. Bouch C, Cousins J: *Core Topics in Anaesthesia and Perioperative Care of the Morbidly Obese Surgical Patient.* Cambridge University Press; 2018.

486. Boucher BJ: "Inverse correlation between serum free IGF-I and IGFBP-3 levels and blood pressure in patients affected with type 1 diabetes" by Capoluongo et al. Cytokine. 2007;37:181-182.

487. Bovolini A, Garcia J, Andrade MA, Duarte JA: Metabolic Syndrome Pathophysiology and Predisposing Factors. International Journal of Sports Medicine. 2020.

488. Bremer AA, Devaraj S, Afify A, Jialal I: Adipose tissue dysregulation in patients with metabolic syndrome. The Journal of Clinical Endocrinology & Metabolism. 2011;96:E1782-E1788.

489. Bremer AA, Jialal I: Adipose tissue dysfunction in nascent metabolic syndrome. Journal of obesity. 2013;2013.

490. Briggs DB: *Biochemical characterization of adiponectin oligomerization.* The University of Arizona; 2011.

491. Brodsky JB, Lemmens HJ: *Anesthetic management of the obese surgical patient.* Cambridge University Press; 2011.

492. Bucci M: LIPOTOXICITY IN OBESITY AND CORONARY ARTERY DISEASE.

493. Buschhaus A, Reitinger P, Franke J: **Automated optimization of complex three-dimensional robot trajectories.** In *2016 2nd International Conference on Control, Automation and Robotics (ICCAR)*. IEEE; 2016: 3-10.

494. Busnelli M, Manzini S, Parolini C, Ganzetti G, Dellera F, Hilvo M, Hurme R, Janis M, Sirtori C, Laaksonen R: **Liver-specific deletion of the PPAP2B gene worsens atherosclerosis in apoE-/-mice.** In *Congresso Nazionale della Società Italiana per lo Studio dell'Arteriosclerosi*. Edimes; 2015: 78-78.

495. Camarena V, Sant DW, Huff TC, Wang G: **Transcriptomic and Proteomic Analysis of the Epicardial Adipose Tissue.** In *Epicardial Adipose Tissue.* Humana, Cham; 2020: 19-36

496. Carbone F, Mach F, Montecucco F: The role of adipocytokines in atherogenesis and atheroprogression. Current drug targets. 2015;16:295-320.

497. Carrera S: **Influence of extracellular factors on p53-mediated DNA damage responses.** University of Leicester, 2013.

498. Cengiz C, Ardicoglu Y, Bulut S, Boyacioglu S: Serum retinol-binding protein 4 in patients with nonalcoholic fatty liver disease: does it have a significant impact on pathogenesis? European journal of gastroenterology & hepatology. 2010;22:813-819.

499. Center ELYS, Hall SCYC, Club CWs, Bough PRTYG, Lecture WSWM, Bingham SCY, Eiff P, Chapman SCY, Reusch JE, Bough PRYG: Carmel, California January 27Y30, 2010. 2010.

500. Chakraborty S, Kaur S, Guha S, Batra SK: The multifaceted roles of neutrophil gelatinase associated lipocalin (NGAL) in inflammation and cancer. Biochimica et Biophysica Acta (BBA)-Reviews on Cancer. 2012;1826:129-169.

501. Chaves MT, Chaves APT: COVID-19: Major Metabolic and Immunological Relationships in Obesity. MedNEXT Journal of Medical and Health Sciences. 2021;87-93.

502. Chen C, Yin N, Yin B, Lu Q: DNA methylation in thoracic neoplasms. Cancer letters. 2011;301:7-16.

503. Chowdhury A, Sarkar J, Chakraborti T, Pramanik PK, Chakraborti S: Protective role of epigallocatechin-3-gallate in health and disease: a perspective. Biomedicine & Pharmacotherapy. 2016;78:50-59.

504. Chowdhury R, M Venkat Narayan K, Zabetian A, Raj S, Tabassum R: Genetic studies of type 2 diabetes in South Asians: a systematic overview. Current diabetes reviews. 2014;10:258-274.

505. Class I, USPC AQF: Patent application title: MARKERS FOR MATURE BETA-CELLS AND METHODS OF USING THE SAME Inventors: Douglas A. Melton (Lexington, MA, US) Douglas A. Melton (Lexington, MA, US) Sinisa Hrvatin (Cambridge, MA, US). 2014.

506. Clements SJ: **The Impact of Diet on Immunosenescence.** University of East Anglia, 2017.

507. Coimbra S, Reis F, Valente MJ, Rocha S, Catarino C, Rocha-Pereira P, Sameiro-Faria M, Bronze-da-Rocha E, Belo L, Santos-Silva A: Subpopulations of High-Density Lipoprotein: Friends or Foes in Cardiovascular Disease Risk in Chronic Kidney Disease? Biomedicines. 2021;9:554.

508. Costa A, Franco OL: Impact and influence of “omics” technology on hyper tension studies. International journal of cardiology. 2017;228:1022-1034.

509. Custodero C, Mankowski R, Lee S, Chen Z, Wu S, Manini T, Echeverri JH, Sabbà C, Beavers D, Cauley J: Evidence-based nutritional and pharmacological interventions targeting chronic low-grade inflammation in middle-age and older adults: A systematic review and meta-analysis. Ageing research reviews. 2018;46:42-59.

510. Dahl TB, Bermudez B, Ranheim T, Otterdal K, Holm S, Biessen EA, Halvorsen B, Aukrust P: Unraveling the role of nicotinamide phosphoribosyltransferase on lipids in atherosclerosis. Clinical Lipidology. 2012;7:697-707.

511. Dallegri F, Carbone F: Epicardial adipose tissue and cardiovascular diseases. 2018.

512. Davis DW, Navalta JW, McGinnis GR, Serafica R, Izuora K, Basu A: Effects of acute dietary polyphenols and post-meal physical activity on postprandial metabolism in adults with features of the metabolic syndrome. Nutrients. 2020;12:1120.

513. Daxu L: Role of Adiponectin in Preventing Chronic Rejection and the Underlying Molecular Immunoregulatory Signaling Pathway. 2011.

514. DE LA DM TDE: La Diabetes Mellitus En La Practica Clinica. pdf.

515. De Rosa S, Arcidiacono B, Chiefari E, Brunetti A, Indolfi C, Foti DP: Type 2 diabetes mellitus and cardiovascular disease: genetic and epigenetic links. Frontiers in endocrinology. 2018;9:2.

516. Decker BS: **Global Genome and Hox Cluster Conformational Programming during ESC Neuronal Development: The Role of nFGFR1 and CTCF.** State University of New York at Buffalo, 2020.

517. Devaraj S, Siegel D, Jialal I: Inflammation and metabolic syndrome. The metabolic syndrome. 2011;13:210-228.

518. Dimitry S, Idriss N, Ahmed A, Abdel Aal E: Circulating Retinol Binding Protein-4 and Total Thiols In Generalized and Abdominal Obesity Regarding; Monitors Of Cardiovascular Disease. Bulletin of Egyptian Society for Physiological Sciences. 2013;33:93-106.

519. Echavarría-Pinto M, Lorenzo Hernando FA: From the epicardial adipose tissue to vulnerable coronary plaques. World journal of cardiology. 2013;5:68.

520. Eckel J: *The Cellular Secretome and Organ Crosstalk.* Academic Press; 2018.

521. Editors: Select Articles Published on the Topic of Coronary Heart Disease in 2013. Circulation. 2014;129:e432-e441.

522. Edsberg LE: **Wound Status Early Outcome Sensor and 3D Construct Development.** DAEMEN COLL AMHERST NY; 2015.

523. Esteve E, Ricart W, Fernández-Real JM: Adipocytokines and insulin resistance: the possible role of lipocalin-2, retinol binding protein-4, and adiponectin. Diabetes care. 2009;32:S362-S367.

524. Excellence WKM: AppLE AcAdEmIc pREss.

525. Fang XP: *Effects of Adiponectin on Skeletal and Cardiac Muscle Cell Metabolism.* York University; 2009.

526. Farkhondeh T, Llorens S, Pourbagher-Shahri AM, Ashrafizadeh M, Talebi M, Shakibaei M, Samarghandian S: An Overview of the Role of Adipokines in Cardiometabolic Diseases. Molecules. 2020;25.

527. Farquhar I, Kane M, Sorkin A, Summers KH: **Innovation in health, environmental and safety research infrastructure.** In *The Value of Innovation: Impact on Health, Life Quality, Safety, and Regulatory Research.* Emerald Group Publishing Limited; 2007

528. Feng L: *Regulation of retinoic acid in early zebrafish development.* University of Washington; 2009.

529. Fertuck KC: *In vitro and in vivo evaluation of the potential estrogenic effects of polycyclic aromatic hydrocarbons.* Michigan State University; 2003.

530. Fisman EZ, Tenenbaum A: Adiponectin: a manifold therapeutic target for metabolic syndrome, diabetes, and coronary disease? Cardiovascular diabetology. 2014;13:1-10.

531. Fitzgibbons TP: Role of Perivascular and Visceral Adipose Tissues in Murine Models of Obesity and Atherosclerosis: A Dissertation. 2012.

532. Fitzgibbons TP, Lee N, Tran K, Nicoloro S, Kelly M, Tam S, Czech M: Let us know how access to this document benefits you. 2016.

533. Frankel C, Watchie J: Cardiopulmonary implications of specific diseases. Essentials of Cardiopulmonary Physical Therapy-E-Book. 2016;214.

534. Franksb TMBS: Genetics of polycystic ovary syndrome. Polycystic Ovary Syndrome: Novel Insights Into Causes and Therapy. 2013;40.

535. Funnell MM, Anderson RM: 30 Influencing Self-Management: From Compliance. Type 2 Diabetes Mellitus:: An Evidence-Based Approach to Practical Management. 2008;455.

536. Gallagher EJ, LeRoith D, Karnieli E: The metabolic syndrome—from insulin resistance to obesity and diabetes. Endocrinology and metabolism clinics of North America. 2008;37:559-579.

537. Gamberi C, Johnstone O, Lasko P: Drosophila RNA binding proteins. International review of cytology. 2006;248:43-139.

538. Gandhi H, Upaganlawar A, Balaraman R: Adipocytokines: The pied pipers. J Pharmacol Pharmacother. 2010;1:9-17.

539. Ganzetti G, Fingerle J, Adorni M, Favari E, Lorenzon P, Busnelli M, Manzini S, Dellera F, Sirtori C, Bernini F: **A single infusion of trimeric apoA-I in hypercholesterolemic rabbits stabilizes atherosclerotic plaques and increases plasma cholesterol efflux capacity.** In *Congresso Nazionale della Società Italiana per lo Studio dell'Arteriosclerosi*. Edimes; 2015: 92-93.

540. Gao W, Zhang KZ: RBP4 induces pyroptosis in cardiomyocytes via activating NLRP3/Caspase-1/GSDMD pathway in acute myocardial infarction. European Heart Journal. 2020;41:3641-3641.

541. García OP, Long KZ, Rosado JL: Impact of micronutrient deficiencies on obesity. Nutrition reviews. 2009;67:559-572.

542. García-Macedo R: The 11 in Type 2 Diabetes. The Diabetes Textbook: Clinical Principles, Patient Management and Public Health Issues. 2019;145.

543. Garza AL: Anti-obesity and anti-diabetic properties of two natural extracts rich in flavonoids (helichrysum and grapefruit): physiological and molecular mechanisms. 2015.

544. Geçene M, Tuncay F, Borman P, Yücel D, Şenes M, KaniyeYılmaz B, Franks L, Radusky R, Feig J, Fernandez P: Best Oral Presentations (OP01–OP12). Rheumatology. 2012;51:i15-i18.

545. Ghoshal K, Bhattacharyya M: Adiponectin: Probe of the molecular paradigm associating diabetes and obesity. World journal of diabetes. 2015;6:151.

546. Giammanco M, Marini HR, Pallio S, Giammanco MM, Tomasello G, Carini F, Venturella F, Leto G, La Guardia M: Adipokines in obesity and metabolic diseases. Journal of Biological Research-Bollettino della Società Italiana di Biologia Sperimentale. 2020;93.

547. Gillis J, Pavlidis P: Exceptional Edges matrices from" Guilt by Association" Is the Exception Rather Than the Rule in Gene Networks Gillis, J. and Pavlidis, P.(2012) PLoS Computational Biology, 8 (3). 2012.

548. Gonçalves CG, Glade MJ, Meguid MM: Metabolically healthy obese individuals: Key protective factors. Nutrition. 2016;32:14-20.

549. Gopinath G: **A Study on Body Fat Distribution and Cardiovascular Risk Factors.** Madras Medical College, Chennai, 2009.

550. Gordon SM: **The role of high density lipoprotein compositional and functional heterogeneity in metabolic disease.** University of Cincinnati, 2012.

551. Green GC, Cuhadar A, deKemp RA: **Spatially adaptive wavelet thresholding of rubidium-82 cardiac PET images.** In *The 26th Annual International Conference of the IEEE Engineering in Medicine and Biology Society*. IEEE; 2004: 1605-1608.

552. Guerrero-Torres JC: *Tolerances allocation in modular robots interfaces design using finite elements method and the spring method.* The University of Texas at Austin; 1998.

553. Gugel I: **cDNA Microarray Analyse und Genexpressionprofil von sporadischen Vestibularisschwannomen verglichen mit gesundem Autopsiegewebe: Eine Betrachtungsweise auf molekularer Ebene unter Verwendung der Ingenuity Pathway Analysis Software.** Universität Tübingen, 2013.

554. Guglielmi V, Morretti T, Morazzini M, Sbraccia P: Fat and Lipid Partitioning: Phenotyping Beyond BMI.

555. Guglielmi V, Sbraccia P: Epicardial adipose tissue: at the heart of the obesity complications. Acta diabetologica. 2017;54:805-812.

556. Harwood HJ, Jr.: The adipocyte as an endocrine organ in the regulation of metabolic homeostasis. Neuropharmacology. 2012;63:57-75.

557. Hasanzad M, Sarhangi N, Meybodi HRA, Nikfar S, Khatami F, Larijani B: Precision medicine in non communicable diseases. International journal of molecular and cellular medicine. 2019;8:1.

558. Heinecke JW: The HDL proteome: a marker–and perhaps mediator–of coronary artery disease. Journal of lipid research. 2009;50:S167-S171.

559. Heinecke JW: The protein cargo of HDL: implications for vascular wall biology and therapeutics. Journal of clinical lipidology. 2010;4:371-375.

560. Hilton UC: 14th Annual World Congress on Insulin Resistance, Diabetes & Cardiovascular Disease (WCIRDC). Endocrine Practice. 2017;23:1A.

561. Hilton UC: 16TH Annual World Congress on Insulin Resistance, Diabetes & Cardiovascular Disease (WCIRDC). Endocrine Practice. 2019;25:1A.

562. Hirschfeld J, Chapple IL: *Periodontitis and systemic diseases: clinical evidence and biological plausibility.* Quintessenz Verlag; 2021.

563. Hosseini B, Saedisomeolia A, Skilton MR: Association between micronutrients intake/status and carotid intima media thickness: a systematic review. Journal of the Academy of Nutrition and Dietetics. 2017;117:69-82.

564. Hotel RH, Hollywood C: 9th Annual World Congress on Insulin Resistance, Diabetes & Cardiovascular Disease (WCIRDC). Endocrine Practice. 2011;17:1A.

565. Hou X, Li G, Zhao Q, Chen X, Wang C, Shi J: [Association between retinol-binding protein 4 and coronary artery disease in Chinese: a Meta-analysis]. Zhonghua Liu Xing Bing Xue Za Zhi. 2015;36:1010-1014.

566. Huang T-L, Lo L-H, Lin C-C, Hung Y-Y: Serum Proteome Analysis of Catatonia. Neuropsychiatry. 2017;7:942-951.

567. Huang Y, Yan Y, Xv W, Qian G, Li C, Zou H, Li Y: A new insight into the roles of MiRNAs in metabolic syndrome. BioMed research international. 2018;2018.

568. Iacobellis G, Barbaro G: Epicardial adipose tissue feeding and overfeeding the heart. Nutrition. 2019;59:1-6.

569. Iacobellis G, Bianco AC: Epicardial adipose tissue: emerging physiological, pathophysiological and clinical features. Trends in Endocrinology & Metabolism. 2011;22:450-457.

570. Iacobellis G, Malavazos AE, Corsi MM: Epicardial fat: from the biomolecular aspects to the clinical practice. The international journal of biochemistry & cell biology. 2011;43:1651-1654.

571. Iannucci C, Capoccia D, Calabria M, Leonetti F: Metabolic syndrome and adipose tissue: new clinical aspects and therapeutic targets. Current pharmaceutical design. 2007;13:2148-2168.

572. Ikmal SIQS, Huri HZ, Vethakkan SR, Ahmad WAW: Potential Biomarkers of Insulin Resistance and Atherosclerosis in Type 2 Diabetes Mellitus Patients with Coronary Artery Disease. International Journal of Endocrinology. 2013;2013.

573. Infante T, Forte E, Schiano C, Cavaliere C, Tedeschi C, Soricelli A, Salvatore M, Napoli C: An integrated approach to coronary heart disease diagnosis and clinical management. American journal of translational research. 2017;9:3148.

574. Itoh N, Ohta H, Konishi M: Endocrine FGFs: evolution, physiology, pathophysiology, and pharmacotherapy. Frontiers in endocrinology. 2015;6:154.

575. Jahan S: NUTRACEUTICALS AND FUNCTIONAL FOODS: THE FUTURE DIETARY APPROACH. TRENDS IN BIOCHEMISTRY AND MOLECULAR BIOLOGY. 71.

576. Jain KK: **Biomarkers of Cancer.** In *The Handbook of Biomarkers.* Springer; 2017: 273-462

577. Jardine J: *Characterising the function of the mitochondrial deubiquitylase USP30 in mitophagy.* The University of Liverpool (United Kingdom); 2021.

578. Johns KW: **Cardiovascular risk in HIV-positive patients: assessment and pharmacological treatment.** University of British Columbia, 2011.

579. Kadir RRA, Alwjwaj M, Bayraktutan U: MicroRNA: An Emerging Predictive, Diagnostic, Prognostic and Therapeutic Strategy in Ischaemic Stroke. Cellular and Molecular Neurobiology. 2020;1-19.

580. Kanagarajan S, Dhamodharan P, Mutharasappan N, Choubey SK, Jayaprakash P, Biswal J, Jeyaraman J: Structural insights on binding mechanism of CAD complexes (CPSase, ATCase and DHOase). J Biomol Struct Dyn. 2021;39:3144-3157.

581. Karam R: 1Columbia University, College of Physicians and Surgeons, New York, 2Hofstra North Shore School of Medicine/North Shore University Hospital, Forest Hills, NY, USA. 2013.

582. Kase N: The polycystic ovary syndrome–challenges and opportunities in adolescent medicine. Pediatric, adolescent, & young adult gynecology Oxford: Blackwell. 2009;316-339.

583. Kase NG: Chronic anovulation and the polycystic. Altchek's Diagnosis and Management of Ovarian Disorders. 2013;151.

584. Katsareli EA, Dedoussis GV: Biomarkers in the field of obesity and its related comorbidities. Expert opinion on therapeutic targets. 2014;18:385-401.

585. Katsi V, Vamvakou G, Lekakis J, Tousoulis D, Stefanadis C, Makris T, Kallikazaros I: Omentin, fat and heart: classical music with new instruments. Heart, Lung and Circulation. 2014;23:802-806.

586. Katsiki N, Mikhailidis DP, Wierzbicki AS: Epicardial fat and vascular risk: a narrative review. Current Opinion in Cardiology. 2013;28:458-463.

587. Katsiki N, Nikolic D, Montalto G, Banach M, Mikhailidis DP, Rizzo M: The role of fibrate treatment in dyslipidemia: an overview. Current pharmaceutical design. 2013;19:3124-3131.

588. Kaur KK, Std MSMD: Role of Adipocyte Impairment in Heart Failure Induction in Subjects that are Obese along with Prediabetes and Overt Diabetes Mellitus-A Systematic.

589. Kekis M: **Regulation of mRNA decay by Pumilio in Drosophila melanogaster.** 2013.

590. KHAING NEE: **EFFECTS OF PHYSICAL ACTIVITY AND SEDENTARY BEHAVIOUR ON HEALTH.** 2015.

591. Kim TH: *Investigating Distributed Brain Circuits In Vivo with Multi-axis Calcium Imaging.* Stanford University; 2019.

592. King RJ, Ajjan RA: Vascular risk in obesity: facts, misconceptions and the unknown. Diabetes and Vascular Disease Research. 2017;14:2-13.

593. Kohlstedt K, Gershome C, Fichtlscherer S, Busse R, Fleming I: Angiotensin converting enzyme (ACE) inhibitors modulate gene expression in human endothelial cells and adipocytes via the ACE-Dependent signaling. Circulation. 2006;114:119-119.

594. Kowalska I: Role of adipose tissue in the development of vascular complications in type 2 diabetes mellitus. Diabetes Research and Clinical Practice. 2007;78:S14-S22.

595. Kralisch S, Fasshauer M: Adipocyte fatty acid binding protein: a novel adipokine involved in the pathogenesis of metabolic and vascular disease? Diabetologia. 2013;56:10-21.

596. Kraus BJ, Sartoretto JL, Polak P, Hosooka T, Shiroto T, Eskurza I, Lee S-A, Jiang H, Michel T, Kahn BB: Novel role for retinol-binding protein 4 in the regulation of blood pressure. Faseb Journal. 2015;29:3133-3140.

597. Kumar A, Kumar A: *Diabetes: Epidemiology, Pathophysiology and Clinical Management.* CRC Press; 2020.

598. Kursawe R, Santoro N: Metabolic syndrome in pediatrics. Advances in clinical chemistry. 2014;65:91-142.

599. Kyrou I, Mattu H, Chatha K, Randeva H: **Fat hormones, adipokines.** In *Endocrinology of the Heart in health and disease.* Elsevier; 2017: 167-205

600. Lazzarini N: **Knowledge extraction from biomedical data using machine learning.** Newcastle University, 2017.

601. Le Jemtel TH, Samson R, Milligan G, Jaiswal A, Oparil S: Visceral adipose tissue accumulation and residual cardiovascular risk. Current hypertension reports. 2018;20:1-14.

602. Li M: UNIVERSITY OF PENNSYLVANIA-PERELMAN SCHOOL OF MEDICINE. 2014.

603. Li Y: *Regulation of TGFβ Signaling by microRNAs.* Rutgers The State University of New Jersey-New Brunswick and University of …; 2010.

604. Li Y: **Metabolic Plasticity in the Cellular Stress Response.** East Tennessee State University, 2018.

605. Li Y, Wright GL, Peterson JM: C1q/TNF-related protein 3 (CTRP3) function and regulation. Comprehensive Physiology. 2017;7:863.

606. Liberale L, Bonaventura A, Vecchiè A, Matteo C, Dallegri F, Montecucco F, Carbone F: The role of adipocytokines in coronary atherosclerosis. Current atherosclerosis reports. 2017;19:10.

607. Lim S, Hivert M-F: Update on the Role of Adipokines in Atherosclerosis and Cardiovascular Diseases. Current Cardiovascular Risk Reports. 2012;6:53-61.

608. Lips P: INVITED AND CONTRIBUTED PRESENTATIONS.

609. Liu L, Li HX, Lv XQ: The mechanism and significance of E-cadherin, anti-apoptosis B-cell lymphoma-2 protein and sE-cadherin roles in cancer. J Biol Regul Homeost Agents. 2014;28:683-691.

610. Lu L, Zhang RY, Wang XQ, Liu ZH, Shen Y, Ding FH, Meng H, Wang LJ, Yan XX, Yang K, et al: C1q/TNF-related protein-1: an adipokine marking and promoting atherosclerosis. Eur Heart J. 2016;37:1762-1771.

611. Lynch IV TL: **The amino terminal region of cardiac myosin binding protein-C is necessary for cardiac function.** Loyola University Chicago, 2016.

612. Macedo RG: **The Immune System and Inflammation in Type 2 Diabetes.** In *The Diabetes Textbook.* Springer; 2019: 145-167

613. Maghbooli Z, Hossein-Nezhad A: Transcriptome and molecular endocrinology aspects of epicardial adipose tissue in cardiovascular diseases: a systematic review and meta-analysis of observational studies. BioMed research international. 2015;2015.

614. Maresca F, Palma VD, Bevilacqua M, Uccello G, Taglialatela V, Giaquinto A, Esposito G, Trimarco B, Cirillo P: Adipokines, vascular wall, and cardiovascular disease: a focused overview of the role of adipokines in the pathophysiology of cardiovascular disease. Angiology. 2015;66:8-24.

615. Maria Agra R, Al-Daghri NM, Badimon L, Bodi V, Carbone F, Chen M, Cubedo J, Dullaart RPF, Eiras S, Garcia-Monzon C, et al: Research update for articles published in EJCI in 2014. European Journal of Clinical Investigation. 2016;46:880-894.

616. Matloch Z, Cinkajzlova A, Mraz M, Haluzik M: The role of inflammation in epicardial adipose tissue in heart diseases. Current pharmaceutical design. 2018;24:297-309.

617. Mayanagi T, Sobue K: Diversification of caldesmon-linked actin cytoskeleton in cell motility. Cell Adh Migr. 2011;5:150-159.

618. Mazurkiewicz J, Simiczyjew A, Dratkiewicz E, Ziętek M, Matkowski R, Nowak D: Stromal cells present in the melanoma niche affect tumor invasiveness and its resistance to therapy. International Journal of Molecular Sciences. 2021;22:529.

619. Mecacci F, Ottanelli S, Petraglia F: Mothers with HIP–The short term and long-term impact, what is new? Diabetes research and clinical practice. 2018;145:146-154.

620. Meex RCR, Watt MJ: Hepatokines: linking nonalcoholic fatty liver disease and insulin resistance. Nature Reviews Endocrinology. 2017;13:508-520.

621. Mehta S, Finkelstein J: *Nutrition and HIV: Epidemiological Evidence to Public Health.* Crc Press; 2018.

622. Mey J: RAR/RXR‐mediated signaling. Gene regulation, epigenetics, and hormone signaling. 2017;457-512.

623. Michener RD: Automating Exams for a Statistics Course: II. A Case Study. 1978.

624. Miller AP, Coronel J, Amengual J: The role of beta-carotene and vitamin A in atherogenesis: Evidences from preclinical and clinical studies. Biochimica Et Biophysica Acta-Molecular and Cell Biology of Lipids. 2020;1865.

625. Min L, Enyuan Z, Xu Z, Guangping L: Mechanism by which statins influence insulin signaling pathway. Chinese medical journal. 2014;127:3664-3668.

626. Misra S, Kumar A, Kumar P, Yadav AK, Mohania D, Pandit AK, Prasad K, Vibha D: Blood-based protein biomarkers for stroke differentiation: A systematic review. Proteomics Clinical Applications. 2017;11.

627. Mlodzik M, Gehring WJ: Expression of the caudal gene in the germ line of Drosophila: formation of an RNA and protein gradient during early embryogenesis. Cell. 1987;48:465-478.

628. Mook CK: Coronary Artery Disease and Retinol Binding Protein 4. Diabetes and Metabolism Journal. 2009;33:91-93.

629. Moschen AR, Adolph TE, Gerner RR, Wieser V, Tilg H: Lipocalin-2: a master mediator of intestinal and metabolic inflammation. Trends in Endocrinology & Metabolism. 2017;28:388-397.

630. Moulder R, Bhosale SD, Goodlett DR, Lahesmaa R: Analysis of the plasma proteome using iTRAQ and TMT‐based Isobaric labeling. Mass spectrometry reviews. 2018;37:583-606.

631. Mousa A, Naderpoor N, Teede H, Scragg R, de Courten B: Vitamin D supplementation for improvement of chronic low-grade inflammation in patients with type 2 diabetes: a systematic review and meta-analysis of randomized controlled trials. Nutrition reviews. 2018;76:380-394.

632. Mukhopadhyay S, Mondal SA, Kumar M, Dutta D: Proinflammatory and Antiinflammatory Attributes of Fetu Iν-A: A Novel Hepatokine Modulating Cardiovascular and Glycemic Outcomes in Metabolic Syndrome. Endocrine Practice. 2014;20:1345-1351.

633. Murdolo G, Angeli F, Reboldi G, Di Giacomo L, Aita A, Bartolini C, Vedecchia P: Left ventricular hypertrophy and obesity: only a matter of fat? High Blood Pressure & Cardiovascular Prevention. 2015;22:29-41.

634. Muzurović EM, Vujošević S, Mikhailidis DP: Can We Decrease Epicardial and Pericardial Fat in Patients With Diabetes? Journal of Cardiovascular Pharmacology and Therapeutics. 2021;10742484211006997.

635. Nagata S: Apoptotic DNA fragmentation. Exp Cell Res. 2000;256:12-18.

636. Nascimento IBd, Sales WB, Fleig R, Silva GDd, Silva JC: Excess weight and dyslipidemia and their complications during pregnancy: a systematic review. Revista Brasileira de Saúde Materno Infantil. 2016;16:93-101.

637. Nasreddine G, El Hajj J, Ghassibe-Sabbagh M: Orofacial clefts embryology, classification, epidemiology, and genetics. Mutation Research/Reviews in Mutation Research. 2021;108373.

638. Nesterova G, Gahl WA: Cystinosis: the evolution of a treatable disease. Pediatric Nephrology. 2013;28:51-59.

639. Neyestani T: Immune Alterations in Metabolic Syndrome: The Old Story of Chicken and Egg. Bioactive Food as Dietary Interventions for Arthritis and Related Inflammatory Diseases: Bioactive Food in Chronic Disease States. 2012;431.

640. Ng TWK: *Studies on Lipoprotein Kinetics in Obesity and the Metabolic Syndrome: Impact of Dietary Weight Loss and Statin Therapy.* University of Western Australia; 2007.

641. Nicoll R, Howard JM, Henein MY: A review of the effect of diet on cardiovascular calcification. International journal of molecular sciences. 2015;16:8861-8883.

642. Nikolaou S, Qiu S, Fiorentino F, Rasheed S, Tekkis P, Kontovounisios C: Systematic review of blood diagnostic markers in colorectal cancer. Techniques in coloproctology. 2018;22:481-498.

643. Niswender KD: Basal insulin: beyond glycemia. Postgraduate medicine. 2011;123:27-37.

644. O’Gorman PA: **Exercise Therapy as a Treatment for Chronic Liver Disease.** Trinity College, 2020.

645. Oikonomou EK, Antoniades C: Immunometabolic regulation of vascular redox state: the role of adipose tissue. Antioxidants & redox signaling. 2018;29:313-336.

646. Oikonomou EK, Antoniades C: The role of adipose tissue in cardiovascular health and disease. Nature Reviews Cardiology. 2019;16:83-99.

647. Okin DA: *Investigations into the Role of Inflammation in the Regulation of Glucose Homeostasis.* Yale University; 2015.

648. Olechnovič K, Kulberkytė E, Venclovas C: CAD-score: a new contact area difference-based function for evaluation of protein structural models. Proteins. 2013;81:149-162.

649. Olofsson L: *Molecular mechanisms in obesity-associated metabolic disease.* Inst of Medicine. Dept of Molecular and Clinical Medicine; 2007.

650. Olsen T: Lipids, Homocysteine and Vitamin A: Perspectives and new hypotheses from patients with cardiovascular disease. 2020.

651. Olsen T, Blomhoff R: Retinol, retinoic acid, and retinol-binding protein 4 are differentially associated with cardiovascular disease, type 2 diabetes, and obesity: an overview of human studies. Advances in Nutrition. 2020;11:644-666.

652. Papaetis GS, Papakyriakou P, Panagiotou TN: Central obesity, type 2 diabetes and insulin: exploring a pathway full of thorns. Archives of medical science: AMS. 2015;11:463.

653. Pennathur S, Heinecke JW: Mechanisms for oxidative stress in diabetic cardiovascular disease. Antioxidants & redox signaling. 2007;9:955-969.

654. Peter K, de Groot Gerrit RA, Stephan R, Soren T, Paul V, Andrew B, Rob H, Roger H, Richard P: ORAL SESSIONS: TRACK 3-DISEASE AND TREATMENT. International Journal of Obesity. 2007;31:S27-S36.

655. Pikir BS: *Hipertensi Manajemen Komprehensif.* Airlangga University Press; 2015.

656. Plane C, Plane S: A. 1 Anatomy Terminology.

657. Polyzos SA, Kountouras J, Zavos C: Nonalcoholic fatty liver disease: the pathogenetic roles of insulin resistance and adipocytokines. Current molecular medicine. 2009;9:299-314.

658. Pop-Busui R, Mehta M, Pennathur S: Oxidative stress and cardiovascular disease in diabetes. Studies in diabetes. 2014;189-235.

659. Porcari A, Merlo M, Rapezzi C, Sinagra G: Transthyretin amyloid cardiomyopathy: An uncharted territory awaiting discovery. European Journal of Internal Medicine. 2020.

660. Praet SF, Van Loon LJ: Exercise Therapy for Obesity and Type 2 Diabetes. Exercise Physiology: from a Cellular to an Integrative Approach. 2010;491-520.

661. Putta S, Yarla NS, Peluso I, Tiwari DK, Reddy GV, Giri PV, Kumar N, Malla R, Rachel V, Bramhachari PV: Anthocyanins: Multi-target agents for prevention and therapy of chronic diseases. Current pharmaceutical design. 2017;23:6321-6346.

662. Quinchia J, Echeverri D, Cruz-Pacheco AF, Maldonado ME, Orozco J: Electrochemical biosensors for determination of colorectal tumor biomarkers. Micromachines. 2020;11:411.

663. Ragino YI, Stakhneva EM, Polonskaya YV, Kashtanova EV: The Role of Secretory Activity Molecules of Visceral Adipocytes in Abdominal Obesity in the Development of Cardiovascular Disease: A Review. Biomolecules. 2020;10.

664. Rahman F, Fontés M: Ascorbic Acid Binding Proteins and Pathophysiology. Vitamin-Binding Proteins: Functional Consequences. 2013;257.

665. Raj M, HRM M: Cardiovascular health in children and adolescents. history. 2014;5:8.

666. Ramezani A: The effects of vitamin “D” on protein adiponectin and inflammation: The narrative review. Clinical Excellence. 2016;5:50-64.

667. Rao DP, Rao VA: Morbidly obese parturient: Challenges for the anaesthesiologist, including managing the difficult airway in obstetrics. What is new? Indian journal of anaesthesia. 2010;54:508.

668. Rasouli N, Kern PA: Adipocytokines and the metabolic complications of obesity. The Journal of Clinical Endocrinology & Metabolism. 2008;93:s64-s73.

669. Recinella L, Orlando G, Ferrante C, Chiavaroli A, Brunetti L, Leone S: Adipokines: New Potential Therapeutic Target for Obesity and Metabolic, Rheumatic, and Cardiovascular Diseases. Frontiers in Physiology. 2020;11.

670. Reddy P, Lent-Schochet D, Ramakrishnan N, McLaughlin M, Jialal I: Metabolic syndrome is an inflammatory disorder: A conspiracy between adipose tissue and phagocytes. Clinica Chimica Acta. 2019;496:35-44.

671. Richard AJ, Stephens JM: **Adipocyte-Derived Hormones.** In *Hormonal Signaling in Biology and Medicine.* Elsevier; 2020: 461-486

672. Richard AJ, White U, Elks CM, Stephens JM: Adipose tissue: physiology to metabolic dysfunction. Endotext [Internet]. 2020.

673. Robberecht H, Hermans N: Biomarkers of metabolic syndrome: biochemical background and clinical significance. Metabolic syndrome and related disorders. 2016;14:47-93.

674. Rocha VZ, Folco EJ: Inflammatory concepts of obesity. International journal of inflammation. 2011;2011.

675. Rocha VZ, Libby P: **The Metabolic Syndrome and Atherogenesis.** In *Atlas of Atherosclerosis and Metabolic Syndrome.* Springer; 2011: 45-58

676. Rodríguez A, Frühbeck G: Peptides involved in vascular homeostasis. Peptides in Energy Balance & Obesity. 2009;229-261.

677. Rodríguez-Gutiérrez A, García-Espinosa EY, Molina-Ayala MA, Ferreira-Hermosillo A: Metabolic Syndrome: from the clinical to the molecular. Revista mexicana de endocrinología metabolismo & nutrición. 2018;5:21-32.

678. Rokling-Andersen MH: Effects of nutrients and exercise on skeletal muscle and adipose tissue. 2009.

679. Ryan C, Menter A: Psoriasis and cardiovascular disorders. Giornale Italiano di Dermatologia e Venereologia. 2012;147:179-187.

680. Rychter AM, Skrzypczak-Zielinska M, Zielińska A, Eder P, Souto EB, Zawada A, Ratajczak AE, Dobrowolska A, Krela-Kaźmierczak I: Topic review Retinol-binding protein 4 in obesity Subjects: Immunology View times: 175.

681. Rychter AM, Skrzypczak-Zielińska M, Zielińska A, Eder P, Souto EB, Zawada A, Ratajczak AE, Dobrowolska A, Krela-Kaźmierczak I: Is the retinol-binding protein 4 a possible risk factor for cardiovascular diseases in obesity? International Journal of Molecular Sciences. 2020;21:1-20.

682. Rychter AM, Skrzypczak-Zielińska M, Zielińska A, Eder P, Souto EB, Zawada A, Ratajczak AE, Dobrowolska A, Krela-Kaźmierczak I: Is the Retinol-Binding Protein 4 a Possible Risk Factor for Cardiovascular Diseases in Obesity? Int J Mol Sci. 2020;21.

683. S Papaetis G, Orphanidou D, N Panagiotou T: Thiazolidinediones and type 2 diabetes: from cellular targets to cardiovascular benefit. Current drug targets. 2011;12:1498-1512.

684. Saad B, Zaid H, Shanak S, Kadan S: **Prevention and treatment of obesity-related diseases by diet and medicinal plants.** In *Anti-diabetes and Anti-obesity Medicinal Plants and Phytochemicals.* Springer; 2017: 95-128

685. Samardzija M, Neuhauss SC, Joly S, Kurz-Levin M, Grimm C: Animal models for retinal degeneration. Animal Models for Retinal Diseases. 2010;51-79.

686. Sanches JM: Biographies of the Editors. Multi-Modality Atherosclerosis Imaging and Diagnosis. 2013;409.

687. Santulli G: *Cardiovascular Disease and Diabetes: A Journey from Bench to Bedside.* Frontiers Media SA; 2019.

688. Saravanan B: **Plasma Omentin 1: A Novel Biomarker For Metabolic Risk Factors in Obesity.** KAP Viswanatham Government Medical College, Tiruchirappalli, 2017.

689. Sarvottam K, Yadav RK: Obesity-related inflammation & cardiovascular disease: Efficacy of a yoga-based lifestyle intervention. Indian Journal of Medical Research. 2014;139:822-834.

690. Satarug S, Phelps KR: **Cadmium Exposure and Toxicity.** In *Metal Toxicology Handbook.* CRC Press; 2020: 219-272

691. Satish M, Saxena SK, Agrawal DK: Adipokine Dysregulation and Insulin Resistance with Atherosclerotic Vascular Disease: Metabolic Syndrome or Independent Sequelae? Journal of Cardiovascular Translational Research. 2019;12:415-424.

692. Satoh K, Shimokawa H: Recent advances in the development of cardiovascular biomarkers. Arteriosclerosis, thrombosis, and vascular biology. 2018;38:e61-e70.

693. Scherer PE: Adipocyte-derived factors: physiological role and diagnostic use. 2008.

694. Scherer PE: The multifaceted roles of adipose tissue—therapeutic targets for diabetes and beyond: The 2015 Banting Lecture. Diabetes. 2016;65:1452-1461.

695. Schernthaner G: Pleiotropic effects of thiazolidinediones on traditional and non‐traditional atherosclerotic risk factors. International journal of clinical practice. 2009;63:912-929.

696. Schuster DP: Obesity and the development of type 2 diabetes: the effects of fatty tissue inflammation. Diabetes, metabolic syndrome and obesity: targets and therapy. 2010;3:253.

697. Seip RL: 17 Beyond Subcutaneous Fat. Obesity: Prevention and Treatment. 2012;381.

698. Sen S, Chakraborty R, De B: **Biomarkers of Diabetes and Diabetic Complications.** In *Diabetes Mellitus in 21st Century.* Springer; 2016: 101-124

699. Sen S, Chakraborty R, De B: *Diabetes mellitus in 21st century.* Springer; 2016.

700. Şengül C, Özveren O: Epicardial adipose tissue: a review of physiology, pathophysiology, and clinical applications. Anadolu Kardiyol Derg. 2013;13:261-265.

701. Shah T, Swerdlow D: Detecting, predicting and modifying cardiovascular risk: New and developing strategies. Expert Review of Cardiovascular Therapy. 2010;8:1519-1521.

702. Shams-White MM, Chung M, Du M, Fu Z, Insogna KL, Karlsen MC, LeBoff MS, Shapses SA, Sackey J, Wallace TC, Weaver CM: Dietary protein and bone health: a systematic review and meta-analysis from the National Osteoporosis Foundation. Am J Clin Nutr. 2017;105:1528-1543.

703. Shehzad A, Iqbal W, Shehzad O, Lee YS: Adiponectin: Regulation of its production and role in human diseases. Hormones. 2012;11:6-18.

704. Siegel D, Devaraj S, Mitra A, Raychaudhuri SP, Raychaudhuri SK, Jialal I: Inflammation, atherosclerosis, and psoriasis. Clinical Reviews in Allergy and Immunology. 2013;44:194-204.

705. Siest G, Nezhad MA, Bagrel D, Shamieh SE, Lambert D, Ndiaye NC, Shahabi P, Visvikis-Siest S: Functional genomics towards personalized healthcare and systems medicine. Personalized medicine. 2011;8:227-242.

706. Siitonen N: **Candidate gene studies on body size, type 2 diabetes and related metabolic traits: genetics of ADRA2B, ADIPOQ, ADIPOR1 and ADIPOR2 in the DPS study population.** Itä-Suomen yliopisto, 2011.

707. Simon TG, Corey KE, Chung RT, Giugliano R: Cardiovascular risk reduction in patients with nonalcoholic fatty liver disease: the potential role of ezetimibe. Digestive diseases and sciences. 2016;61:3425-3435.

708. Singh B, Arora S, Goswami B, Mallika V: Metabolic syndrome: A review of emerging markers and management. Diabetes and Metabolic Syndrome: Clinical Research and Reviews. 2009;3:240-254.

709. Siracusano L, Girasole V: **Sepsis and Adiponectin.** Adiponectin: Production, Regulation and Roles in Disease. Nova Publishers At ….

710. Siracusano L, Girasole V: THE ROLE OF SEPSIS AND ADIPOSE TISSUE DYSFUNCTION IN SEPSIS. International Journal of Medical and Biological Frontiers. 2012;18:435.

711. Siracuse JJ, Chaikof EL: **The pathogenesis of diabetic atherosclerosis.** In *Diabetes and peripheral vascular disease.* Springer; 2012: 13-26

712. Smekal A, Vaclavik J: Adipokines and cardiovascular disease: A comprehensive review. Biomedical Papers of the Medical Faculty of Palacky University in Olomouc. 2017;161.

713. Stefanska A, Bergmann K, Sypniewska G: Metabolic syndrome and menopause: pathophysiology, clinical and diagnostic significance. Advances in clinical chemistry. 2015;72:1-75.

714. Stuck BJ, Kahn BB: Retinol-binding protein 4 (RBP4): A biomarker for subclinical atherosclerosis. American Journal of Hypertension. 2009;22:948-949.

715. Taatjes DJ, Roth J: The Histochemistry and Cell Biology compendium: a review of 2012. Histochemistry and cell biology. 2013;139:815-846.

716. Tarantino G, Citro V, Capone D: Nonalcoholic fatty liver disease: a challenge from mechanisms to therapy. Journal of clinical medicine. 2020;9:15.

717. Taube A, Schlich R, Sell H, Eckardt K, Eckel J: Inflammation and metabolic dysfunction: links to cardiovascular diseases. American journal of physiology-heart and circulatory physiology. 2012;302:H2148-H2165.

718. Thiriet M: **Context of Cardiac Diseases.** In *Diseases of the Cardiac Pump.* Springer; 2015: 99-153

719. Time I-PCO: CONTINUOUS GLUCOSE MONITORING 1-OR.

720. Torres AG: The cad locus of Enterobacteriaceae: more than just lysine decarboxylation. Anaerobe. 2009;15:1-6.

721. Tratnjek L, Jeruc J, Romih R, Zupančič D: Vitamin A and Retinoids in Bladder Cancer Chemoprevention and Treatment: A Narrative Review of Current Evidence, Challenges and Future Prospects. International Journal of Molecular Sciences. 2021;22:3510.

722. Trayhurn P, Drevon CA, Eckel J: Secreted proteins from adipose tissue and skeletal muscle–adipokines, myokines and adipose/muscle cross-talk. Archives of physiology and biochemistry. 2011;117:47-56.

723. Tripathi P: PATHOPHYSIOLOGICAL MECHANISM BEHIND DIABETIC CARDIOVASCULAR DISORDERS. 2013.

724. Tso AWK, Xu A, Chow WS, Lam KSL: Adipose tissue and the metabolic syndrome: focusing on adiponectin and several novel adipokines. Biomarkers in Medicine. 2008;2:239-252.

725. Tycinska AM, Lisowska A, Musial WJ, Sobkowicz B: Apelin in acute myocardial infarction and heart failure induced by ischemia. Clinica Chimica Acta. 2012;413:406-410.

726. Universal City C: 13th Annual World Congress on Insulin Resistance, Diabetes & Cardiovascular Disease (WCIRDC). Endocrine Practice. 2016;22:1A.

727. Vaisar T: Proteomics investigations of HDL: challenges and promise. Current vascular pharmacology. 2012;10:410-421.

728. Verges B: Pathophysiology of diabetic dyslipidaemia: where are we? Diabetologia. 2015;58:886-899.

729. Verhagen S: *Local and systemic effects of visceral and perivascular adipose tissue.* Utrecht University; 2012.

730. Von Eynatten M, Humpert PM: Retinol-binding protein-4 in experimental and clinical metabolic disease. Expert review of molecular diagnostics. 2008;8:289-299.

731. von Zychlinski A, Kleffmann T: Dissecting the proteome of lipoproteins: New biomarkers for cardiovascular diseases? Translational Proteomics. 2015;7:30-39.

732. Vučević D, Radak Đ, Đorđević D, Miletić M, Jakovljević A, Jorgačević B, Vesković M: Chronic low grade inflammation in aging process as a link on a chain of obesity-related vascular disorders. Medicinska istraživanja. 2018;52:32-42.

733. Wagner MJ, Kim TH, Kadmon J, Nguyen ND, Ganguli S, Schnitzer MJ, Luo L: Shared cortex-cerebellum dynamics in the execution and learning of a motor task. Cell. 2019;177:669-682. e624.

734. Walther B, Lett AM, Bordoni A, Tomás‐Cobos L, Nieto JA, Dupont D, Danesi F, Shahar DR, Echaniz A, Re R: GutSelf: Interindividual variability in the processing of dietary compounds by the human gastrointestinal tract. Molecular nutrition & food research. 2019;63:1900677.

735. Wanders D, Plaisance EP, Judd RL: Pharmacological effects of lipid-lowering drugs on circulating adipokines. World journal of diabetes. 2010;1:116.

736. Wanders D, Plaisance EP, Judd RL: Lipid-lowering drugs and circulating adiponectin. Vitamins & Hormones. 2012;90:341-374.

737. Wang CL: Caldesmon and the regulation of cytoskeletal functions. Adv Exp Med Biol. 2008;644:250-272.

738. Wang J, Lin Z-J, Liu L, Xu H-Q, Shi Y-W, Yi Y-H, He N, Liao W-P: Epilepsy-associated genes. Seizure. 2017;44:11-20.

739. Wang J, Zhang Q, Islam S, Byrne BJ, Lawson LA, Smith BK, Frykman PK, Gangi A, Duel BP, Williams JA: Abstracts from IPEG 2013 The 22nd Annual Congress for Endosurgery in Children June 17–22, 2013 Beijing, China. Journal of Laparoendoscopic & Advanced Surgical Techniques. 2013;23:A-1-A-144.

740. Wang Y: Small lipid‐binding proteins in regulating endothelial and vascular functions: focusing on adipocyte fatty acid binding protein and lipocalin‐2. British journal of pharmacology. 2012;165:603-621.

741. Watchie J: 7 CHAPTER Cardiopulmonary Implications of Specific Diseases. Essentials of Cardiopulmonary Physical Therapy-E-Book. 2010;228.

742. Weichhaus MG: **Molecular aspects of the link between obesity, insulin resistance and breast cancer.** 2010.

743. Welsh M, Jamalpour M, Zang G, Åkerblom B: The role of the Src Homology-2 domain containing protein B (SHB) in β cells. Journal of molecular endocrinology. 2016;56:R21-R31.

744. Weschenfelder C, Schaan de Quadros A, Lorenzon dos Santos J, Bueno Garofallo S, Marcadenti A: Adipokines and adipose tissue-related metabolites, nuts and cardiovascular disease. Metabolites. 2020;10:32.

745. Westerink J, Visseren FL: Pharmacological and non-pharmacological interventions to influence adipose tissue function. Cardiovascular diabetology. 2011;10:1-12.

746. Widłak P: The DFF40/CAD endonuclease and its role in apoptosis. Acta Biochim Pol. 2000;47:1037-1044.

747. Yim J, Rabkin SW: Differences in Gene Expression and Gene Associations in Epicardial Fat Compared to Subcutaneous Fat. Horm Metab Res. 2017;49:327-337.

748. Zeman M, Vecka M, Perlík F, Staňková B, Hromádka R, Tvrzická E, Širc J, Hrib J, Žák A: Pleiotropic effects of niacin: Current possibilities for its clinical use. Acta Pharmaceutica. 2016;66:449-469.

749. Zhang M, Wang M, Tai Y, Tao J, Zhou W, Wang Q: Triggers of Cardiovascular Diseases in Rheumatoid Arthritis. Current Problems in Cardiology. 2021;100853.

750. Zhang P, Gao J, Pu C, Zhang Y: Apolipoprotein status in type 2 diabetes mellitus and its complications. Molecular medicine reports. 2017;16:9279-9286.

751. Zhang S-y, Yang K-l, Long Z-y, Li W-q, Huang H-y: Use of a systematic pharmacological methodology to explore the mechanism of shengmai powder in treating diabetic cardiomyopathy. Medical science monitor: international medical journal of experimental and clinical research. 2020;26:e919029-919021.

752. ZHOU G: **Synthesis and Evaluation of Small Molecules for Controlling Stem Cell Development.** Durham University, 2014.

753. Zhou Y, Zhang B, Hao C, Huang X, Li X, Huang Y, Luo Z: Omentin-A novel adipokine in respiratory diseases. International journal of molecular sciences. 2018;19:73.

754. Zorena K: Obesity and Type 2 Diabetes Mellitus: Adipocytokines as Markers of Insulin Resistance. Frontiers in Clinical Drug Research: Diabetes and Obesity: Volume: 3. 2016;3:195.

755. Baig M, Alghalayini KW, Gazzaz ZJ, Atta H: Association of serum omentin-1, chemerin, and leptin with acute myocardial infarction and its risk factors. Pakistan Journal of Medical Sciences. 2020;36:1183.

756. Bandara E: **Association of Risk Factors for Development of Coronary Artery Disease and Nutritional and Immune Status on the Recovery Following Coronary Artery Bypass Surgery.** University of Sri Jayewardenepura, Nugegoda, 2015.

757. Bhaskar S, Ganesan M, Chandak GR, Mani R, Idris MM, Khaja N, Gulla S, Kumar U, Movva S, Vattam KK: Association of PON 1 and APOA 5 gene polymorphisms in a cohort of Indian patients having coronary artery disease with and without type 2 diabetes. Genetic Testing and Molecular Biomarkers. 2011;15:507-512.

758. Bilbija D, Elmabsout AA, Sagave J, Haugen F, Bastani N, Dahl CP, Gullestad L, Sirsjö A, Blomhoff R, Valen G: Expression of retinoic acid target genes in coronary artery disease. International journal of molecular medicine. 2014;33:677-686.

759. CHAI R-n, LU X-f, ZHANG S-w, YAN H, LI P-f, LIU J, WANG W, LUO J, XU D-f, GAO W: Correlation of blood serum visfatin with type 2 diabetes mellitus complicated with coronary artery disease [J]. Acta Academiae Medicinae Militaris Tertiae. 2009;5.

760. Choi KM, Lee JS, Kim EJ, Baik SH, Seo HS, Choi DS, Oh DJ, Park CG: Implication of lipocalin-2 and visfatin levels in patients with coronary heart disease. European Journal of Endocrinology. 2008;158:203-207.

761. Elnajjar MM, Dawood AA, Soliman MA, Khalil GI, Elzorkany KMA, Aglan MF: Serum chemerin and its association with coronary heart disease in diabetic and nondiabetic patients. Menoufia Medical Journal. 2018;31:474.

762. Fain JN, Sacks HS, Bahouth SW, Tichansky DS, Madan AK, Cheema PS: Human epicardial adipokine messenger RNAs: comparisons of their expression in substernal, subcutaneous, and omental fat. Metabolism. 2010;59:1379-1386.

763. Fiévet C, Staels B: Efficacy of peroxisome proliferator-activated receptor agonists in diabetes and coronary artery disease. Current atherosclerosis reports. 2009;11:281-288.

764. Hanboly NH, Sharaf Y, Al-Anany M, Saeed E: Serum chemerin as a predictor of left ventricle hypertrophy in patients with coronary artery disease. Nigerian Journal of Cardiology. 2019;16:25.

765. Heng C-K, Ooi DS, Dorajoo R, Chan MY-Y, Low AF-H, Friedlander Y: Investigation of the novel androgen-dependent tissue factor pathway inhibitor regulating protein (ADTRP) and its role in coronary artery disease. Atherosclerosis. 2017;263:e199-e200.

766. Holvoet P, Klocke B, Vanhaverbeke M, Menten R, Sinnaeve P, Raitoharju E, Lehtimäki T, Oksala N, Zinser C, Janssens S, et al: RNA-sequencing reveals that STRN, ZNF484 and WNK1 add to the value of mitochondrial MT-COI and COX10 as markers of unstable coronary artery disease. PLoS One. 2019;14:e0225621.

767. Isbir T, Yilmaz H, Agachan B, Karaali ZE: Cholesterol ester transfer protein, apolipoprotein E and lipoprotein lipase genotypes in patients with coronary artery disease in the Turkish population. Clin Genet. 2003;64:228-234.

768. Jafaripour S, Sasanejad P, Dadgarmoghaddam M, Sadr-Nabavi A: ADAMTS7 and ZC3HC1 Share Genetic Predisposition to Coronary Artery Disease and Large Artery Ischemic Stroke. Crit Rev Eukaryot Gene Expr. 2019;29:351-361.

769. Kadhim DJ: **Association of Admission Serum Adiponectin, Resistin and Leptin Levels with Acute ST-Segment Elevation Myocardial Infarction.** Department of Clinical Pharmacy and the Committee of Graduate Studies of the …, 2013.

770. Kharb R, Sharma A, Chaddar MK, Yadav R, Agnihotri P, Kar A, Biswas S: Plasma proteome profiling of coronary artery disease patients: downregulation of transthyretin—an important event. Mediators of inflammation. 2020;2020.

771. Lepper PM, Schumann C, Triantafilou K, Rasche FM, Schuster T, Frank H, Schneider EM, Triantafilou M, von Eynatten M: Association of lipopolysaccharide-binding protein and coronary artery disease in men. J Am Coll Cardiol. 2007;50:25-31.

772. Manfredi M, Chiariello C, Conte E, Castagna A, Robotti E, Gosetti F, Patrone M, Martinelli N, Bassi A, Cecconi D: Plasma Proteome Profiles of Stable CAD Patients Stratified According to Total Apo C‐III Levels. PROTEOMICS–Clinical Applications. 2019;13:1800023.

773. Munjas J, Sopić M, Spasojević-Kalimanovska V, Kalimanovska-Oštrić D, Anđelković K, Jelić-Ivanović Z: Association of adenylate cyclase-associated protein 1 with coronary artery disease. Eur J Clin Invest. 2017;47:659-666.

774. Naryzhnaya NV, Koshelskaya OA, Kologrivova IV, Kharitonova OA, Evtushenko VV, Boshchenko AA: Hypertrophy and Insulin Resistance of Epicardial Adipose Tissue Adipocytes: Association with the Coronary Artery Disease Severity. Biomedicines. 2021;9:64.

775. Oh J-Y: Serum cystatin C as a biomarker for predicting coronary artery disease in diabetes. Korean diabetes journal. 2010;34:84-85.

776. Phulukdaree A, Moodley D, Khan S, Chuturgoon AA: Uncoupling protein 2 -866G/A and uncoupling protein 3 -55C/T polymorphisms in young South African Indian coronary artery disease patients. Gene. 2013;524:79-83.

777. Poduri A, Bahl A, Talwar KK, Khullar M: Proteomic analysis of circulating human monocytes in coronary artery disease. Mol Cell Biochem. 2012;360:181-188.

778. Shen L, Wang S, Ling Y, Liang W: Association of C1q/TNF-related protein-1 (CTRP1) serum levels with coronary artery disease. J Int Med Res. 2019;47:2571-2579.

779. Silaghi A, Achard V, Paulmyer-Lacroix O, Scridon T, Tassistro V, Duncea I, Clément K, Dutour A, Grino M: Expression of adrenomedullin in human epicardial adipose tissue: role of coronary status. American Journal of Physiology-Endocrinology and Metabolism. 2007;293:E1443-E1450.

780. Smékal A, Vaclavik J, Stejskal D, Benešová K, Jarkovský J, Svobodova G, Richterova R, Švesták M, Táborský M: Plasma levels and leucocyte RNA expression of adipokines in young patients with coronary artery disease, in metabolic syndrome and healthy controls. Cytokine. 2019;122:154017.

781. Sottero B, Pozzi R, Leonarduzzi G, Aroasio E, Gamba P, Gargiulo S, Rabajoli F, Ferrari F, Greco Lucchina P, Poli G: Lipid peroxidation and inflammatory molecules as markers of coronary artery disease. Redox Rep. 2007;12:81-85.

782. Syvänne M, Castro G, Dengremont C, De Geitere C, Jauhiainen M, Ehnholm C, Michelagnoli S, Franceschini G, Kahri J, Taskinen MR: Cholesterol efflux from Fu5AH hepatoma cells induced by plasma of subjects with or without coronary artery disease and non-insulin-dependent diabetes: importance of LpA-I:A-II particles and phospholipid transfer protein. Atherosclerosis. 1996;127:245-253.

783. Tan L, Xu Q, Wang Q, Shi R, Zhang G: Identification of key genes and pathways affected in epicardial adipose tissue from patients with coronary artery disease by integrated bioinformatics analysis. PeerJ. 2020;8:e8763.

784. Vaisar T, Pennathur S, Green PS, Gharib SA, Hoofnagle AN, Cheung MC, Byun J, Vuletic S, Kassim S, Singh P: Shotgun proteomics implicates protease inhibition and complement activation in the antiinflammatory properties of HDL. The Journal of clinical investigation. 2007;117:746-756.

785. Wan K, Zhao J, Deng Y, Chen X, Zhang Q, Zeng Z, Zhang L, Chen Y: A genetic polymorphism in RBP4 is associated with coronary artery disease. Int J Mol Sci. 2014;15:22309-22319.

786. Wang H, Wang X, Cao Y, Han W, Guo Y, Yang G, Zhang J, Jiang P: Association of polymorphisms of preptin, irisin and adropin genes with susceptibility to coronary artery disease and hypertension. Medicine (Baltimore). 2020;99:e19365.

787. Wang Y: **Phenotyping of circulating monocytes in coronary artery diseases.** Universität Ulm, 2015.

788. Yan L, Cao X, Zeng S, Li Z, Lian Z, Wang J, Lv F, Wang Y, Li Y: Associations of proteins relevant to MAPK signaling pathway (p38MAPK-1, HIF-1 and HO-1) with coronary lesion characteristics and prognosis of peri-menopausal women. Lipids in health and disease. 2016;15:1-11.

789. Zhang Y, Liu C, Liu J, Guo R, Yan Z, Liu W, Lau WB, Jiao X, Cao J, Xu K, et al: Implications of C1q/TNF-related protein superfamily in patients with coronary artery disease. Sci Rep. 2020;10:878.

790. Zhou X-z, Shi R, Wang J, Shi K, Liu X, Li Y, Gao Y, Guo Y-k, Yang Z-g: Characteristics of coronary artery disease in patients with subclinical hypothyroidism: evaluation using coronary artery computed tomography angiography. BMC Cardiovascular Disorders. 2021;21:1-12.

791. Cheng X, Wu Z, Yuan B: Diagnostic and predictive value of serum LDL/HDL and RBP4 levels in restenosis after revascularization in patients with coronary heart disease (CHD). International Journal of Clinical and Experimental Medicine. 2019;12:10783-10788.

792. Cosentino RG, Churilla JR, Josephson S, Molle-Rios Z, Hossain MJ, Prado W, Balagopal PB: Branched chain amino acid-inflammation relationship in youth with obesity: A randomized controlled intervention study. The Journal of Clinical Endocrinology & Metabolism. 2021.

793. Derosa G, Cicero A, D'Angelo A, Bonaventura A, Bianchi L, Romano D, Maffioli P: Effects of an olmesartan/amlodipine combination compared to olmesartan or amlodipine monotherapies on some insulin resistance parameters in hypertensive patients. European Heart Journal. 2013;34.

794. Derosa G, Cicero AFG, Fogari E, D'Angelo A, Bonaventura A, Maffioli P: Effects of n-3 PUFA on insulin resistance after an oral fat load. European Journal of Lipid Science and Technology. 2011;113:950-960.

795. Derosa G, Maffioli P, D'Angelo A, Fogari E, Bianchi L, Cicero AFG: RETRACTED: Acarbose on insulin resistance after an oral fat load: a double-blind, placebo controlled study (Retracted article. See vol. 31, pg. 1248, 2017). Journal of Diabetes and Its Complications. 2011;25:258-266.

796. Elmadhun NY, Lassaletta AD, Chu LM, Sellke FW: Metformin alters the insulin signaling pathway in ischemic cardiac tissue in a swine model of metabolic syndrome. J Thorac Cardiovasc Surg. 2013;145:258-265; discussion 265-256.

797. Fuernau G, Beck J, Desch S, Eitel I, Erbs S, Mangner N, Fengler K, Sandri M, Schuler G, Thiele H: Mild hypothermia in cardiogenic shock complicating myocardial infarction–the randomized SHOCKCOOL pilot trial. Eur Heart J. 2016;37:1041.

798. Gharipour M, Sadeghi M, Behmanesh M, Salehi M, Roohafza H, Nezafati P, Khosravi E, Hosseini M, Keshvari M, Rouhi-Bourojeni H, Sarrafzadegan N: Proposal of a study protocol of a preliminary double-blind randomized controlled trial. Verifying effects of selenium supplementation on selenoprotein p and s genes expression in protein and mRNA levels in subjects with coronary artery disease: selenegene. Acta Biomed. 2019;90:44-50.

799. Huang F, del-Río-Navarro BE, Leija-Martinez J, Torres-Alcantara S, Ruiz-Bedolla E, Hernández-Cadena L, Barraza-Villarreal A, Romero-Nava R, Sanchéz-Muñoz F, Villafaña S: Effect of omega-3 fatty acids supplementation combined with lifestyle intervention on adipokines and biomarkers of endothelial dysfunction in obese adolescents with hypertriglyceridemia. The Journal of nutritional biochemistry. 2019;64:162-169.

800. Karakas SE, Banaszewska B, Spaczynski RZ, Pawelczyk L, Duleba A: Free fatty acid binding protein-4 and retinol binding protein-4 in polycystic ovary syndrome: response to simvastatin and metformin therapies. Gynecological Endocrinology. 2013;29:483-487.

801. Liu JF, Li YS, Drew PA, Zhang C: The effect of celecoxib on DNA methylation of CDH13, TFPI2, and FSTL1 in squamous cell carcinoma of the esophagus in vivo. Anti-cancer drugs. 2016;27:848-853.

802. Moyle GJ, Stellbrink H-J, Compston J, Orkin C, Arribas JR, Domingo P, Granier C, Pearce H, Sedani S, Gartland M, Team A: 96-Week results of abacavir/lamivudine versus tenofovir/emtricitabine, plus efavirenz, in antiretroviral-naive, HIV-1-infected adults: ASSERT study. Antiviral Therapy. 2013;18:905-913.

803. Mukhtar R: **Metabolic Syndrome, Weight and Cardiovascular Co-morbidities: A Randomised Study Comparing the Effect of Three Dietary Approaches on Cardiovascular Risk in Subjects with the Metabolic Syndrome.** University of Bath, 2013.

804. Noce A, Marrone G, Di Daniele F, Di Lauro M, Pietroboni Zaitseva A, Wilson Jones G, De Lorenzo A, Di Daniele N: Potential Cardiovascular and Metabolic Beneficial Effects of ω-3 PUFA in Male Obesity Secondary Hypogonadism Syndrome. Nutrients. 2020;12:2519.

805. Oh EG, Bang SY, Kim SH, Hyun SS, Chu SH, Jeon YK, Im JA, Lee MK, Lee JE: Effect of 6-Month Therapeutic Lifestyle Modification Program in Reducing Metabolic Coronary Heart Disease Risk in Women with Metabolic Syndrome in the Community. Circulation. 2009;119:E284-E284.

806. Parás Chávez C: **The effect of n-3 PUFA on metabolic and inflammatory markers in normal weight and obese subjects and the modulation of inflammatory signals by different fatty acids in THP-1 derived macrophages.** University of Southampton, 2014.

807. Patel S, Murthy S, Bhatia V, Edwards P, Goldstein D, Maybaum S: 462 Cardiac Improvement with Combined Neurohormonal Blockade and Support with a Continuous Flow LVAD. The Journal of Heart and Lung Transplantation. 2011;30:S157-S158.

808. Post FA, Moyle GJ, Stellbrink HJ, Domingo P, Podzamczer D, Fisher M, Norden AG, Cavassini M, Rieger A, Khuong-Josses M-A, et al: Randomized Comparison of Renal Effects, Efficacy, and Safety With Once-Daily Abacavir/Lamivudine Versus Tenofovir/Emtricitabine, Administered With Efavirenz, in Antiretroviral-Naive, HIV-1-Infected Adults: 48-Week Results From the ASSERT Study. Jaids-Journal of Acquired Immune Deficiency Syndromes. 2010;55:49-57.

809. Robinson DG, Margrain TH, Dunn MJ, Bailey C, Binns AM: Low-level nighttime light therapy for age-related macular degeneration: a randomized clinical trial. Investigative ophthalmology & visual science. 2018;59:4531-4541.

810. Sakai K, Ikari Y: P6437Inpact of intravascular ultrasound guided coronary intervention using minimum dose of contrast on 1 year clinical outcomes in patients with severe chronic kidney disease-MINICON2 study. European Heart Journal. 2017;38.

811. Salgado-Somoza A, Teijeira-Fernández E, Fernández ÁL, González-Juanatey JR, Eiras S: Changes in lipid transport-involved proteins of epicardial adipose tissue associated with coronary artery disease. Atherosclerosis. 2012;224:492-499.

812. Toth PP, Bays H, Farnier M, Jensen E, Tomassini JE, Polis A, Lin J, Bird S, Foody J, Tershakovec AM: A comparison of the attainment of guideline-recommended LDL-C lowering with statin and ezetimibe+ statin therapies. Atherosclerosis. 2017;263:e240-e241.

813. Verhagen SN, Buijsrogge MP, Vink A, van Herwerden LA, van der Graaf Y, Visseren FL: Secretion of adipocytokines by perivascular adipose tissue near stenotic and non-stenotic coronary artery segments in patients undergoing CABG. Atherosclerosis. 2014;233:242-247.

814. Verhagen SN, Buijsrogge MP, Vink A, van Herwerden LA, van der Graaf Y, Visseren FL: Secretion of adipocytokines by perivascular adipose tissue near stenotic and non-stenotic coronary artery segments in patients undergoing CABG. Atherosclerosis. 2014;233:242-247.

815. You SH, Kim BS, Hong SJ, Ahn CM, Lim DS: The effects of pioglitazone in reducing atherosclerosis progression and neointima volume in type 2 diabetic patients: Prospective randomized study with volumetric intravascular ultrasonography analysis. Korean Circulation Journal. 2010;40:625-631.

816. Aquino LA, Pereira SE, Sobrinho CJS, Ramalho A: Associação entre a deficiência de vitamina A e indicadores antropométricos em indivíduos com obesidade grau III. RBONE-Revista Brasileira de Obesidade, Nutrição e Emagrecimento. 2008;2.

817. Aragonés Bargalló G: **Fabp4 i biomarcadors de la disfunció endotelial. Estudi clínic i in vitro.** Universitat Rovira i Virgili.

818. Auto C: شير ‌اَ ‌.

819. BB SD, ISOLE ADEGI, LANGERHANS D, BB IDRDS, DAI LORO O: Tesi di Laurea Sperimentale in Farmacologia e Farmacoterapia.

820. Beáta LDT: Varga Viktória Evelin A szelektív LDL aferezis non-lipid hatásai súlyos heterozigóta familiáris hiperkoleszterinémiás betegek esetén.

821. Benaiges Martinez C: *Teixit adipós epicàrdic i senyalització a través dels Receptors Toll like (TLR): Paper en la patofisiologia de l'aterosclerosi.* Universitat Autònoma de Barcelona; 2015.

822. Bispo PFF: Estratégias para as opções dietéticas de combate à obesidade relacionadas com a morbilidade cardiometabolica. 2017.

823. Bodmann KF, Schuster HP, Jürgens P, Tröster S: [Behavior of plasma proteins and nitrogen balance in patients with acute myocardial infarct with and without intravenous amino acid administration]. Infusionsther Transfusionsmed. 1993;20:76-80.

824. Buschhaus A: *Hochpräzise adaptive Steuerung und Regelung robotergeführter Prozesse.* Friedrich-Alexander-Universität Erlangen-Nürnberg (FAU); 2017.

825. Camera A, Hopps E, Caimi G: [Metabolic syndrome: from insulin resistance to adipose tissue dysfunction]. Minerva Med. 2008;99:307-321.

826. Campderrós Traver L: GDFI5, un nou factor secretable regulador del metabolisme. Paper com a" batoquina" en models experimentals, i estudis en humans. 2019.

827. Cardoso DF: Mudanças fisiológicas da diabetes e a reação do exercício físico. 2017.

828. Chan S-H: 胰島素受體受質和疾病及致病機制關聯性之探討. 成功大學臨床醫學研究所學位論文. 2013;1-113.

829. Chielle EO: **Avaliação de potenciais biomarcadores séricos e salivares e do polimorfismo do gene da glutationa S-transferase P1 (GSTP1) na obesidade adulto jovem.** Universidade Federal de Santa Maria, 2015.

830. Chu C-S: 陰電性低密度脂蛋白透過 L5/LOX-1/CRP 迴路增幅血管內皮細胞毒性及動脈粥狀硬化. 高雄醫學大學醫學研究所學位論文. 2014;1-134.

831. Comucci EB: Níveis séricos da proteína carreadora do retinol 4 e risco cardiovascular no diabetes mellitus tipo 2. 2012.

832. Cortes Ibarra MA: DIFERENCIAS GENETICAS, FENOTIPICAS Y DISTANCIAS GENETICAS PARA LOS GENES ESR, FUT1 Y RBP4 EN POBLACIONES DE RAZAS DE CERDOS YORKSHIRE, PELON MEXICANO Y CUINOS. 2007.

833. Costa MGdS: Alterações no proteoma caulinar de Eucalyptus globulus e Eucalyptus grandis em resposta a variações de temperatura. 2017.

834. Cuervo Pinto R: Evaluación de las alteraciones de la glucemia y su manejo intrahospitalario y el alta como marcador pronóstico en el servicio de urgencias y unidades vinculadas. 2017.

835. Dametto JFdS: Avaliação da suplementação pós-parto com vitamina E sobre a concentração de retinol e alfa-tocoferol no soro e leite maternos. 2018.

836. de Aquino LA, Pereira SE, Sobrinho CJS, Ramalho A: Association between vitamin A deficiency and anthropometric markers in individuals with class III obesity/Associacao entre a deficiencia de vitamina A e indicadores antropometricos em individuos com obesidade grau III. Revista Brasileira de Obesidade, Nutrição e Emagrecimento. 2008;2:404-412.

837. Delgado García AF: **Incidencia de factores de riesgo de síndrome metabólico en estudiantes de la Facultad de Ciencias Químicas de la Universidad de Guayaquil.** Universidad de Guayaquil. Facultad de Ciencias Químicas, 2014.

838. Doubková K: **Ekonomický rámec prevence a podpory zdraví u kardiovaskulárních onemocnění.** České vysoké učení technické v Praze. Vypočetní a informační centrum., 2016.

839. Duval PA, Silveira DH, Assunção MCF: Intervenção nutricional e de modificação no estilo de vida tem efeito positivo em adultos com síndrome metabólica.

840. Fornovi Justo A: El bypass gástrico como herramienta terapéutica en el paciente diabético y obeso: cambios en el riesgo cardiovascular tras la cirugía metabólica. Proyecto de investigación:. 2017.

841. Gallina F, Servillo G, Della Fazia MA: Diabete: frutto dei nostri geni o del nostro stile di vita?

842. Garbossa RA, Canale NE: CONTROLE LITO-ESTRUTURAL NA ORGANIZAÇÃO ESPACIAL DA BACIA DO RIO TAGAÇABA–PR: ANÁLISE MORFOMÉTRICA DA REDE DE DRENAGEM. 2003.

843. Gibala D: Avaliação do efeito do selênio no processo de reparação tecidual em pacientes com pé diabético. 2018.

844. Gómez JÁ: II Coloquio en Medicina Hospitalaria y de Emergencia 2014.

845. Guebre-Egziabher F: **Modulation de l’apport en acides gras polyinsaturés n-3: intérêt chez le sujet sain et au cours de l’insuffisance rénale chronique.** Université Claude Bernard-Lyon I, 2010.

846. Hamidi M: Stabil angina pektorisli hastalarda vaspin ve visfatin düzeylerinin değerlendirilmesi ve koroner arter hastalığı ciddiyeti ile ilişkisi. 2013.

847. Hernández López SH: Evaluación de genes candidatos por PCR-RFLP para características reproductivas en hembras porcinas. CONACYT. 2006.

848. Iskandar A, Mayashinta DK, Indra MR: *Mengenal Toxoplasma Gondii, Obesitas, dan Sindrom Metabolik.* Universitas Brawijaya Press; 2018.

849. Isobe MT: **Validação de um questionário de frequência alimentar para estimativa da ingestão de vitamina A em gestantes.** Universidade de São Paulo.

850. Jung REUN, Choi ES, Young PC, Lee W-Y, Sung K, 김지훈, 원종철, 오기원, 김병진, 김범수, et al: The Relationship between Serum Retinol-Binding Protein 4 Levels and Coronary Artery Disease in Korean Adults. Diabetes and Metabolism Journal. 2009;33:105-112.

851. Kim J-H, Rhee E-J, Choi E-S, Won J-C, Park C-Y, Lee W-Y, Oh K-W, Kim B-J, Sung K-C, Kim B-S: The Relationship between Serum Retinol-Binding Protein 4 Levels and Coronary Artery Disease in Korean Adults. Korean Diabetes Journal. 2009;33:105-112.

852. Klatko W, Niemczyk S: Biozgodność u chorych z przewlekłą chorobą nerek leczonych hemodializami i dializą otrzewnową. Indukacja stanu zapalnego przez zabiegi dializy Zmiany stężeń leptyny i neuropeptydu Y w próbie 4-godzinnego głodzenia Ocena wpływu stanu zapalnego na stężenia adipocytokin u pacjentów przewlekle dializowanych. 1997;100:419-424.

853. Kretzer DC: Consumo alimentar de gestantes com e sem diabetes mellitus gestacional, ganho de peso gestacional e alterações antropométricas do recém-nascido nos primeiros seis meses de vida: estudo IVAPSA. 2019.

854. Lagrange J: **Changements hémostatiques du syndrome métabolique, de l'hypertension artérielle, et de l'insuffisance cardiaque: approches physiologique et physiopathologique.** Université de Lorraine, 2013.

855. Lázaro López I: **Regulació de fabp4 depenent de nrf2 en macròfags.** Universitat Rovira i Virgili, 2010.

856. Lcdo RM, Lcda SO, Lcda ZR, Añez RJ, Joselyn Rojas M, Valmore Bermúdez M: Prevalencia de diabetes mellitus tipo 2 en individuos adultos del municipio San Cristóbal del estado Táchira, Venezuela/Type 2 diabetes mellitus prevalence in the adult population of San Cristóbal municipality from Táchira State, Venezuela. Diabetes Internacional. 2015;7:18.

857. Li F, Yang T, Zhao Z, Xia K: [Plasma level of RBP4 in patients with coronary heart disease and the effect of hyperinsulinemia]. Zhong Nan Da Xue Xue Bao Yi Xue Ban. 2012;37:1177-1182.

858. Li J, Huang X, Jiang Y, Luo F, Mao Z: [Expression and clinical significance of serum retinol binding protein 4, superoxide dismutase and hypersensitive C-reactive protein in patients with acute ST-segment elevated myocardial infarction]. Zhonghua Wei Zhong Bing Ji Jiu Yi Xue. 2020;32:1199-1202.

859. Liu TF, Lin T, Ren LH, Li GP, Peng JJ: [Association between CMTM5 gene and coronary artery disease and the relative mechanism]. Beijing Da Xue Xue Bao Yi Xue Ban. 2020;52:1082-1087.

860. Luo T, Yan A, Liu L, Jiang H, Feng C, Liu G, Liu F, Tang D, Zhou T: [In vitro study of joint intervention of E-cad and Bmi-1 mediated by transcription activator-like effector nuclease in nasopharyngeal carcinoma]. Zhong Nan Da Xue Xue Bao Yi Xue Ban. 2018;43:229-239.

861. Martos Moreno GÁ: El adipocito como órgano endocrino: adipokinas durante el desarrollo e implicaciones clínicas en la obesidad en la infancia al diagnóstico y tras reducción ponderal. 2008.

862. Memiç K: Koroner arter bypass cerrahisi yapılan hastalarda kardiyovasküler risk faktörleri ve koroner ateroskleroz ciddiyetinin uzun dönem greft açıklık oranına etkisi. 2011.

863. Nagajyothi F, Desruisseaux MS, Weiss LM, Chua S, Albanese C, Machado FS, Esper L, Lisanti MP, Teixeira MM, Scherer PE: Chagas disease, adipose tissue and the metabolic syndrome. Memórias do Instituto Oswaldo Cruz. 2009;104:219-225.

864. Nascimento IBd, Sales WB, Fleig R, Silva GDd, Silva JC: Excesso de peso e dislipidemia e suas intercorrências no período gestacional: uma revisão sistemática. Revista Brasileira de Saúde Materno Infantil. 2016;16:93-101.

865. Nunes IJG: Gene expression analysis platform (GEAP): uma plataforma flexível e intuitiva para análise de transcriptoma. 2018.

866. Oliveira RB: Avaliação da expressão hepática de microRNAs relacionados ao metabolismo lipídico na prole de camundongos com obesidade induzida por dieta. 2013.

867. Pinto RC: *Evaluación de las alteraciones de la glucemia y su manejo intrahospitalario y el alta como marcador pronóstico en el servicio de urgencias y unidades vinculadas.* Universidad Complutense de Madrid; 2016.

868. Posada Ayala M: Patología cardiovascular y enfermedad renal: búsqueda de biomarcadores de riesgo y diagnóstico temprano a nivel proteómico y metabólico. 2014.

869. PRATA MF: EVIDÊNCIAS DE EFEITO HIPOLIPIDÊMICO DO EXTRATO HIDROALCOÓLICO DE PRÓPOLIS VERMELHA EM RATOS. 2020.

870. Qaddoura NM: **جزيئات الحامض النووي الريبوزي الصغيرة كعلامة حيوية لمرضى السكري من النوع الثاني في قطاع غزة.** الجامعة الإسلامية بغزة, 2018.

871. Qi RX, Xu XY: [Inverse correlation of S100A4 and E-cad protein expression and their clinical significance in non-small cell lung cancer]. Zhonghua Zhong Liu Za Zhi. 2007;29:681-684.

872. Rensing L, Rippe V: *Altern: zelluläre und molekulare Grundlagen, körperliche Veränderungen und Erkrankungen, Therapieansätze.* Springer-Verlag; 2013.

873. Resende FBS: **Avaliação do retinol em parturientes com diabetes mellitus gestacional no pós parto imediato.** Universidade Federal do Rio Grande do Norte, 2013.

874. Rodrigues NRD: **Estudo estereológico do efeito da exposição gestacional à poluição ambiental de São Paulo sobre o desenvolvimento renal em camundongos.** Universidade de São Paulo.

875. Santos AR: Identificação de biomarcadores na fibrogênese pulmonar da paracoccidioidomicose. 2018.

876. Schön L-V: **Der Einfluss des Fettstoffwechsels auf Diabetiker.** uniwien, 2009.

877. Seligman BGS: Efeitos de dieta e exercício sobre a função endotelial e risco cardiovascular em pacientes com síndrome metabólica: ensaio clínico randomizado. 2009.

878. Shamansurova Akhmedova Z: Déterminer les mécanismes impliqués dans les effets du récepteur à la rénine et prorénine dans l’obésité et dans le diabète= Determining mechanisms implicated in the effects of the renin and prorenin receptor in the development of obesity and diabetes. 2017.

879. Sharafi P: Olgun Yağ Hücrelerinin Rekombinant Protein Ifade Kapasitelerinin İncelenmesi. 2013.

880. Silva OSd: **Ioga e repercussões no metabolismo.** 2015.

881. Skrsypcsak C, Locatelli C: Efeitos da liraglutida sobre a glicemia e a obesidade: uma revisão bibliográfica. Vita et Sanitas. 2013;7:51-64.

882. Tao G, Yu W, Siming T, Shuai S, Hongyan C, Ping Y, Yunzhu P: GW24-e0779 Extracorporeal cardiac shock wave therapy (CSWT) for treatment of coronary artery disease. Heart. 2013;99:A158-A159.

883. UNIFACVEST CU, MATOS BAM: INFLUÊNCIA DA PUBLICIDADE ALIMENTÍCIA NA OBESIDADE INFANTIL.

884. Villaescusa MBP: PROGRAMA OFICIAL DE DOCTORADO EN NUTRICIÓN Y CIENCIA DE LOS ALIMENTOS.

885. Voller H, Salzwedel A, Reibis R, Kaminski S, Buhlert H, Eichler S, Wegscheider K: D Mesquita1, A Abreu1, G Portugal1, S Rosa1, P Rio1, M Oliveira1, PS Cunha1, V Santos2, H Santa-Clara2, R Ferreira1 1Hospital de Santa Marta, Lisbon, Portugal, 2Human Motricity Faculty, Lisbon University, Lisbon, Portugal.

886. White PAS: Efeitos do extrato aquoso de abajeru (Crhysobalanus icaco) sobre o peso corporal, adiposidade e sensibilidade à insulina de camundongos obesos. 2011.

887. Wright CV, Dunn NR: Adrian Kee Keong Teo, Norihiro Tsuneyoshi, Shawn Hoon, 2 Ee Kim Tan, Lawrence W. Stanton, 3.

888. XIE F-y, CHEN Z, DING Z, FAN P-y, WANG X, FENG Y, MA G-s: Elevated plasma levels of retinol-binding protein-4 are associated coronary artery disease and one-year prognosis. Modern Medical Journal. 2012;06.

889. Yang H, Wang S, Yan L, Qian P, Duan H: Association of interleukin gene polymorphisms with the risk of coronary artery disease. Genet Mol Res. 2015;14:12489-12496.

890. Αναγνώστου Π: **Εξόρυξη γνώσης και οπτικοποίηση δικτύων μοριακής βιολογίας.** 2019.

891. Γαστουνιώτη Α: Υπολογιστική ανάλυση μηχανικών χαρακτηριστικών του αρτηριακού τοιχώματος από απεικονιστικές καταγραφές με στόχο την υποβοήθηση της διάγνωσης της καρωτιδικής αθηρωμάτωσης. 2015.

892. Δολιανίτη ΛΕ: Το μεταβολικό σύνδρομο σε σχέση με τη φυσική δραστηριότητα σε παιδιά και γονείς του δήμου νότιας Κυνουρίας. 2014.

893. АНЖ ВЕРОНИК БЭ, АКСЕЛЬРОД А, ЩЕКОЧИХИН Д, ТЕБЕНЬКОВА E, ЖЕЛАНКИН А, СТОНОГИНА Д, СЫРКИНА Е, ТЕРНОВОЙ С: Современный алгоритм диагностики ишемической болезни сердца: достижения и перспективы. Kardiologia i Serdechno-Sosudistaya Khirurgia. 2019;12.

894. Билонг ЭАВН, Аксельрод А, Щекочихин Д, Тебенькова E, Стоногина Д, Желанкин А, Сыркина Е, Терновой С: Современный алгоритм диагностики ишемической болезни сердца: достижения и перспективы. Кардиология и сердечно-сосудистая хирургия. 2019;12:418-428.

895. БОННИ К: НОВЫЕ КОНСТРУКЦИИ ТРАНСПОРТЕРОВ И МОЛЕКУЛЫ-КОНЪЮГАТЫ, ЯВЛЯЮЩИЕСЯ ТРАНСПОРТЕРАМИ КАРГО-МОЛЕКУЛ. 2015.

896. БОННИ К: ЭФФЕКТИВНЫЙ ТРАНСПОРТ В ЛЕЙКОЦИТЫ. 2015.

897. Вербовой АФ, Митрошина Е: Адипокины и сердечно-сосудистая система. Эндокринология: Новости Мнения Обучение. 2014.

898. Вороненко Н: Метаболічний синдром та дисфункція жирової тканини у жінок. Здоровье женщины. 2013;65-71.

899. Громова О, Торшин И, Тетруашвили Н, Коденцова В, Рудаков К: Витамин А в акушерстве: фундаментальные и клинические исследования. Медицинский алфавит. 2019;1:59-69.

900. Іванова КВ: **Роль фактора росту фібробластів 21 в регуляції метаболізму кісткової тканини та вуглеводного гомеостазу у хворих на ішемічну хворобу серця з ожирінням.** 2021.

901. ФОТИН-МЛЕЧЕК М, Зёнке Ф: КОМПОЗИЦИЯ, ВКЛЮЧАЮЩАЯ КОМПЛЕКСНУЮ (И) РНК И СВОБОДНУЮ ИРНК ДЛЯ ОСУЩЕСТВЛЕНИЯ ИЛИ ПОВЫШЕНИЯ ИММУНОСТИМУЛИРУЮЩЕГО ОТВЕТА У МЛЕКОПИТАЮЩИХ, И ЕЕ ПРИМЕНЕНИЯ. 2015.

902. ФОТИНМЛЕЧЕК М, БАУМХОФ П: КОМПЛЕКСЫ НА ОСНОВЕ РНК И КАТИОННЫХ ПЕПТИДОВ ДЛЯ ТРАНСФЕКЦИИ И ИММУНОСТИМУЛЯЦИИ. 2013.

903. اکبرزاده: بررسي تغييرات سرمي رتينول باندينگ پروتئين4، واسپين، ويسفاتين، اديپونكتين، امنتين-1، APO-B100و LDLاكسيده در افراد65-25ساله خانواده هاي ديابتي شهر بوشهر.

904. رمضانی, آتنا: اثرات ویتامین D بر پروتئین آدیپونکتین و التهاب: یک مرور ساده. تعالی بالینی. 2016;5:50-64.

905. سوری, رحمان, چوپانی, ثروت, فلاحیان, خانی ر: تاثیر فعالیت بدنی بر سطوح هموسیستئین سرم زنان چاق و دارای اضافه وزن. مجله علمی پژوهشی افق دانش. 2016;22:307-312.

906. 김지훈, 이은정, 최은숙, 원종철, 박철영, 이원영, 오기원, 김병진, 성기철, 김범수: 한국인 성인에서 관상동맥질환과 혈중 Retinol-Binding Protein 4 농도와의 연관성. Korean Diabetes Journal. 2009;33:105-112.

907. 侯晓雯, 李光, 赵琼蕊, 陈欣, 王冲, 时景璞: 中国人视黄醇结合蛋白 4 水平与冠心病关系的 Meta 分析. 中华流行病学杂志. 2015;36:1010-1014.

908. 保科克行, 加藤雅明, 根本卓, 須原正光, 白須拓郎, 芳賀真, 望月康晃, 根元洋光, 松倉満, 赤井隆文: 大動脈ステントグラフトの branch protection: 最善の手法とは? 2013.

909. 关于, 查看全文GydF4y2BaGydF4y2Ba, 运用在, 运用在GydF4y2BaGydF4y2Ba, 图库GydF4y2BaAudioslides, 介绍视频和褒奖GydF4y2BaENM: 脂肪因子与非酒精性脂肪肝发生, 发展的关系 GydF4y2Ba.

910. 刘佟: **炎症脂肪细胞因子与冠状动脉粥样硬化性心脏病的相关性.** 承德医学院, 2019.

911. 吳令怡, 陳介甫, 黃翊恭: 肥胖相關代謝症候群與減肥藥. J Chin Med. 2013;24:261-277.

912. 小畑淳史, 窪田直人, 窪田哲也, 佐藤寛之, 桜井賛孝, 岩本真彦, 深澤正徳, 鈴木昌幸, 本田清史, 鈴木好幸: 的変化―SGLT2 阻害薬と DPPIV 阻害薬との比較―.

913. 徐建华, 高运泽, 张玉风, 张红: 利多卡因在搭桥术中抑制再灌注室颤研究. 医药论坛杂志. 2004;25:14-15.

914. 李瑶瑶, 董文鹏, 陆华, 童光, 王晓武, 马涛, 张卫达: 血清 Leptin, SFRP5, RBP4 与冠心病的相关性分析. 安徽医科大学学报. 2016;51:1160-1163.

915. 李非: **视黄醇结合蛋白 4 促高胰岛素血症血管平滑肌增殖作用及机制的研究.** 中南大学, 2013.

916. 李非, 杨天伦, 赵震宇, 夏珂: **冠心病患者血浆视黄醇结合蛋白 4 水平变化及高胰岛素血症对其的影响.** 2012.

917. 桐林美緒, 越前宏俊: HMG-CoA 還元酵素阻害薬による脂質異常症の治療モニタリングにおける高感度 CRP 濃度測定の意義と耐糖能異常の影響に関するメタ解析. 医療薬学. 2012;38:479-490.

918. 牛善利, 黄友敏, 周永勤: 突发性耳聋患者内脂素和 RBP4 含量变化的研究. 中国医师杂志. 2012;14:465-467.

919. 王珊, 姚娟, 高小平: 急性脑卒中患者血清视黄醇结合蛋白 4 的表达及其与低密度脂蛋白关系的探讨. 湖南师范大学学报: 医学版. 2013;45-47.

920. 王红霞: **冠心病患者血清视黄醇结合蛋白 4 (RBP4) 和性激素水平的检测分析.** 南京医科大学, 2019.

921. 秦辛玲, 石青峰, 汤希凡: 妊娠高血压综合征患者血清视黄醇结合蛋白 4 的水平变化. 中国现代医学杂志. 2015;25:58-59.

922. 董文锡, 申学舟, 高凌云, 张其敏, 张硅, 尹海辉, 葛文, 许崇永: MSCT 多维重组技术对小儿先天性肺动脉狭窄的诊断价值. 中国医师杂志. 2012;14:468-472.

923. 詹唯玮, 陈红青, 张素英, 张俊光: 胰岛素泵双波疗法联合健康教育治疗妊娠期糖尿病对孕妇血清铁蛋白及视黄醇结合蛋白 4 水平的影响分析. 解放军预防医学杂志. 2016;102-102.

924. 许明星: 建筑工程高支模支撑体系配置与计算. 建筑知识: 学术刊. 2011;73-75.

925. Llombart V, Garcia-Berrocoso T, Bustamante A, Giralt D, Rodriguez-Luna D, Muchada M, Penalba A, Boada C, Hernandez-Guillamon M, Montaner J: Plasmatic retinol-binding protein 4 and glial fibrillary acidic protein as biomarkers to differentiate ischemic stroke and intracerebral hemorrhage. Journal of Neurochemistry. 2016;136:416-424.

926. Rahim MAA, Rahim ZHA, Ahmad WAW, Bakri MM, Ismail MD, Hashim OH: Inverse changes in plasma tetranectin and titin levels in patients with type 2 diabetes mellitus: a potential predictor of acute myocardial infarction? Acta Pharmacol Sin. 2018;39:1197-1207.

927. von Eynatten M, Breitling LP, Roos M, Baumann M, Rothenbacher D, Brenner H: Circulating adipocyte fatty acid-binding protein levels and cardiovascular morbidity and mortality in patients with coronary heart disease: a 10-year prospective study. Arterioscler Thromb Vasc Biol. 2012;32:2327-2335.

928. Cheow ESH, Cheng WC, Yap T, Dutta B, Lee CN, Kleijn DPV, Sorokin V, Sze SK: Myocardial Injury Is Distinguished from Stable Angina by a Set of Candidate Plasma Biomarkers Identified Using iTRAQ/MRM-Based Approach. J Proteome Res. 2018;17:499-515.

929. Dong H, Li X, Tang Y: Serum Retinol-Binding Protein-4 Level is a High Risk Factor for Coronary Heart Disease in Chinese. Clin Lab. 2015;61:1675-1678.

930. Stakhneva EM, Meshcheryakova IA, Demidov EA, Starostin KV, Peltek SE, Voevoda MI, Ragino YI: Changes in the proteomic profile of blood serum in coronary atherosclerosis. Journal of Medical Biochemistry. 2019.

931. Ingelsson E, Lind L: Circulating retinol-binding protein 4 and subclinical cardiovascular disease in the elderly. Diabetes Care. 2009;32:733-735.

932. Ingelsson E, Sundström J, Melhus H, Michaëlsson K, Berne C, Vasan RS, Risérus U, Blomhoff R, Lind L, Arnlöv J: Circulating retinol-binding protein 4, cardiovascular risk factors and prevalent cardiovascular disease in elderly. Atherosclerosis. 2009;206:239-244.

933. Kim JM, Won JC, Ko KS, Rhee BD, Park CY: Relationship between retinol binding protein 4 and the risk of cardiovascular diseases. Diabetologia. 2011;54:S300.

934. Alkharfy KM, Al-Daghri NM, Vanhoutte PM, Krishnaswamy S, Xu A: Serum retinol-binding protein 4 as a marker for cardiovascular disease in women. PLoS One. 2012;7:e48612.

935. Pala L, Monami M, Ciani S, Dicembrini I, Pasqua A, Pezzatini A, Francesconi P, Cresci B, Mannucci E, Rotella CM: Adipokines as possible new predictors of cardiovascular diseases: a case control study. J Nutr Metab. 2012;2012:253428.

936. García-Fontana B, Morales-Santana S, Longobardo V, Reyes-García R, Rozas-Moreno P, García-Salcedo JA, Muñoz-Torres M: Relationship between Proinflammatory and Antioxidant Proteins with the Severity of Cardiovascular Disease in Type 2 Diabetes Mellitus. Int J Mol Sci. 2015;16:9469-9483.

937. Patterson CC, Blankenberg S, Ben-Shlomo Y, Heslop L, Bayer A, Lowe G, Zeller T, Gallacher J, Young I, Yarnell J: Which biomarkers are predictive specifically for cardiovascular or for non-cardiovascular mortality in men? Evidence from the Caerphilly Prospective Study (CaPS). Int J Cardiol. 2015;201:113-118.

938. Liu G, Ding M, Chiuve SE, Rimm EB, Franks PW, Meigs JB, Hu FB, Sun Q: Plasma Levels of Fatty Acid-Binding Protein 4, Retinol-Binding Protein 4, High-Molecular-Weight Adiponectin, and Cardiovascular Mortality Among Men With Type 2 Diabetes: A 22-Year Prospective Study. Arterioscler Thromb Vasc Biol. 2016;36:2259-2267.

939. Rist PM, Jiménez MC, Tworoger SS, Hu FB, Manson JE, Sun Q, Rexrode KM: Plasma Retinol-Binding Protein 4 Levels and the Risk of Ischemic Stroke among Women. J Stroke Cerebrovasc Dis. 2018;27:68-75.

940. Wong YK, Cheung CYY, Tang CS, Au KW, Hai JSH, Lee CH, Lau KK, Cheung BMY, Sham PC, Xu A, et al: Age-Biomarkers-Clinical Risk Factors for Prediction of Cardiovascular Events in Patients With Coronary Artery Disease. Arterioscler Thromb Vasc Biol. 2018;38:2519-2527.

941. Zhu YY, Zhang JL, Liu L, Han Y, Ge X, Zhao S: Evaluation of serum retinol-binding protein-4 levels as a biomarker of poor short-term prognosis in ischemic stroke. Biosci Rep. 2018;38.

942. Cheng X, Wu Z, Yuan B: Diagnostic and predictive value of serum LDL/HDL and RBP4 levels in restenosis after revascularization in patients with coronary heart disease (CHD). Int J Clin Exp Med. 2019;12:10783-10788.

943. Liu C, Che Y: Retinol-binding protein 4 predicts lesion volume (determined by MRI) and severity of acute ischemic stroke. Neurotoxicity research. 2019;35:92-99.

944. Nar G, Sanlialp SC: Retinol Binding Protein 4 Levels relate to the presence and severity of coronary artery disease. Journal of Medical Biochemistry. 2021.

945. Salgado-Somoza A, Teijeira-Fernandez E, Rubio J, Couso E, Gonzalez-Juanatey JR, Eiras S: Coronary artery disease is associated with higher epicardial retinol-binding protein 4 (RBP4) and lower glucose transporter (GLUT) 4 levels in epicardial and subcutaneous adipose tissue. Clin Endocrinol (Oxf). 2012;76:51-58.

**Supplementary table 4.** Risk of bias assessment using the Risk of Bias in Non-randomized Studies – of Exposures

| References (Author, Year) | D1 | D2 | D3 | D4 | D5 | D6 | D7 | Overall |
| --- | --- | --- | --- | --- | --- | --- | --- | --- |
| von Eynatten et al., 2007[31] | 2 | 2 | 1 | 1 | 1 | 1 | 1 | 2-Moderate |
| Mallat et al., 2009[35] | 2 | 1 | 2 | 1 | 1 | 1 | 1 | 2-Moderate |
| Al-Daghri et al., 2009[10] | 2 | 2 | 1 | 1 | 1 | 1 | 1 | 2-Moderate |
| Mahmoudi et al., 2012[32] | 2 | 2 | 1 | 1 | 1 | 1 | 1 | 2-Moderate |
| Sun et al., 2013[33] | 2 | 1 | 1 | 1 | 1 | 1 | 1 | 2-Moderate |
| Cubedo et al., 2014[12] | 2 | 2 | 1 | 1 | 1 | 1 | 1 | 2-Moderate |
| Lambadiari et al., 2014[7] | 2 | 2 | 1 | 1 | 1 | 1 | 1 | 2-Moderate |
| Li et al., 2014[34] | 2 | 2 | 1 | 1 | 1 | 1 | 1 | 2-Moderate |
| Liu et al., 2015[8] | 2 | 1 | 1 | 1 | 1 | 1 | 1 | 2-Moderate |
| Guan et al., 2016[11] | 2 | 2 | 1 | 1 | 1 | 1 | 1 | 2-Moderate |
| Wang et al., 2018[15] | 2 | 2 | 1 | 1 | 1 | 1 | 1 | 2-Moderate |
| Liu et al., 2019[14] | 2 | 2 | 1 | 1 | 1 | 1 | 1 | 2-Moderate |
| Sun et al., 2019[9] | 2 | 2 | 1 | 1 | 1 | 1 | 1 | 2-Moderate |
| Pan et al., 2020[13] | 2 | 2 | 1 | 1 | 1 | 1 | 1 | 2-Moderate |
| Si et al., 2020[36] | 2 | 2 | 2 | 1 | 1 | 1 | 1 | 2-Moderate |

D: Domain; D1: Bias due to confounding; D2: Bias in selection of participants into the study; D3: Bias in classification of exposures; D4: Bias due to departures from intended exposures; D5: Bias due to missing data; D6: Bias in measurement of outcomes; D7: Bias in selection of the reported result; Overall. Risk of bias assessment: 0—No information; 1—Low; 2—Moderate; 3—Serious; 4—Critical.

**Supplementary table 5.** Sensitivity analysis for circulating RBP4 levels in coronary artery disease (CAD) patients and controls in included studies.

| Excluded study | Pooled effect | LCI 95% | HCI 95% | Cochran Q | *P* | I^2^ | I^2^ LCI 95% | I^2^ HCI 95% |
| --- | --- | --- | --- | --- | --- | --- | --- | --- |
| von Eynatten et al., 2007[31] | 0.28 | -0.28 | 0.84 | 408.46 | 0.00 | 96.33 | 95.14 | 97.22 |
| Mallat et al., 2009[35] | 0.34 | -0.16 | 0.84 | 414.53 | 0.00 | 96.38 | 95.22 | 97.26 |
| Al-Daghri et al., 2009[10] | 0.25 | -0.30 | 0.79 | 422.79 | 0.00 | 96.45 | 95.32 | 97.31 |
| Mahmoudi et al., 2012[32] | 0.25 | -0.29 | 0.80 | 430.02 | 0.00 | 96.51 | 95.41 | 97.35 |
| Sun et al., 2013[33] | 0.28 | -0.38 | 0.95 | 421.40 | 0.00 | 96.44 | 95.31 | 97.30 |
| Cubedo et al., 2014[12] | 0.28 | -0.26 | 0.82 | 387.53 | 0.00 | 96.13 | 94.86 | 97.09 |
| Lambadiari et al., 2014[7] | 0.21 | -0.32 | 0.75 | 367.04 | 0.00 | 95.91 | 94.55 | 96.94 |
| Li et al., 2014[34] | 0.26 | -0.29 | 0.80 | 429.11 | 0.00 | 96.50 | 95.40 | 97.34 |
| Li et al., 2014[34] | 0.25 | -0.30 | 0.80 | 429.91 | 0.00 | 96.51 | 95.41 | 97.35 |
| Liu et al., 2015[8] | 0.20 | -0.33 | 0.73 | 344.30 | 0.00 | 95.64 | 94.15 | 96.75 |
| Guan et al., 2016[11] | 0.25 | -0.30 | 0.80 | 429.91 | 0.00 | 96.51 | 95.41 | 97.35 |
| Guan et al., 2016[11] | 0.24 | -0.29 | 0.77 | 405.79 | 0.00 | 96.30 | 95.11 | 97.21 |
| Wang et al., 2018[15] | 0.30 | -0.30 | 0.90 | 392.47 | 0.00 | 96.18 | 94.93 | 97.12 |
| Liu et al., 2019[14] | 0.23 | -0.32 | 0.78 | 397.89 | 0.00 | 96.23 | 95.00 | 97.16 |
| Sun et al., 2019[9] | 0.21 | -0.28 | 0.69 | 318.77 | 0.00 | 95.29 | 93.64 | 96.52 |
| Pan et al., 2020[13] | 0.26 | -0.30 | 0.83 | 425.31 | 0.00 | 96.47 | 95.35 | 97.32 |
| Si et al., 2020[36] | 0.25 | -0.32 | 0.82 | 430.01 | 0.00 | 96.51 | 95.41 | 97.35 |

LCI: low confidential interval; HCL: high confidential interval

**Supplementary table 6.** GRADE certainty of evidence assessment

| **Certainty assessment** | | | | | | | | **Certainty** |
| --- | --- | --- | --- | --- | --- | --- | --- | --- |
| **Outcome** | **No. of studies** | **Study design** | **Risk of bias** | **Inconsistency** | **Indirectness** | **Imprecision** | **Other**  **considerations** |  |
| **RBP4** | 15 | observational studies | not serious | serious^1^ | serious^2^ | not serious | none | ⨁◯◯◯ Very LOW |

^1^ Serious inconsistency as *I^2^*=96.00% and *P*<0.01

^2^ Serious indirectness for mean BMI of patients and complications may affect the generalizability of the results

**Supplementary table 7** Detailed information for 5 studies included in the meta-analysis of pooled odd ratio.

| References (Author, Year) | Country | Study design | Cases/Controls | Subtypes of CAD | Comparison categories | OR/RR (95%CI) | Covariates in the most fully-adjusted model |
| --- | --- | --- | --- | --- | --- | --- | --- |
| Mallat et al., 2008 | France | Case-control | 1036/1889 | CAD | Quartile 4 vs. quartile 1 | 0.93 (0.70-1.24) | body mass index, diabetes, smoking status,  systolic blood pressure, triglycerides, LDL- and HDL-cholesterol, CRP, and adiponectin |
| Sun et al., 2013 | USA | Prospective | 468/472 | CAD | Highest quartile vs. Lowest quartile | 0.74 (0.36-1.50) | age at blood draw, smoking status (never, past, current), fasting status (yes, no), and time of blood draw，body mass index (kg/m2); menopausal status (yes, no); hormone use (current user, past user, and nonuser);  physical activity (MET-h/wk); alcohol use (g/d); parental history of myocardial infarction <65 years of age (yes, no); use of aspirin (yes, no); glomerular filtration rate;  intakes of whole grains, trans fat, and polyunsaturated fat (in tertiles); and history of hypercholesterolemia, diabetes mellitus, or hypertension (yes, no)，total cholesterol (mg/dL), triacylglycerol (mg/dL), high-sensitivity C-reactive protein (mg/L), hemoglobin A1c (%),  and total adiponectin (μg/mL) |
| Liu et al., 2015 | China | Prospective | 201/225 | Stable CAD | Continuous | 1.228 (1.061-1.358) | - |
| Wang et al., 2018 | China | Case-control | 440/218 | CAD | Continuous | 0.975 (0.958-0.993) | - |
| Sun et al., 2019 | China | Case-control | 148/102 | SCH+CAD | Continuous | 1.17 (1.10-1.25) | age, gender, BMI, smoking, hypertension, diabetes, TSH, T3, TC, TG, LDL-C, HDL-C, FBG, UA, and Cr. |

CAD: coronary artery disease; SCH: Subclinical hypothyroidism; DM: diabetes mellitus; SD: standard deviation.

**Supplementary fig. 1** The forest plot for pooled odds ratio between circulating RBP4 and risk of coronary artery disease
